# Supplementary material for: Problematic gaming in occasional, low, intermediate or heavy use gamers – not all screen time is created equal – A reanalysis
Source: PLoS One. 2026 Oct 21;21(10):e0356080. doi: 10.1371/journal.pone.0356080 (PMC13686491; doi:10.1371/journal.pone.0356080)
Supplement: Supplementary file 1 [file pone.0356080.s002.pdf]

S2 File.

Detailed cross-lagged panel model (CLPM) results.

Full output of the cross-lagged panel models summarised in Table S5 and described in section 3.6 of S1 File. One model was estimated for each predictor, with Problematic Gaming as the outcome. Each model reports model significance, model fit, factor loadings, regression paths and residual variances.

These models estimate cross-lagged associations at the between-person level; the within-person decomposition is given in S3 File.

Contents

CLPM: x = VG Hours, y = Problematic Gaming pp. 2-4

CLPM: x = Impulsiveness, y = Problematic Gaming pp. 5-7

CLPM: x = Family Environment, y = Problematic Gaming pp. 8-10

lx1-lx3 = predictor at waves 1-3; ly1-ly3 = Problematic Gaming at waves 1-3.
*Significance markers: * p < .05; ** p < .01; *** p < .001.*

CLPM model:
x = VG Hours, y = Problematic Gaming

Model Significance

N	χ^2	df	p
3333	9.828	2	0.007

Model Fit

CFI	RMSEA	90% CI	TLI	SRMR	AIC	BIC
0.997	0.034	0.015 — 0.057	0.980	0.011	41858.070	42010.861

Factor Loadings

		Standardized					
Latent Factor	Indicator	Loading	95% CI	sig	SE	z	p
lx1	x1	0.635	0.557 — 0.712	***	0.040	16.007	0.000
lx2	x2	0.711	0.652 — 0.770	***	0.030	23.653	0.000
lx3	x3	0.855	0.831 — 0.880	***	0.012	69.160	0.000
ly1	y1	0.799	0.728 — 0.871	***	0.036	22.014	0.000
ly2	y2	0.786	0.737 — 0.834	***	0.025	31.809	0.000
ly3	y3	0.836	0.818 — 0.854	***	0.009	90.556	0.000

* p < .05; ** p < .01; *** p < .001

Regression Paths

		Standardized					
Predictor	DV	β	95% CI	sig	SE	z	p
lx1	lx2	0.827	0.664 — 0.990	***	0.083	9.964	0.000
ly1	lx2	−0.014	−0.130 — 0.103		0.060	−0.229	0.819
lx2	lx3	0.803	0.702 — 0.905	***	0.052	15.518	0.000

ly2	lx3	−0.013	−0.120 — 0.095		0.055	−0.232	0.817
lx1	ly2	−0.025	−0.117 — 0.066		0.047	−0.539	0.590
ly1	ly2	0.776	0.651 — 0.901	***	0.064	12.163	0.000
lx2	ly3	−0.026	−0.119 — 0.067		0.047	−0.548	0.583
ly2	ly3	0.770	0.688 — 0.852	***	0.042	18.455	0.000

* p < .05; ** p < .01; *** p < .001

Latent Factor Correlations

Factor	Factor	r	95% CI	sig	SE	p
lx1	ly1	0.586	0.498 — 0.673	***	0.045	0.000
lx2	ly2	0.656	0.405 — 0.908	***	0.128	<0.001
lx3	ly3	0.416	0.269 — 0.564	***	0.075	<0.001

* p < .05; ** p < .01; *** p < .001

Latent Factor Variance/Residual Variance

Factor	Variance	Std. Variance	sig	p
lx2	0.151	0.329	**	0.004
lx3	0.203	0.367	***	0.000
ly2	0.281	0.420	***	<0.001
ly3	0.307	0.430	***	0.000
lx1	0.400	1.000	***	<0.001
ly1	0.635	1.000	***	0.000

Explained Variance

Variable	R^2
lx2	0.671

lx3	0.633
ly2	0.580
ly3	0.570

CLPM model:

x = Impulsiveness, y = Problematic Gaming

Model Significance

N	χ^2	df	p
3291	2.485	2	0.289

Model Fit

CFI	RMSEA	90% CI	TLI	SRMR	AIC	BIC
1.000	0.009	0.000 — 0.037	0.999	0.004	42568.512	42720.986

Factor Loadings

		Standardized					
Latent Factor	Indicator	Loading	95% CI	sig	SE	z	p
lx1	x1	0.685	0.626 — 0.745	***	0.030	22.585	0.000
lx2	x2	0.700	0.656 — 0.744	***	0.022	31.296	0.000
lx3	x3	0.702	0.677 — 0.728	***	0.013	54.313	0.000
ly1	y1	0.774	0.707 — 0.842	***	0.034	22.498	0.000
ly2	y2	0.775	0.728 — 0.822	***	0.024	32.468	0.000
ly3	y3	0.693	0.668 — 0.718	***	0.013	54.941	0.000

* p < .05; ** p < .01; *** p < .001

Regression Paths

		Standardized					
Predictor	DV	β	95% CI	sig	SE	z	p
lx1	lx2	0.879	0.758 — 1.000	***	0.062	14.232	0.000
ly1	lx2	−0.047	−0.137 — 0.042		0.046	−1.042	0.297
lx2	lx3	0.975	0.898 — 1.052	***	0.039	24.833	0.000

ly2	lx3	−0.049	−0.139 — 0.041		0.046	−1.077	0.282
lx1	ly2	−0.009	−0.086 — 0.068		0.039	−0.231	0.817
ly1	ly2	0.788	0.673 — 0.902	***	0.058	13.513	0.000
lx2	ly3	−0.011	−0.108 — 0.085		0.049	−0.233	0.816
ly2	ly3	0.930	0.849 — 1.012	***	0.041	22.432	0.000

* p < .05; ** p < .01; *** p < .001

Latent Factor Correlations

Factor	Factor	r	95% CI	sig	SE	p
lx1	ly1	0.541	0.462 — 0.619	***	0.040	0.000
lx2	ly2	0.739	0.470 — 1.008	***	0.137	<0.001
lx3	ly3	1.926	1.094 — 2.758	***	0.425	<0.001

* p < .05; ** p < .01; *** p < .001

Latent Factor Variance/Residual Variance

Factor	Variance	Std. Variance	sig	p
lx2	0.154	0.270	**	0.003
lx3	0.057	0.101	**	0.004
ly2	0.248	0.387	***	<0.001
ly3	0.072	0.146	***	<0.001
lx1	0.470	1.000	***	0.000
ly1	0.598	1.000	***	0.000

Explained Variance

Variable	R^2
lx2	0.730

CLPM model:

x = Impulsiveness, y = Problematic Gaming

lx3	0.899
ly2	0.613
ly3	0.854

CLPM model:

x = Family Environment, y = Problematic Gaming

Model Significance

N	χ^2	df	p
3293	1.738	2	0.419

Model Fit

CFI	RMSEA	90% CI	TLI	SRMR	AIC	BIC
1.000	0.000	0.000 — 0.033	1.001	0.005	42028.601	42181.090

Factor Loadings

		Standardized					
Latent Factor	Indicator	Loading	95% CI	sig	SE	z	p
lx1	x1	0.702	0.640 — 0.764	***	0.032	22.039	0.000
lx2	x2	0.718	0.672 — 0.763	***	0.023	30.783	0.000
lx3	x3	0.707	0.681 — 0.732	***	0.013	54.103	0.000
ly1	y1	0.770	0.702 — 0.838	***	0.035	22.190	0.000
ly2	y2	0.770	0.722 — 0.818	***	0.024	31.455	0.000
ly3	y3	0.696	0.671 — 0.720	***	0.012	55.673	0.000

* p < .05; ** p < .01; *** p < .001

Regression Paths

		Standardized					
Predictor	DV	β	95% CI	sig	SE	z	p
lx1	lx2	0.881	0.771 — 0.991	***	0.056	15.680	0.000
ly1	lx2	0.045	−0.029 — 0.119		0.038	1.184	0.237
lx2	lx3	0.966	0.906 — 1.026	***	0.030	31.708	0.000

ly2	lx3	0.047	−0.028 — 0.123		0.039	1.230	0.219
lx1	ly2	−0.039	−0.101 — 0.024		0.032	−1.214	0.225
ly1	ly2	0.775	0.676 — 0.874	***	0.050	15.358	0.000
lx2	ly3	−0.047	−0.125 — 0.031		0.040	−1.173	0.241
ly2	ly3	0.907	0.844 — 0.970	***	0.032	28.229	0.000

* p < .05; ** p < .01; *** p < .001

Latent Factor Correlations

Factor	Factor	r	95% CI	sig	SE	p
lx1	ly1	−0.458	−0.540 — −0.376	***	0.042	0.000
lx2	ly2	−0.397	−0.646 — −0.149	**	0.127	0.002
lx3	ly3	−0.869	−1.535 — −0.203	*	0.340	0.011

* p < .05; ** p < .01; *** p < .001

Latent Factor Variance/Residual Variance

Factor	Variance	Std. Variance	sig	p
lx2	0.146	0.258	**	0.004
lx3	0.056	0.104	**	0.001
ly2	0.235	0.370	***	<0.001
ly3	0.068	0.138	***	<0.001
lx1	0.494	1.000	***	0.000
ly1	0.593	1.000	***	0.000

Explained Variance

Variable	R^2
lx2	0.742

CLPM model:

x = Family Environment, y = Problematic Gaming

lx3	0.896
ly2	0.630
ly3	0.862
