# Supplementary material for: Problematic gaming in occasional, low, intermediate or heavy use gamers – not all screen time is created equal – A reanalysis
Source: PLoS One. 2026 Oct 21;21(10):e0356080. doi: 10.1371/journal.pone.0356080 (PMC13686491; doi:10.1371/journal.pone.0356080)
Supplement: Supplementary file 2 [file pone.0356080.s003.pdf]

S3 File.

Detailed random-intercept cross-lagged panel model (RI-CLPM) results.

Full output of the random-intercept cross-lagged panel models summarised in Table S5 and S7 Fig, and described in section 3.6 of S1 File. One model was estimated for each predictor, with Problematic Gaming as the outcome. Each model reports model significance, model fit, factor loadings, regression paths and residual variances.

By including random intercepts, these models separate stable between-person differences from within-person change, so the regression paths reported here are within-person cross-lagged effects.

Contents

RI-CLPM:	x = VG Hours, y = Problematic Gaming	pp. 2-4
RI-CLPM:	x = Impulsiveness, y = Problematic Gaming	pp. 5-7
RI-CLPM:	x = Family Environment, y = Problematic Gaming	pp. 8-10

lx1-lx3 = predictor at waves 1-3; ly1-ly3 = Problematic Gaming at waves 1-3.
ix, iy = random intercepts (between-person) for predictor and outcome.
*Significance markers: * p < .05; ** p < .01; *** p < .001.*

RI-CLPM model:

x = VG Hours, y = Problematic Gaming

Model Significance

N	χ^2	df	p
3333	43.716	7	<0.001

Model Fit

CFI	RMSEA	90% CI	TLI	SRMR	AIC	BIC
0.988	0.040	0.029 — 0.051	0.974	0.030	41881.958	42004.190

Factor Loadings

Latent Factor	Indicator	Standardized Loading	95% CI	sig	SE	z	p
ix	x1	0.586	0.548 — 0.624	***	0.019	30.602	0.000
ix	x2	0.663	0.617 — 0.709	***	0.023	28.212	0.000
ix	x3	0.663	0.617 — 0.709	***	0.023	28.223	0.000
iy	y1	0.618	0.573 — 0.662	***	0.023	27.126	0.000
iy	y2	0.609	0.563 — 0.656	***	0.024	25.652	0.000
iy	y3	0.609	0.562 — 0.656	***	0.024	25.392	0.000
lx1	x1	0.810	0.783 — 0.837	***	0.014	58.514	0.000
lx2	x2	0.749	0.708 — 0.790	***	0.021	36.035	0.000
lx3	x3	0.749	0.708 — 0.790	***	0.021	36.052	0.000
ly1	y1	0.786	0.751 — 0.821	***	0.018	43.944	0.000
ly2	y2	0.793	0.757 — 0.829	***	0.018	43.459	0.000
ly3	y3	0.793	0.757 — 0.829	***	0.018	43.100	0.000

* p < .05; ** p < .01; *** p < .001

RI-CLPM model:

x = VG Hours, y = Problematic Gaming

Regression Paths

		Standardized					
**Predictor**	**DV**	**β**	**95% CI**	**sig**	**SE**	**z**	**p**
lx1	lx2	−0.005	−0.100 — 0.091		0.049	−0.099	0.921
ly1	lx2	0.035	−0.044 — 0.114		0.040	0.866	0.387
lx2	lx3	−0.004	−0.082 — 0.074		0.040	−0.099	0.921
ly2	lx3	0.036	−0.046 — 0.117		0.042	0.860	0.390
lx1	ly2	−0.055	−0.121 — 0.011		0.034	−1.647	0.100
ly1	ly2	0.172	0.093 — 0.252	***	0.041	4.249	<0.001
lx2	ly3	−0.045	−0.098 — 0.008		0.027	−1.665	0.096
ly2	ly3	0.176	0.093 — 0.259	***	0.042	4.157	<0.001

* p < .05; ** p < .01; *** p < .001

Latent Factor Correlations

Factor	Factor	r	95% CI	sig	SE	p
ix	iy	0.612	0.520 — 0.704	***	0.047	0.000
lx1	ly1	0.163	0.105 — 0.221	***	0.029	<0.001
lx2	ly2	0.093	−0.001 — 0.187		0.048	0.052
lx3	ly3	0.187	0.111 — 0.263	***	0.039	<0.001

* p < .05; ** p < .01; *** p < .001

Latent Factor Variance/Residual Variance

Factor	Variance	Std. Variance	sig	p
lx1	0.681	1.000	***	0.000
lx2	0.455	0.999	***	0.000

lx3	0.455	0.999	***	0.000
ly1	0.624	1.000	***	0.000
ly2	0.633	0.970	***	0.000
ly3	0.633	0.968	***	0.000
ix	0.356	1.000	***	0.000
iy	0.385	1.000	***	0.000

Explained Variance

Variable	R^2
lx2	0.001
lx3	0.001
ly2	0.030
ly3	0.032

RI-CLPM model:

x = Impulsiveness, y = Problematic Gaming

Model Significance

N	χ^2	df	p
3291	16.515	7	0.021

Model Fit

CFI	RMSEA	90% CI	TLI	SRMR	AIC	BIC
0.997	0.020	0.007 — 0.033	0.993	0.015	42572.542	42694.521

Factor Loadings

Latent Factor	Indicator	Standardized					
		Loading	95% CI	sig	SE	z	p
ix	x1	0.631	0.592 — 0.670	***	0.020	31.928	0.000
ix	x2	0.596	0.555 — 0.637	***	0.021	28.556	0.000
ix	x3	0.595	0.554 — 0.637	***	0.021	28.053	0.000
iy	y1	0.610	0.563 — 0.658	***	0.024	25.240	0.000
iy	y2	0.599	0.551 — 0.647	***	0.025	24.389	0.000
iy	y3	0.598	0.550 — 0.647	***	0.025	24.037	0.000
lx1	x1	0.776	0.744 — 0.807	***	0.016	48.230	0.000
lx2	x2	0.803	0.773 — 0.833	***	0.015	51.872	0.000
lx3	x3	0.804	0.773 — 0.834	***	0.016	51.153	0.000
ly1	y1	0.792	0.755 — 0.829	***	0.019	42.484	0.000
ly2	y2	0.801	0.765 — 0.837	***	0.018	43.552	0.000
ly3	y3	0.801	0.765 — 0.838	***	0.019	43.099	0.000

* p < .05; ** p < .01; *** p < .001

RI-CLPM model:

x = Impulsiveness, y = Problematic Gaming

Regression Paths

		Standardized					
**Predictor**	**DV**	**β**	**95% CI**	**sig**	**SE**	**z**	**p**
lx1	lx2	0.089	0.022 — 0.156	**	0.034	2.593	0.010
ly1	lx2	0.095	0.038 — 0.151	***	0.029	3.292	<0.001
lx2	lx3	0.097	0.023 — 0.172	*	0.038	2.566	0.010
ly2	lx3	0.097	0.039 — 0.156	**	0.030	3.266	0.001
lx1	ly2	0.051	−0.002 — 0.104		0.027	1.879	0.060
ly1	ly2	0.172	0.093 — 0.251	***	0.040	4.273	<0.001
lx2	ly3	0.056	−0.003 — 0.115		0.030	1.871	0.061
ly2	ly3	0.177	0.094 — 0.259	***	0.042	4.210	<0.001

* p < .05; ** p < .01; *** p < .001

Latent Factor Correlations

Factor	Factor	r	95% CI	sig	SE	p
ix	iy	0.485	0.400 — 0.571	***	0.044	0.000
lx1	ly1	0.161	0.099 — 0.223	***	0.032	<0.001
lx2	ly2	0.214	0.148 — 0.279	***	0.033	<0.001
lx3	ly3	0.222	0.170 — 0.274	***	0.027	0.000

* p < .05; ** p < .01; *** p < .001

Latent Factor Variance/Residual Variance

Factor	Variance	Std. Variance	sig	p
lx1	0.611	1.000	***	0.000
lx2	0.720	0.980	***	0.000

lx3	0.720	0.977	***	0.000
ly1	0.628	1.000	***	0.000
ly2	0.643	0.965	***	0.000
ly3	0.643	0.961	***	0.000
ix	0.404	1.000	***	0.000
iy	0.373	1.000	***	0.000

Explained Variance

**Variable**	**R^2**
lx2	0.020
lx3	0.023
ly2	0.035
ly3	0.039

RI-CLPM model:

x = Family Environment, y = Problematic Gaming

Model Significance

N	χ^2	df	p
3293	13.643	7	0.058

Model Fit

CFI	RMSEA	90% CI	TLI	SRMR	AIC	BIC
0.998	0.017	0.000 — 0.030	0.995	0.013	42030.506	42152.497

Factor Loadings

		Standardized					
Latent Factor	Indicator	Loading	95% CI	sig	SE	z	p
ix	x1	0.632	0.594 — 0.670	***	0.020	32.361	0.000
ix	x2	0.612	0.571 — 0.653	***	0.021	29.086	0.000
ix	x3	0.612	0.570 — 0.653	***	0.021	28.767	0.000
iy	y1	0.613	0.566 — 0.659	***	0.024	25.587	0.000
iy	y2	0.602	0.554 — 0.651	***	0.025	24.339	0.000
iy	y3	0.602	0.553 — 0.651	***	0.025	24.050	0.000
lx1	x1	0.775	0.744 — 0.806	***	0.016	48.684	0.000
lx2	x2	0.791	0.759 — 0.823	***	0.016	48.532	0.000
lx3	x3	0.791	0.759 — 0.823	***	0.016	48.086	0.000
ly1	y1	0.790	0.754 — 0.827	***	0.019	42.614	0.000
ly2	y2	0.798	0.762 — 0.835	***	0.019	42.736	0.000
ly3	y3	0.799	0.762 — 0.836	***	0.019	42.337	0.000

* p < .05; ** p < .01; *** p < .001

RI-CLPM model:

x = Family Environment, y = Problematic Gaming

Regression Paths

		Standardized					
**Predictor**	**DV**	**β**	**95% CI**	**sig**	**SE**	**z**	**p**
lx1	lx2	0.108	0.034 — 0.181	**	0.037	2.876	0.004
ly1	lx2	−0.066	−0.129 — −0.004	*	0.032	−2.070	0.038
lx2	lx3	0.113	0.034 — 0.192	**	0.040	2.816	0.005
ly2	lx3	−0.068	−0.133 — −0.003	*	0.033	−2.056	0.040
lx1	ly2	−0.068	−0.129 — −0.008	*	0.031	−2.207	0.027
ly1	ly2	0.172	0.093 — 0.251	***	0.040	4.274	<0.001
lx2	ly3	−0.072	−0.136 — −0.008	*	0.033	−2.200	0.028
ly2	ly3	0.176	0.094 — 0.259	***	0.042	4.190	<0.001

* p < .05; ** p < .01; *** p < .001

Latent Factor Correlations

**Factor**	**Factor**	**r**	**95% CI**	**sig**	**SE**	**p**
ix	iy	−0.423	−0.520 — −0.325	***	0.050	0.000
lx1	ly1	−0.129	−0.195 — −0.062	***	0.034	<0.001
lx2	ly2	−0.119	−0.196 — −0.042	**	0.039	0.003
lx3	ly3	−0.108	−0.170 — −0.046	***	0.032	<0.001

* p < .05; ** p < .01; *** p < .001

Latent Factor Variance/Residual Variance

**Factor**	**Variance**	**Std. Variance**	**sig**	**p**
lx1	0.609	1.000	***	0.000
lx2	0.663	0.982	***	0.000

lx3	0.663	0.980	***	0.000
ly1	0.629	1.000	***	0.000
ly2	0.638	0.963	***	0.000
ly3	0.638	0.960	***	0.000
ix	0.405	1.000	***	0.000
iy	0.378	1.000	***	0.000

Explained Variance

Variable	R^2
lx2	0.018
lx3	0.020
ly2	0.037
ly3	0.040
