# Supplementary material for: Problematic gaming in occasional, low, intermediate or heavy use gamers – not all screen time is created equal – A reanalysis
Source: PLoS One. 2026 Oct 21;21(10):e0356080. doi: 10.1371/journal.pone.0356080 (PMC13686491; doi:10.1371/journal.pone.0356080)
Supplement: Supplementary file 3 [file pone.0356080.s001.docx]

**Problematic gaming in occasional, low, intermediate or heavy gamers – not all screen time is created equal – a reanalysis**

**Supplementary Information**

**1. Dataset** **and measures included in this reanalysis.**

The list of variables collected in the original study is available in Table 1 of Choo et al. 2010 [1] and the full list of papers published using this dataset is available in section 5 of the supplementary information.

1.1. **Missing data and attrition.**

The proportion of missing data ranged from 0% for video game consumption at baseline (which was used for grouping) to 13% (impulsivity) at baseline and increased with each wave (See Table S1). The proportion of missing data was higher in the occasional-gamers, except for PG and VG hours at baseline (S1 Fig).

**Table S1. Proportion of missing data per measure and wave.**

| **Measure** | **W1** | **W2** | **W3** |
| --- | --- | --- | --- |
| PG | 0.06 | 0.19 | 0.23 |
| VG Hours | 0.00 | 0.22 | 0.26 |
| Impulsiveness | 0.13 | 0.20 | 0.23 |
| Family environment | 0.08 | 0.20 | 0.22 |

Missing data was not equally distributed between groups and was higher in occasional-gamers compared to other groups (S1 Fig). To reduce possible bias due to differential attrition between groups, our main analyses focused on completers, i.e., participants who had data at wave 1 and 3, and no missing-data imputation technique was applied.

**S1 Fig. Proportion of missing data per variable, group and wave. PG = problematic gaming, VG = VG hours, Imp = impulsiveness, Fam = Family environment.**

**Table S2. Number of observations available for the different variables, waves and groups in the raw dataset, and corresponding attrition rates.**

| Predictor | Group | N Wave1 | N Wave3 | N W1 & W3 | Attrition (%) |
| --- | --- | --- | --- | --- | --- |
| Problematic Gaming | Occasional Gamers | 678 | 472 | 472 | 30.38 |
|  | Low Gamers | 739 | 665 | 634 | 10.01 |
|  | Intermediate Gamers | 707 | 615 | 562 | 13.01 |
|  | Heavy Gamers | 715 | 592 | 545 | 17.2 |
| VG Hours | Occasional Gamers | 678 | 444 | 444 | 34.51 |
|  | Low Gamers | 786 | 631 | 631 | 19.72 |
|  | Intermediate Gamers | 785 | 584 | 584 | 25.61 |
|  | Heavy Gamers | 785 | 573 | 573 | 27.01 |
| Impulsiveness | Occasional Gamers | 518 | 475 | 379 | 8.3 |
|  | Low Gamers | 728 | 669 | 628 | 8.1 |
|  | Intermediate Gamers | 707 | 614 | 562 | 13.15 |
|  | Heavy Gamers | 694 | 587 | 525 | 15.42 |
| Family Environment | Occasional Gamers | 560 | 481 | 410 | 14.11 |
|  | Low Gamers | 747 | 669 | 643 | 10.44 |
|  | Intermediate Gamers | 734 | 625 | 591 | 14.85 |
|  | Heavy Gamers | 737 | 591 | 557 | 19.81 |

To probe the plausibility of MNAR, we compared completers (W1 and W3) and non-completers (W1 only) on all baseline variables (Tables S3 continuous, S4 categorical). Compared to completers, non-completers reported higher baseline VG hours, higher impulsiveness, slightly more positive family environment, were older, and had lower parental education; importantly, non-completers reported lower baseline PG, not higher. This pattern is the opposite of the worst-case MNAR scenario (in which drop-out is concentrated among adolescents with worsening PG), and suggests that the attrition pattern observed would tend to under- rather than over-estimate the longitudinal VG-hours→PG and impulsivity→PG associations in the completer subsample, which is consistent with our finding that these are the strongest effects.

**Table S3. Completers vs non-completers - continuous variables.**

| **variable** | **n_completers** | **n_dropouts** | **mean_compl** | **mean_dropout** | **sd_compl** | **sd_dropout** | **mean_diff** | **cohen_d** | **t_p** | **wilcox_p** |
| --- | --- | --- | --- | --- | --- | --- | --- | --- | --- | --- |
| **PG** | 2213 | 626 | 1.87 | 1.72 | 1.87 | 1.85 | 0.15 | 0.08 | 0.082 | 0.036 |
| **VG Hours** | 2213 | 821 | 19.74 | 22.64 | 25.13 | 27.36 | -2.90 | -0.11 | 0.008 | 0.090 |
| **Impuls.** | 2018 | 629 | 2.25 | 2.34 | 0.45 | 0.40 | -0.09 | -0.20 | <0.001 | <0.001 |
| **Fam. Env.** | 2082 | 696 | 3.27 | 3.16 | 0.58 | 0.59 | 0.11 | 0.19 | <0.001 | <0.001 |
| **Age** | 2094 | 719 | 10.95 | 11.97 | 2.00 | 2.04 | -1.02 | -0.51 | <0.001 | <0.001 |
| **Mother Education** | 2058 | 709 | 6.58 | 5.95 | 2.11 | 2.38 | 0.62 | 0.29 | <0.001 | <0.001 |
| **Father Education** | 2026 | 696 | 6.77 | 6.17 | 1.94 | 2.30 | 0.59 | 0.29 | <0.001 | <0.001 |

**Table S4. Completers vs non-completers - categorical variables**

| **variable** | **chi_sq** | **df** | **p_value** | **cramer_v** |
| --- | --- | --- | --- | --- |
| **group** | 33.17 | 3 | <0.001 | 0.10 |
| **sex** | 2.35 | 1 | 0.125 | 0.03 |
| **race** | 4.12 | 4 | 0.390 | 0.04 |
| **education** | 225.52 | 3 | <0.001 | 0.27 |
| **school** | 365.68 | 11 | <0.001 | 0.33 |

As a more direct robustness check, we re-estimated the main change-on-change regressions (changes in PG between W1 and W3 predicted by changes in VG hours, impulsiveness and family environment) after multiple imputation with chained equations (MICE, m = 20). The MICE coefficients are essentially identical to the completers-only estimates (S2 Fig): in occasional and heavy gamers, the directions, signs and orderings of the effects are unchanged, and the magnitudes differ by less than one standard error. The only notable numerical difference concerns the low-gamer group, in which the impact of VG hours and of family environment on PG is slightly larger after imputation (already a sub-dominant effect in the main analysis). We interpret this convergence as evidence that, within the MAR assumption that MICE makes, the completers-only inferences in the main text are not driven by selection on the outcome. Under more pessimistic MNAR assumptions, no analysis can be definitive, but the combined evidence from (ii) — non-completers having lower, not higher, baseline PG — substantially reduces the plausibility of the most damaging MNAR scenarios.

**S2 Fig. Beta-coefficients of a regression analysis predicting changes in PG from W1 to W3 from changes in VG hours, impulsiveness and family environment over the same period (W1 to W3), separately for the completers only (as in the main manuscript) and after multivariate imputation with chained equations (MICE) using m = 20. As can be seen in the figure below, the coefficients are very similar. The only notable difference concerns low gamers, where the impact of VG hours and family environment on PG is was numerically larger after data imputation (see figure below - MI (m=20)).**

1.2. **Correlation between measures**

S3 Fig shows the correlations between all variables at waves 1 and 3, as well as with their change scores (Diff31). As expected, each change score was correlated positively with wave 3 data and negatively with wave 1 data. Video gaming hours and impulsiveness were positively correlated both within and across waves, whereas family environment correlated negatively with both impulsiveness and VG Hours.

**S3 Fig. Left: correlation matrix between our three predictor and one outcome variables at Wave 1 and Wave 3. Right: correlation between changes in VG Hours, changes in PG, changes in impulsiveness and changes in family environment between wave 1 and wave 3.** Names indicate the wave (W1 = wave 1, W3 = wave 3, Diff31 = difference W3-W1) and the variable (PG = Problematic Gaming, VG = VG Hours, Imp = impulsiveness, Fam = family environment).

**2. Data analysis**

All analyses were conducted in R. All variables were z-scored before the analysis to simplify the interpretation of regression coefficients.

**3. Results**

***3.1. Descriptive statistics at Wave 1 and Wave 3.***

Descriptive statistics for gaming time, impulsiveness, family environment and PG at wave 1 and wave 3, including effect sizes of pairwise group comparisons, are provided below.

Occasional-gamers and low-gamers scored lower on PG compared to intermediate gamers, who in turned scored lower than heavy gamers (while occasional-gamers and low-gamers did not differ much in PG (Cohen’s d = 0.06), low-gamers differed more (*d* = 0.69) than did occasional-gamers (*d* = 0.26) from heavy-gamers). On average, PG was relatively low and decreased significantly between W1 (mean = 2.25, SE = 0.03) and W3 (mean = 1.78, SE = 0.04; *t*(5043) = 9.62, Cohen’s *d =* 0.27). The decrease was significant in all groups and was strongest in heavy-gamers (Cohen’s *d* = 0.54) and lowest in occasional-gamers (Cohen’s *d* = 0.09). Importantly, in each group, some individuals increased and other decreased in PG; the analyses in our main text address this inter-individual variability, independently of the overall mean effect reported here.

Occasional-gamers and low-gamers played less VG hours compared to intermediate-gamers who played less than heavy gamers (with pairwise comparisons ranging from Cohen’s *d* = 0.59 to *d* = 2.16), except for occasional-gamers vs. low-gamers, Cohen’s *d* = 0.03). On average, VG hours increased slightly from W1 (mean = 19.83, SE = 0.31) to W3 (mean = 20.93, SE = 0.36; *t*(5258) = -2.32, Cohen’s *d = -*0.07). VG hours increased in all groups (Cohen’s *d* = 1.01, 0.46 and 0.20 for occasional-gamers, low-gamers and intermediate-gamers, respectively) except in heavy gamers where it decreased significantly (Cohen’s *d* = 1.41). Again, in each group, some individuals increased and other decreased in VG hours; and the analyses in our main text address this inter-individual variability, independently of the overall mean effect reported here.

For Impulsiveness, the low-gamers scored lowest, the heavy gamers scored highest and all pairwise comparisons were significant with effect sizes between Cohen’s *d = 0.11 (occasional-gamers vs intermediate-gamers*) and Cohen’s *d* = 0.42 (low-gamers vs. heavy-gamers), except for occasional-gamers vs low-gamers (Cohen’s *d =* 0.08). Mean Impulsiveness was stable across the 2-years interval.

The same pattern was found for family environment. The lowest levels were found in heavy gamers (mean = 3.14, SE = 0.02), followed by occasional-gamers (mean = 3.21, SE = 0.02), intermediate gamers (mean = 3.28, SE = 0.02) and finally low gamers (mean = 3.38, SE = 0.02), with all pairwise comparisons being significant with Cohen’s *d* values between 0.11 and 0.42. On average, family environment was stable across the 2-years interval.

***3.2. SCA results.***

In the main analysis of changes in VG hours, baseline VG hours were included as a control variable, which is redundant with the group analysis given that the groups are based on this same variable. We thus ran an additional SCA analysis assessing the impact of a change in VG hours on changes in PG, without controlling for VG hours at study inception, which replicated the results as can be seen in S4 Fig below.

**S4 Fig. Results of the analysis based on change scores between wave 1 and wave 3 (W1-W3) without controlling for VG hours at W1. Left panel: partial eta-squared which reflect the % of explained variance in PG changes by changes in each predictor. Right panel: standardized beta coefficients showing the direction and strength of the association**. Facets represent different predictors: VG = changes in weekly VG hours.

***3.3. Linear mixed model***

The following model was used to test the effect of changes in impulsiveness and in gaming hours on PG in occasional-gamers.

$$\Delta PG \sim$$

$$\boldsymbol{\Delta}_{\boldsymbol{31}}\boldsymbol{Impuls:Group +}\boldsymbol{\Delta}_{\boldsymbol{31}}\boldsymbol{VG:Group}\boldsymbol{+}$$

$$W_{1}\_PG+W_{1}\_VG+W_{1}\_Impulsiveness+$$

$$school+education+age+maedu+paedu+race+sex$$

∆PG: changes in PG from W1 to W3; ∆Impuls changes in impulsivity from W1 to W3, ∆VG: changes in video gaming hours. W1_PG: PG at wave 1, W1_VG: video gaming hours at wave 1, W1_impusliveness: impulsivity at wave 1, maedu : maternal education, paedu : paternal education.

***3.4. Results for W1-W2 and W2-W3***

To further explore the time course of the causal impact of changes in VG consumption, impulsivity and family environment, we repeated the combined SCA and Granger-causality analysis separately for wave 1 to wave 2 and for wave 2 to wave 3 (S5 Fig). The goal is to predict changes occurring in the first year (wave 1 to 2) and changes in the second year (wave 2 to 3). In each analysis, we use change scores in VG hours, impulsiveness and family environment to predict changes in PG while controlling for their levels at the preceding time point.

As shown in S5 Fig (effect sizes), the impact of changes in VG hours was strongest in occasional gamers the 2^nd^ year (11%). Otherwise, small-to-medium impact was seen for both occasional-gamers (7%) and low-gamers (7%) during the first year. Changes in impulsiveness impacted most intermediate-gamers during the first year (8%) followed by intermediate-gamers (7%) and heavy-gamers (6%) during the second year. Changes in family environment were either small (< 3%) or negligible.

**S5 Fig. Results of the analysis based on change scores between wave 1 and wave 2 (W1-W2) and between wave 2 and wave 3 (W2-W3). Left panel: partial eta-squared which reflect the % of explained variance in PG changes by changes in each predictor. Right panel: standardized beta coefficients showing the direction and strength of the association**. Facets represent different predictors: VG = changes in weekly VG hours, Imp = changes in impulsivity, Fam = changes in family environment.

**3.5. Results of Moderation analysis**

Moderation analyses confirmed the differential impact of VG hours and impulsiveness in occasional and heavy gamers (S6 Fig). The slope (dose-response) of the relationship between changes in PG and changes in VG hours was steeper in occasional-gamers. In contrast, the relationship between changes in PG and changes in impulsiveness was steeper in heavy-gamers. These results are consistent with the main analysis. Interactions were found between VG hours and gaming group (F(3,117.5) = 10.9, p < .001) and impulsiveness and gaming group (F(3,41.3) = 4.0, p < .01). The impact of VG hours on PG was stronger in occasional-gamers, whereas the effect of impulsiveness was stronger in heavy gamers (S6 Fig).

**S6 Fig. Results of the moderation analysis showing an interaction effects between PG and gaming time (top) and between PG and impulsiveness (bottom).**

**3.6. Cross-Lagged Panel results**

Following a reviewer’s request, we ran cross-lagged panel models to assess the directionality of the reciprocal influences while controlling for auto-regressive effects [2]. In addition, we also conducted random-intercept cross-lagged panel models to further separate within-person from between-person effects. These analyses were performed at the whole group level as their goal is to verify the directionality of influences rather than replicate the effects observed in the main analysis. The results are summarized in Table S5 and S7 Fig. CLPM analyses revealed no relation between gaming time, impulsivity, family environment and problematic gaming at the between-subject level. At the within-subject level, RI-CLPM models suggest a unidirectional (positive) effect of problematic gaming on later impulsiveness, as well as bidirectional (negative) links between PG and family environment. Detailed results are available in a separate [supplementary PDF](https://drive.switch.ch/index.php/s/pG64B0JtiSB6fFG)s.

**Table S5. Summary results of CLPM and RI-CLPM models using Problematic Gaming (PG) as the outcome (column Y) and video game consumption (VG), impulsiveness (Imp) and Family environment (Fam) as predictors (column X).** CLPM models showed only auto-regressive effects and no cross-lagged effect at the between-person level. RI-CLPM models, in contrast, revealed both unidirectional *positive* influences (e.g., PG 🡪 Imp) and bidirectional *negative* influences (e.g., Fam 🡨🡪 PG) at the within-person level. Significant cross-lagged effects are shown in bold font on orange background. Auto-regressive effects are shown with blue background.

| **X** | **Path** | **Beta** | **p** | **Sig** | **R2** | **CFI** | **RMSEA** | **CI** | **SRMR** |
| --- | --- | --- | --- | --- | --- | --- | --- | --- | --- |
| **CLPM Models (between-person effects)** | | | | | | | | | |
| VG | lx1 -> lx2 | 0.83 | 0.00 | *** | 0.67 | 1.00 | 0.03 | [0.015, 0.057] | 0.01 |
|  | lx2 -> lx3 | 0.80 | 0.00 | *** | 0.63 |  |  |  |  |
|  | ly1 -> ly2 | 0.78 | 0.00 | *** | 0.58 |  |  |  |  |
|  | ly2 -> ly3 | 0.77 | 0.00 | *** | 0.57 |  |  |  |  |
|  | ly1 -> lx2 | -0.01 | 0.82 |  | 0.67 |  |  |  |  |
|  | ly2 -> lx3 | -0.01 | 0.82 |  | 0.63 |  |  |  |  |
|  | lx1 -> ly2 | -0.03 | 0.59 |  | 0.58 |  |  |  |  |
|  | lx2 -> ly3 | -0.03 | 0.58 |  | 0.57 |  |  |  |  |
| Imp | lx1 -> lx2 | 0.88 | 0.00 | *** | 0.73 | 1.00 | 0.01 | [0, 0.037] | 0.00 |
|  | lx2 -> lx3 | 0.98 | 0.00 | *** | 0.90 |  |  |  |  |
|  | ly1 -> ly2 | 0.79 | 0.00 | *** | 0.61 |  |  |  |  |
|  | ly2 -> ly3 | 0.93 | 0.00 | *** | 0.85 |  |  |  |  |
|  | ly1 -> lx2 | -0.05 | 0.30 |  | 0.73 |  |  |  |  |
|  | ly2 -> lx3 | -0.05 | 0.28 |  | 0.90 |  |  |  |  |
|  | lx1 -> ly2 | -0.01 | 0.82 |  | 0.61 |  |  |  |  |
|  | lx2 -> ly3 | -0.01 | 0.82 |  | 0.85 |  |  |  |  |
| Fam | lx1 -> lx2 | 0.88 | 0.00 | *** | 0.74 | 1.00 | 0.00 | [0, 0.033] | 0.01 |
|  | lx2 -> lx3 | 0.97 | 0.00 | *** | 0.90 |  |  |  |  |
|  | ly1 -> ly2 | 0.78 | 0.00 | *** | 0.63 |  |  |  |  |
|  | ly2 -> ly3 | 0.91 | 0.00 | *** | 0.86 |  |  |  |  |
|  | ly1 -> lx2 | 0.05 | 0.24 |  | 0.74 |  |  |  |  |
|  | ly2 -> lx3 | 0.05 | 0.22 |  | 0.90 |  |  |  |  |
|  | lx1 -> ly2 | -0.04 | 0.23 |  | 0.63 |  |  |  |  |
|  | lx2 -> ly3 | -0.05 | 0.24 |  | 0.86 |  |  |  |  |
| **RI-CLPM Models (within-person effects)** | | | | | | | | | |
| VG | lx1 -> lx2 | -0.01 | 0.92 |  | 0.00 | 0.99 | 0.04 | [0.029, 0.051] | 0.03 |
|  | ly1 -> lx2 | 0.04 | 0.39 |  | 0.00 |  |  |  |  |
|  | lx2 -> lx3 | 0.00 | 0.92 |  | 0.00 |  |  |  |  |
|  | ly2 -> lx3 | 0.04 | 0.39 |  | 0.00 |  |  |  |  |
|  | ly1 -> ly2 | 0.17 | 0.00 | *** | 0.03 |  |  |  |  |
|  | lx1 -> ly2 | -0.06 | 0.10 |  | 0.03 |  |  |  |  |
|  | ly2 -> ly3 | 0.18 | 0.00 | *** | 0.03 |  |  |  |  |
|  | lx2 -> ly3 | -0.05 | 0.10 |  | 0.03 |  |  |  |  |
| Imp | lx1 -> lx2 | 0.09 | 0.01 | ** | 0.02 | 1.00 | 0.02 | [0.007, 0.033] | 0.02 |
|  | **ly1 -> lx2** | **0.10** | **0.00** | ******* | **0.02** |  |  |  |  |
|  | lx2 -> lx3 | 0.10 | 0.01 | * | 0.02 |  |  |  |  |
|  | **ly2 -> lx3** | **0.10** | **0.00** | ****** | **0.02** |  |  |  |  |
|  | ly1 -> ly2 | 0.17 | 0.00 | *** | 0.04 |  |  |  |  |
|  | lx1 -> ly2 | 0.05 | 0.06 |  | 0.04 |  |  |  |  |
|  | ly2 -> ly3 | 0.18 | 0.00 | *** | 0.04 |  |  |  |  |
|  | lx2 -> ly3 | 0.06 | 0.06 |  | 0.04 |  |  |  |  |
| Fam | lx1 -> lx2 | 0.11 | 0.00 | ** | 0.02 | 1.00 | 0.02 | [0, 0.03] | 0.01 |
|  | **ly1 -> lx2** | **-0.07** | **0.04** | ***** | **0.02** |  |  |  |  |
|  | lx2 -> lx3 | 0.11 | 0.01 | ** | 0.02 |  |  |  |  |
|  | **ly2 -> lx3** | **-0.07** | **0.04** | ***** | **0.02** |  |  |  |  |
|  | ly1 -> ly2 | 0.17 | 0.00 | *** | 0.04 |  |  |  |  |
|  | **lx1 -> ly2** | **-0.07** | **0.03** | ***** | **0.04** |  |  |  |  |
|  | ly2 -> ly3 | 0.18 | 0.00 | *** | 0.04 |  |  |  |  |
|  | **lx2 -> ly3** | **-0.07** | **0.03** | ***** | **0.04** |  |  |  |  |

**S7 Fig. Summary of RI-CLPM results showing significant within-person cross-lagged influences. Betas indicate standardized coefficients.**

**4. Problematic Gaming scale.**

**Table S6*.* Items of the DSM-IV and PG scale as compared to description of DSM-V for IGD diagnosis. The questions used in our study cover all 9 of symptoms of the DSM-V criteria for IGD. The only divergence concerns a tenth item on co-dependence based on the DSM-IV / PG which is absent from DSM-V / IGD.**

| **#** | **DSM IV Item** | **IGD description** | **PG item used in this study** |
| --- | --- | --- | --- |
| 1 | Preoccupation | The person is preoccupied with gaming. | In the past year, are you thinking about computer or video games more and more? |
| 2 | Withdrawal | The person experiences withdrawal symptoms when gaming is not possible. | In the past year, have you become restless or irritable when trying to cut down or stop playing computer or video games? |
| 3 | Tolerance | The person needs to spend more time gaming to satisfy their urge. | In the past year, do you need to spend more and more time or money on computer/video games to feel the same amount of excitement? |
| 4 | Loss of control (unsuccessful attempts to reduce gaming) | The person has made unsuccessful attempts to cut down or stop gaming. | In the past year, have you tried to play computer or video games less often or for shorter periods of time? are you successful? |
| 5 | Loss of interest (giving up activities) | The person has lost interest in other hobbies and entertainment as a result of, or because of, gaming. | In the past year, have you ever skipped your studies or co-curricular activities (CCA) to play more computer or video games? |
| 6 | Continuation despite negative consequences | The person has jeopardized or lost a significant relationship, job, educational or career opportunity because of gaming. | In the past year, has your schoolwork suffered because you spent too much time playing computer or video games? |
| 7 | Deception | The person has used deception with family members, therapists, or others about the extent of their gaming. | In the past year, have you ever lied to family or friends about how much you play computer or video games? |
| 8 | Escape | The person has used gaming to escape negative moods or feelings. | In the past year, have you played computer or video games to escape from problems, bad feelings, or stress? |
| 9 | Jeopardizing Relationships or Career | The person has jeopardized or lost a job or relationship due to gaming | In the past year, have you ever stolen a video game from a store or a friend, or stolen money so you could buy or play a video game? |
| 10 | Codependence | NOT IN IGD | In the past year, have you ever needed to borrow money so you could get or play computer or video games? |

**5. Published papers with this dataset (chronological order):**

1. Choo, H. *et al.* Pathological video-gaming among Singaporean youth. *Ann Acad Med Singap* **39**, 822–829 (2010). [10.47102/annals-acadmedsg.V39N11p822](http://dx.doi.org/10.47102/annals-acadmedsg.V39N11p822)
2. Gentile, D. A., Choo, H., Liau, A., Sim, T., Li, D., Fung, D., & Khoo, A. (2011). Pathological video game use among youths: A two-year longitudinal study. *Pediatrics*, *127*(2), e319-329. <https://doi.org/10.1542/peds.2010-1353>
3. Gentile, D. A., Swing, E. L., Lim, C. G., & Khoo, A. (2012). Video game playing, attention problems, and impulsiveness: Evidence of bidirectional causality. *Psychology of Popular Media Culture*, *1*, 62–70. <https://doi.org/10.1037/a0026969>
4. Gentile, D. A., Li, D., Khoo, A., Prot, S., & Anderson, C. A. (2014). Mediators and Moderators of Long-term Effects of Violent Video Games on Aggressive Behavior: Practice, Thinking, and Action. *JAMA Pediatrics*, *168*(5), 450–457. <https://doi.org/10.1001/jamapediatrics.2014.63>
5. Prot, S., Gentile, D. A., Anderson, C. A., Suzuki, K., Swing, E., Lim, K. M., Horiuchi, Y., Jelic, M., Krahé, B., Liuqing, W., Liau, A. K., Khoo, A., Petrescu, P. D., Sakamoto, A., Tajima, S., Toma, R. A., Warburton, W., Zhang, X., & Lam, B. C. P. (2014). Long-Term Relations Among Prosocial-Media Use, Empathy, and Prosocial Behavior. *Psychological Science*, *25*(2), 358–368. <https://doi.org/10.1177/0956797613503854>
6. Busching, R., Gentile, D. A., Krahé, B., Möller, I., Khoo, A., Walsh, D. A., & Anderson, C. A. (2015). Testing the reliability and validity of different measures of violent video game use in the United States, Singapore, and Germany. *Psychology of Popular Media Culture*, *4*(2), 97–111. <https://doi.org/10.1037/ppm0000004>
7. Liau, A. K., Choo, H., Li, D., Gentile, D. A., Sim, T., & Khoo, A. (2015). Pathological video-gaming among youth: A prospective study examining dynamic protective factors. *Addiction Research & Theory*, *23*(4), 301–308. <https://doi.org/10.3109/16066359.2014.987759>
8. Gentile, D. A., Berch, O. N., Choo, H., Khoo, A., & Walsh, D. A. (2017). Bedroom media: One risk factor for development. *Developmental Psychology*, *53*, 2340–2355. <https://doi.org/10.1037/dev0000399>
9. Ferguson, C. J., & Wang, J. C. K. (2019). Aggressive Video Games are Not a Risk Factor for Future Aggression in Youth: A Longitudinal Study. *Journal of Youth and Adolescence*, *48*(8), 1439–1451. <https://doi.org/10.1007/s10964-019-01069-0>
10. Ferguson, C. J., Jeong, E. J., & Wang, J. C. K. (2022). Pathological gaming: A longitudinal study from the perspectives of mental health problems and social stress model. *The Journal of General Psychology*, *0*(0), 1–21. <https://doi.org/10.1080/00221309.2022.2060176>

**References cited in the supplementary information**

1.	Choo H, Gentile DA, Sim T, Li D, Khoo A, Liau AK. Pathological video-gaming among Singaporean youth. Ann Acad Med Singap. 2010 Nov;39(11):822–9.

2.	Hamaker EL, Kuiper RM, Grasman RPPP. A critique of the cross-lagged panel model. Psychological Methods. 2015 Mar;20(1):102–16.
