# Supplementary material for: Problematic gaming in occasional, low, intermediate or heavy use gamers – not all screen time is created equal – A reanalysis
Source: PLoS One. 2026 Oct 21;21(10):e0356080. doi: 10.1371/journal.pone.0356080 (PMC13686491; doi:10.1371/journal.pone.0356080)
Supplement: Supplementary file 4 [file pone.0356080.s012.docx]

Internet Gaming Disorder in novel, low, intermediate or heavy gamers – not all screen time is created equal

Benoit Bediou, Sezen Cekic, Douglas A. Gentile and Daphne Bavelier

1. funding

We added the following statement in the data availability section: *"The funders provided only financial support and had no involvement in the research's study design, data collection, analysis, writing, or decision to publish."*

2. Data availability

In the online submission form you indicate that your data is not available for proprietary reasons and have provided a contact point for accessing this data. Please note that your current contact point is a co-author on this manuscript. **According to our Data Policy, the contact point must not be an author on the manuscript and must be an institutional contact, ideally not an individual.** Please revise your data statement to a non-author institutional point of contact, such as a data access or ethics committee, and send this to us via return email. Please also include contact information for the third party organization, and please include the full citation of where the data can be found.

We have updated the contact information for data availability. The institutional contact for data sharing is the Research Support Office at Nanyang Technological University. Research Support Office (RSO), NTU Singapore [Dir-RSO@ntu.edu.sg](mailto:Dir-RSO@ntu.edu.sg)

3. References

If the reviewer comments include a recommendation to cite specific previously published works, please review and evaluate these publications to determine whether they are relevant and should be cited. There is no requirement to cite these works unless the editor has indicated otherwise.

All references suggested by reviewers have been added where appropriate.

Additional Editor Comments:

Dear authors,

Your submission, "Internet Gaming Disorder in novel, low, intermediate or heavy gamers – not all screen time is created equal" has been reviewed. We encourage you to submit a revised version that carefully addresses all reviewers’ comments. Please note that failure to adequately address the concerns raised may result in the rejection of your submission. An editor will evaluate the revisions and determine whether they meet the necessary criteria for publication.

The reviewers' comments are included at the bottom of this email. Please respond to each point in the reviewers' comments and identify what changes you have made. If you find any of the reviewer's comments to be unjustified or inappropriate, please explain your perspective.

[Note: HTML markup is below. Please do not edit.]

Reviewers' comments:

Reviewer's Responses to Questions

**Comments to the Author**

1. Is the manuscript technically sound, and do the data support the conclusions?

The manuscript must describe a technically sound piece of scientific research with data that supports the conclusions. Experiments must have been conducted rigorously, with appropriate controls, replication, and sample sizes. The conclusions must be drawn appropriately based on the data presented.

Reviewer #1: Partly

Reviewer #2: Yes

2. Has the statistical analysis been performed appropriately and rigorously?

Reviewer #1: Yes

Reviewer #2: Yes

3. Have the authors made all data underlying the findings in their manuscript fully available?

The [PLOS Data policy](http://track.editorialmanager.com/CL0/http:%2F%2Fwww.plosone.org%2Fstatic%2Fpolicies.action%23sharing/1/010f019a90f1e206-151cdea4-ed4f-40b4-a487-0bf8bd0629c6-000000/SyJDicyMxfzKcwfYeHlZlgoYJMyuHorCa91gbNrQw2Q=237) requires authors to make all data underlying the findings described in their manuscript fully available without restriction, with rare exception (please refer to the Data Availability Statement in the manuscript PDF file). The data should be provided as part of the manuscript or its supporting information, or deposited to a public repository. For example, in addition to summary statistics, the data points behind means, medians and variance measures should be available. If there are restrictions on publicly sharing data—e.g. participant privacy or use of data from a third party—those must be specified.

Reviewer #1: Yes

Reviewer #2: No

4. Is the manuscript presented in an intelligible fashion and written in standard English?

PLOS ONE does not copyedit accepted manuscripts, so the language in submitted articles must be clear, correct, and unambiguous. Any typographical or grammatical errors should be corrected at revision, so please note any specific errors here.

Reviewer #1: Yes

Reviewer #2: Yes

4. Review Comments to the Author

   Please use the space provided to explain your answers to the questions above. You may also include additional comments for the author, including concerns about dual publication, research ethics, or publication ethics. (Please upload your review as an attachment if it exceeds 20,000 characters)

Based on the comments from the reviewers, the introduction has been entirely rewritten, and the work now discusses problematic gaming and not IGD, a term coined after our problematic gaming questionnaire was administered (as correctly noted by Reviewer 1). As we detail below, our questionnaire indeed differs by one question as compared to those of IGD. The rewritten introduction is divided into 3 sections. We begin by reviewing the different modifiable risk and protecting factors found in previous research. These are now introduced following an epidemiological model that distinguishes between factors related to the agent (game factors), to the host (intrapersonal factors) and to the environment (social and interpersonal factors). In this context, we introduce the multidimensional model of impulsivity which links impulsivity to other related constructs (e.g., self-control, impulse regulation), as well as to presumably unrelated factors, such as maladaptive cognitions and dysregulated behaviors (e.g., rule-breaking) as proposed in the I-PACE model. The second section focuses on the heterogeneity of the association between gaming time and problematic gaming. In the third and last section, we introduce our study goal and hypotheses which are that (1) increases in VG consumption and impulsiveness will be positively associated with increases in PG symptoms, while improvements in the family environment will be protective; and (2) the predictive strength of these factors will vary significantly depending on the baseline gaming habit group, consistent with the hypothesized non-linear model. This rewriting clarifies the hypotheses tested and methodology of the study.

Reviewer #1: In this paper the authors explore three factors – impulsiveness, gaming time and family environment – as risk and resilience factors for internet Gaming Disorder (IGD) across a two year longitudinal study in children. Gaming time and impulsiveness were found to be risk factors for IGD symptoms whereas a better family environment was protective.

This paper had some interesting findings but had a substantial number of issues as well.

Introduction

Lines 52-57. It isn’t clear what was studied longitudinally. Was it persistence of IGD symptoms? Was it treatment?

The meta-analysis by Zhuang et al. focused on studies with a longitudinal design, whether including a treatment (intervention) or not (i.e. IGD persistence). This has been clarified in the text.

Lines 57-58. Also, it is not true that impulsivity and game time are the two strongest predictors. At r=.11, impulsivity was a weaker predictor than self control (r=-.27) and parent child relationship (r=-.15). If self control and impulsivity have differing effect sizes then they are different constructs and should not be treated interchangeably.

We appreciate the reviewer drawing our attention to the difference between impulsivity and self-control. We have clarified that self‑control (the positive side) refers to the capacity to generate adapted behavioral responses, often tested in the context of questionnaires presenting everyday life situations. Impulsivity (the down side) refers to the personality trait which can be measured either using “objective” task‑based measures or using subjective questionnaires such as the UPPS or the Barratt Impulsiveness Scale, as in the present work. To avoid confusion, we have revised the text to clarify that these constructs are distinct, and we also discuss how these two constructs interact.

In terms of game time, the authors note in lines 84-93 that the correlation between gaming time and IGD is generally small, and this is my experience. This undermines the contention that game time is the strongest predictor of IGD.

As noted by the reviewer, in the meta‑analysis referred above and later in the discussion, the correlation between game time and problematic gaming is generally small. We have rewritten to properly characterize its predictive strength. Our point was rather to draw attention to the fact that game time remains one of the most consistently examined variables in the literature. We have rephrased accordingly.

Overall, the second paragraph could be rewritten for clarity and focus.

The second paragraph of the introduction (and in fact the whole introduction) has been substantially rewritten to increase clarity and focus, as well as to further acknowledge the existence of other modifiable risk and protective factors (see next comments from Reviewer 1).

Paragraph beginning line 94
This paper seems to be about predictors of IGD rather than outcomes, and this paragraph on the impact of IGD on mental health seems to come from nowhere and not be linked to the central tenets of the paper. If this paper is centrally interested in outcomes as well as predictors then the introduction needs to be more clearly framed with the aims of the paper stated clearly up front.

The reviewer is correct. We have removed the section on the relationship between gaming and other health consequences of problematic gaming as this is not directly relevant to the present study.

Lines 136-138. I do not agree that “the most common modifiable risk and protective factors for IGD identified in past research” are “gaming time, impulsiveness, and family environment”. There are many risk and protective factors for IGD (for example, see the Warburton et al 2022 risk and resilience paper on IGD with a large range of factors, Li et al 2023 with risk and protective factors related to GD, Jeong et al 2020 on several factors associated with IGD persistence plus others; there are several papers on risk and protective factors for problematic social media use that may also be relevant). Many such factors are modifiable. I think it is important that all key risk and protective factors be acknowledged in the introduction, with a compelling reason then given as to why the factors of interest in this study were singled out.

We thank the reviewer for pointing out these additional studies. We now provide an extended list of modifiable risk and protective factors in the introduction, including the references suggested by Reviewer 1 as well as new references to other relevant work. We also made explicit that the choice of factors of interest was guided by the epidemiological triad framework to cover factors associated with each level: the agent (gaming consumption), the host (impulsivity) and the environment (family environment).

Lines 145-157. Discusses results rather than hypotheses. I will deal with the age of the data elsewhere.

We have rephrased this section of the introduction to present our hypotheses rather than results.

Overall, the introduction lacked clarity and focus and for me did not provide a compelling argument for the methodology of the study conducted.

We thank Reviewer 1 for their constructive feedback. We have made substantial changes to improve the clarity and focus of the introduction as well as to better introduce our hypotheses and how they justify the methodology.

Method

The single biggest issue with this paper is the age of the data (2007-2009), all of which predates by several years the proposed diagnostic criteria for IGD (2013). Apart from the issue of publishing data now nearly 20 years old, how can a person meaningfully study data purported to measure a phenomenon that was not created, reported or measured until several years afterward? At the very least this study must be reframed as being about problematic gaming rather than as being related to a disorder not conceptualised until after the data were collected.

We acknowledge this anachronism and have now rewritten the manuscript to refer to problematic gaming (PG) rather than IGD. The questions use in the present study cover 9 of the 10 symptoms of the DSM-V criteria for IGD with the one divergence concerning the ***continuation*** question in the DSM-V which is absent and replaced by a question about negative consequences (***sneaky behaviors)*** in the present study. This has been clarified by adding a column with the IGD items to Table S4 and updating the legend to mention the diverging item.

It is not sufficient to say that the measures and methodology are reported elsewhere. This paper must stand alone in terms of describing the sample and measures used here. Given the contentiousness of claiming IGD was measured (see below) it is crucial that all measures be made clear and described in sufficient detail. It is also important that key sample characteristics are reported.

We have moved the details about the sample and the measures from the supplementary information to the methods section of the main manuscript.

It is also purported that the study measured IGD. This cannot be factually true given that no such diagnosis existed at the time of the study.

This important point is now clarified in the introduction, where we note that we use the term problematic gaming in this work to cover PG, GD and IGD, as the DSM scales have changed.

Results

Should decimals be to two decimal points (e.g., Table 2)?

We have added the second decimals that were missing.

I think it is important to note that the IGD score is [presumably – we never see the measure] a score of IGD symptomology providing a continuous numeric measure on a continuum, rather than an indication of actually having a gaming disorder. So this is not really a study of IGD or any sort of disorder in a way, it is a study of the level of symptoms, with low levels at one end and higher levels at the other, with no way of knowing which, if any, participants had a gaming disorder. In line 335 diagnosis of IGD is discussed in relation to the data. Again, these data predate the diagnosis or any measure of IGD and the data are continuous without categorising IGD.

We have clarified that the dependent variable is the sum of all 10 items, which are available in Table S2, and represents a measure of the severity of PG symptomatology.

Paragraph starting line 248. It is not simply the case that novel gamers had some IGD symptoms at wave 1 – their levels were nearly as high as at wave 3 and nearly as high as all other groups. Indeed higher than the ‘low’ group. This does not seem to be entirely an artefact of the wording or timing of presentation of measures – for me it is a significant anomaly that casts doubt on the measure of IGD and all the IGD data.

We thank the reviewer for raising this important point. It should not come as a surprise that, amongst novel gamers, there are children who are not allowed to play video game at home and yet may engage in video game play outside of the family setting. This disconnect is not surprising and in fact aligned with the most frequently reported “symptoms” at baseline in novel gamers being, from most frequent to least frequent: items 9 (loss of control / unsuccessful attempts), followed by item 6 (escape), item 2 (withdrawal), item 7 (loss of interests), and item 1 (preoccupation).

This has now been addressed lines 360-372 of the revised MS.

Overall I found the results hard to follow and interpret, but having said that do think the findings are interesting and publishable.

We thank the reviewer and have rephrased the results and discussion to make them easier to follow.

Discussion

Line 365. Is ‘remarkable’ the best word for a finding that is strongly in line with past research? Maybe noteworthy ….

We have replaced remarkable with noteworthy

I am not sure one can categorise IGD as an impulse control problem (line 383). Rather, impulsiveness is one risk factor. It is certainly not an adequate description of the disorder.

We thank the reviewer for this clarification. We have removed this sentence and rephrased part of our text. We agree with the reviewer that it does not provide direct evidence for considering PG as an impulse control problem.

Overall I found the discussion to be lacking in depth, entirely omitting the clear limitations of the paper or adequately situating findings in existing literature.

The discussion now better situates our findings within the existing literature. We have also added a limitation section. In particular, we now emphasize that the direction and magnitude of the observed effects is consistent with the prior work discussed in the introduction. We also acknowledge the inherent limitations of our measures of family environment and of impulsivity, especially when it comes to capturing more complex dynamics associated with the constructs of social environment and of impulse regulation.

Minor issues

There is often not a gap between a word and a following reference number in brackets.

Missing spaces have been added.

In Summary

This paper has a number of serious issues – the age of the data, the measure of IGD predating the existence of the diagnosis, the lack of detail about key parts of the study, and a lack of clarity and focus across the paper. I do think the results are interesting but would need to be presented in a heavily redrafted paper with a completely new focus (not IGD).

We thank the reviewer for their thoughtful and constructive feedback on our manuscript. We hope our interesting results are now better contextualized thanks to the novel introduction, the clarification about the collected measures, the amount of missing data and attrition rates across waves and measures, as well as the updated discussion. We thank the reviewer for their suggestions as to how to strengthen our paper.

Reviewer #2: Major Issues Requiring Revision:
1. Data Availability (Critical)

Does not meet PLOS ONE standards
Must provide deidentified data to controlled-access repository or substantially enhance supplementary materials with complete analysis code and results

We have updated the contact information for data availability. The institutional contact for data sharing is the Research Support Office at Nanyang Technological University. Research Support Office (RSO), NTU Singapore [Dir-RSO@ntu.edu.sg](mailto:Dir-RSO@ntu.edu.sg)

2. Analytical Approach

Recommendation: Replace or supplement difference score analyses with:

Cross-lagged panel models to establish temporal precedence.
Growth curve models to capture trajectories
These approaches handle missing data better and test directional hypotheses more rigorously

We thank the reviewer for these thoughtful suggestions. Our use of Specification Curve Analysis (SCA) of change scores (including autoregressive effects) was specifically chosen to address potential p-hacking and model uncertainty inherent in longitudinal observational data analysis, particularly concerning the selection of covariates. While CLPMs and RI-CLPMs are excellent for isolating within-person dynamics, the SCA framework offers superior robustness against specification choices.

Yet, because the SCA doesn’t assess the directionality of the associations, we have now complemented the main (SCA) analysis with cross-lagged panel models (CLPM) which provide better tests of directionality of reciprocal influences while controlling for auto-regressive effects. In addition, we also conducted random-intercept cross-lagged panel models (RI-CLPM) which better separate within-person from between-person effects. These analyses were performed at the whole group level as their goal is to verify the directionality of influences rather than replicate the effects observed in the main analysis. The results are summarized in supplementary Table S3 and supplementary Figure S6.

CLPM analyses revealed no significant influences at the between-person level. Yet, at the within-subject level, RI-CLPM models suggested a unidirectional (positive) influence of PG on impulsiveness (more PG symptoms at time t leading to higher levels of impulsiveness at time t + 1) and bidirectional (negative) influences between family environment and PG. Of note, no relationship was found between gaming time and PG whether at the between-subject or at the within-subject level. This new analysis is now noted in the Introduction, lines 286-295. The results are then summarized in the results section, lines 484-498.

In addition, we now also discuss these new results lines 561-576 of the revised MS and highlight the strength of our multi-method approach as requested by Reviewer 2.

Current claim (lines 269-271): "we can not only determine the direction of influence" overstates what difference scores can establish

This section has been reworded.

3. Missing Data & Attrition

Required: Add section describing:
Pattern of missingness across waves.
Comparison of completers vs. non-completers on key variables
Sensitivity analyses (if feasible)
Concern: Differential attrition by gaming group could bias results

We have added a section on missing data. The proportion of missing data ranged from 0% for video game consumption at baseline (which was used for grouping) to 13% (impulsivity) at baseline and increased with each wave (See Table S1).

Missing data was not equally distributed between groups and was higher in novel-gamers compared to other groups, except for PG and VG hours at baseline (Figure S1). To reduce possible bias due to differential attrition between groups, our main analyses focused on completers, i.e., participants who had data at wave 1 and 3, and no missing-data imputation technique was applied. This better controls for potential bias related to differential attrition between groups.

This has been clarified lines 256-262 of the revised MS and in the supplementary information, Table S1 and Figure S1.

4. Grouping Strategy

Consider: Justify quantile-based grouping or explore:
Theory-driven cutoffs (e.g., APA's 30 hours/week mentioned line 245)
Data-driven approaches (latent profile analysis)
Sensitivity analyses with different grouping schemes

We used a data-driven approach to categorize participants in quartiles according to their video game consumption at study inception. This choice ensures an equal number of participants in each group. In line with our hypothesis of non-linear associations between gaming time and problematic gaming, at least 3 groups were needed. We note that, among the four groups identified, we are well aligned with the theory-driven APA cutoff of 30 hours/week defining the heavy-gamers group. A smaller number of groups (i.e., tertiles) would result in lower cutoff, whereas a higher number (five or more) would result in smaller groups and thus lower power.

5. Novel-Gamers Definition
Lines 249-259 reveal measurement timing issues
Required clarification: Were questions about "usual" gaming and "past year" IGD symptoms administered in the same session or different sessions?
This affects interpretation of results for novel-gamers substantially.

All surveys were administered in the same session. At each wave, participants were asked about their usual gaming consumption and their problematic gaming symptoms during the past year.

Moderate Issues:

6. Effect Size Interpretation

While the stratified findings are interesting, the practical significance needs discussion
Even in novel-gamers, VG hours explain only 12% of IGD variance
Add: Discussion of what these effect sizes mean for intervention design

We thank the reviewer for raising this important point. We have now added the following text to the discussion lines 592-608 which provides a benchmark for interpretation of effect-sizes magnitudes as well as a comparison with effect-sizes from CBT intervention studies to situate their practical significance.

7. Exploratory Analyses (Figure 3)

Apply FDR correction for multiple comparisons
Be more cautious about interpreting "sneaky" and "deception" symptoms (lines 342-351)
These are interesting hypotheses but need replication

For large sample sizes, we followed the general recommendation to rely on effect sizes rather than significance levels. The results of analysis by symptom (i.e., item of the problematic gaming scale) are indeed presented as exploratory and in need of replication. We have further clarified the limitations in the discussion lines 561-575.

8. Temporal Ordering

Strengthen claims: The phrase "changes in VG consumption influenced changes in IGD" (lines 26-27) implies causation that difference scores cannot establish
Suggest rewording throughout to reflect associations rather than causal effects

We have rephrased in terms of association rather than influence.

9. Alternative Explanations

The discussion could address:

Why does impulsivity matter more for heavy gamers? (addressed briefly)
Could reverse causation explain some findings?
Role of game genres, social gaming, etc.

We have amended the discussion to more directly address these points (lines 516-545).

Minor Issues:

10. Methods Detail

SI section mentions IGD, VG consumption, impulsivity measures but main text should briefly describe:

How many items in each scale?
Response formats?
Reliability estimates?

The number of items and response formats are now available in the methods section. We have added the Cronbach’s alpha for each measure (0.70 for PG, 0.69 for impulsiveness and 0.78 for family environment) in the relevant section of the methods.

11. Table 1

Add column showing attrition rates by group
Because attrition varies across groups and measures, we have added a new supplementary Table S2 which shows the number of observations available for each variable and group at wave 1 and wave 3, with corresponding attrition rates.

Consider adding baseline comparisons of groups on outcome measures

Baseline differences are reported in supplementary results (section 3.1).

12. Figure 1

Very informative but consider adding:
Sample sizes at each timepoint
Statistical tests of mean changes

Sample sizes at each time point are now provided in table S2 for each group and measure. In addition, we have added the total sample size at each wave and measure to the legend of Figure 1.

Tests of mean changes between wave 1 and wave 3 are available in supplementary results, section 3.1.

13. Supplementary Materials

Consider moving Table S1 to main text - these are primary results.
Provide complete correlation matrices for all variables.

Table S1 (now S3) contains the coefficients plotted in Figure 2 and thus could be viewed as redundant. For now, given the length of the paper, we have kept that Table in the supplementary information but would be happy to move it to the main paper if deemed appropriate. Such information is indeed important for future systematic reviews and meta-analyses.

As asked, we have added a new Figure S2 in Sup Mat showing the full correlation matrix.

Strengths to Highlight:

Novel stratification approach addresses real heterogeneity in the literature
Large, longitudinal sample
Sophisticated control for confounds via SCA
Practical implications for tailored interventions
Transparent acknowledgment of exploratory analyses

We thank the reviewer for their suggestion of highlighting the strength of this work. It is now done lines 561-576 of the revised MS.

Suggested Additional Analyses:

Moderation analyses: Formally test whether gaming group moderates VG hours→IGD and impulsivity→IGD relationships
Dose-response: Within each group, is there a linear relationship between change in VG hours and change in IGD?
Bidirectional effects: Even with difference scores, test whether baseline IGD predicts change in VG hours

We have added a moderation analysis, which shows that the slope (dose-response) of the relationship between changes in problematic gaming and changes in **gaming hours** is steeper in **novel-gamers**. In contrast, the relationship between changes in problematic gaming and changes in **impulsivity** is steeper in **heavy-gamers**. These results, which are summarized in Figure S6 and in the main in the main text (lines 478-483), are consistent with the main analysis, confirming the moderating influence of gaming group on the relationship between VG hours and PG and between Impulsivity and PG.

To more formally test the directionality of the effects, we also added CLPM and RICLPM analyses (also requested by reviewer 1).

Discussion Enhancements:

Compare effect sizes to other longitudinal IGD studies
Discuss clinical vs. statistical significance more explicitly
Address limitations more comprehensively (single cultural context, self-report measures, attrition)

We have added a dedicated section to address these points in the discussion lines 592-608.

We also added a section to acknowledge the limitations of the study, lines 577-591.

Clarify intervention implications: How would one "introduce gaming progressively" (line 31)?

We have added examples in the abstract (last sentence).

6. PLOS authors have the option to publish the peer review history of their article ([what does this mean?](https://track.editorialmanager.com/CL0/https:%2F%2Fjournals.plos.org%2Fplosone%2Fs%2Feditorial-and-peer-review-process%23loc-peer-review-history/1/010f019a90f1e206-151cdea4-ed4f-40b4-a487-0bf8bd0629c6-000000/A2C6JnBIHgzvlKdF7L_HsYJiVvhQheVDv-Or728OeRI=237)). If published, this will include your full peer review and any attached files.

If you choose “no”, your identity will remain anonymous but your review may still be made public.

**Do you want your identity to be public for this peer review?** For information about this choice, including consent withdrawal, please see our [Privacy Policy](https://track.editorialmanager.com/CL0/https:%2F%2Fwww.plos.org%2Fprivacy-policy/1/010f019a90f1e206-151cdea4-ed4f-40b4-a487-0bf8bd0629c6-000000/HzaemdBQhgf1X10JDqTAElvTmvGyae9pXyMyvvxFQo8=237).

Reviewer #1: No

Reviewer #2: No

**REFERENCES (cited in the response)**

Ji, Y., Yin, M. X. C., Zhang, A. Y., & Wong, D. F. K. (2022). Risk and protective factors of Internet gaming disorder among Chinese people: A meta-analysis. *The Australian and New Zealand Journal of Psychiatry*, *56*(4), 332–346. https://doi.org/10.1177/00048674211025703

Sallis, J. F., Prochaska, J. J., & Taylor, W. C. (2000). A review of correlates of physical activity of children and adolescents. *Medicine and Science in Sports and Exercise*, *32*(5), 963–975. https://doi.org/10.1097/00005768-200005000-00014

Twenge, J. M., Martin, G. N., & Campbell, W. K. (2018). Decreases in psychological well-being among American adolescents after 2012 and links to screen time during the rise of smartphone technology. *Emotion*, *18*(6), 765–780. https://doi.org/10.1037/emo0000403

Warburton, W. A., Parkes, S., & Sweller, N. (2022). Internet Gaming Disorder: Evidence for a Risk and Resilience Approach. *International Journal of Environmental Research and Public Health*, *19*(9), Article 9. https://doi.org/10.3390/ijerph19095587

Zhuang, X., Zhang, Y., Tang, X., Ng, T. K., Lin, J., & Yang, X. (2023). Longitudinal modifiable risk and protective factors of internet gaming disorder: A systematic review and meta-analysis. *Journal of Behavioral Addictions*, *12*(2), 375–392. https://doi.org/10.1556/2006.2023.00017
