# Supplementary material for: Problematic gaming in occasional, low, intermediate or heavy use gamers – not all screen time is created equal – A reanalysis
Source: PLoS One. 2026 Oct 21;21(10):e0356080. doi: 10.1371/journal.pone.0356080 (PMC13686491; doi:10.1371/journal.pone.0356080)
Supplement: Supplementary file 5 [file pone.0356080.s013.docx]

PONE-D-25-39575R1

Problematic gaming in novel, low, intermediate or heavy gamers – not all screen time is created equal - a reanalysis

PLOS One

Dear Dr. Bediou,

Thank you for submitting your manuscript to PLOS ONE. After careful consideration, we feel that it has merit but does not fully meet PLOS ONE’s publication criteria as it currently stands. Therefore, we invite you to submit a revised version of the manuscript that addresses the points raised during the review process.

Please see the reviewers' comments for the concerns raised and address their comments in a point-by-point fashion. Of note, one of the original reviewers was not available for reviewing again so an independent third reviewer was engaged in order to progress with the PLoS One review procedure.

Please submit your revised manuscript by May 28 2026 11:59PM. If you will need more time than this to complete your revisions, please reply to this message or contact the journal office at plosone@plos.org. When you're ready to submit your revision, log on to <https://www.editorialmanager.com/pone/> and select the 'Submissions Needing Revision' folder to locate your manuscript file.

Please include the following items when submitting your revised manuscript:

- A letter that responds to each point raised by the academic editor and reviewer(s). You should upload this letter as a separate file labeled 'Response to Reviewers'.
- A marked-up copy of your manuscript that highlights changes made to the original version. You should upload this as a separate file labeled 'Revised Manuscript with Track Changes'.
- An unmarked version of your revised paper without tracked changes. You should upload this as a separate file labeled 'Manuscript'.

If you would like to make changes to your financial disclosure, please include your updated statement in your cover letter. Guidelines for resubmitting your figure files are available below the reviewer comments at the end of this letter.

If applicable, we recommend that you deposit your laboratory protocols in protocols.io to enhance the reproducibility of your results. Protocols.io assigns your protocol its own identifier (DOI) so that it can be cited independently in the future. For instructions see: [https://journals.plos.org/plosone/s/submission-guidelines#loc-laboratory-protocols](https://track.editorialmanager.com/CL0/https:%2F%2Fjournals.plos.org%2Fplosone%2Fs%2Fsubmission-guidelines%23loc-laboratory-protocols/1/010f019d89888771-c8b5baf5-f1c4-40a5-b15f-5a4f5c9ea94e-000000/4wQEonaFDsG4h0jkaE9PF5MAQL7w2HHYBel9tZ2ODjY=258). Additionally, PLOS ONE offers an option for publishing peer-reviewed Lab Protocol articles, which describe protocols hosted on protocols.io. Read more information on sharing protocols at [https://plos.org/protocols?utm_medium=editorial-email&utm_source=authorletters&utm_campaign=protocols](https://track.editorialmanager.com/CL0/https:%2F%2Fplos.org%2Fprotocols%3Futm_medium=editorial-email%26utm_source=authorletters%26utm_campaign=protocols/1/010f019d89888771-c8b5baf5-f1c4-40a5-b15f-5a4f5c9ea94e-000000/2kjg3ZXNJq2YNyZVQglFw6in2m2XOA17z13QimQDNjY=258).

As the corresponding author, your ORCID iD is verified in the submission system and will appear in the published article. PLOS supports the use of ORCID, and we encourage all coauthors to register for an ORCID iD and use it as well. Please encourage your coauthors to verify their ORCID iD within the submission system before final acceptance, as unverified ORCID iDs will not appear in the published article. *Only* the individual author can complete the verification step; PLOS staff *cannot* verify ORCID iDs on behalf of authors.

We look forward to receiving your revised manuscript.

Kind regards,

Marc N Potenza
Academic Editor
PLOS One

**Journal Requirements:**

If the reviewer comments include a recommendation to cite specific previously published works, please review and evaluate these publications to determine whether they are relevant and should be cited. There is no requirement to cite these works unless the editor has indicated otherwise.

[Note: HTML markup is below. Please do not edit.]

Dear Pr. Potenza,

Thank you for the opportunity to submit a second revised version of our manuscript. We are grateful to Reviewer 1 for their continued constructive feedback and to Reviewer 3 (Dr. Matthew W. R. Stevens) for the substantial and careful review provided in this round. Together, their comments have helped us sharpen the framing, transparency, and interpretation of our analyses. A detailed point-by-point response accompanies this letter.

In brief, the revised manuscript:

(i) reframes the work as a reanalysis of a longitudinal dataset collected in Singapore (2007–2009), with the title and abstract now stating this explicitly, and the Introduction situating Problematic Gaming (PG) relative to the later DSM-V IGD (2013) and ICD-11 GD (2019) criteria, following Reviewer 1’s recommendation;

(ii) clarifies the construct measured — a 10-item DSM-IV-derived scale of PG symptom severity, mapped item-by-item to the DSM-V IGD criteria in a new Table S6, with corrected labelling. The item previously labelled “sneaky” is now properly referred to as “jeopardize / lying” following pervious terminology in the field. This table aslo makes clear that the one DSM-IV item not retained in DSM-V IGD is co-dependence;

(iii) strengthens the psychometric reporting (adding McDonald’s ω alongside Cronbach’s α for all scales) and benchmarks our values against the published Problematic Gaming/IGD-instrument in the literature;

(iv) renames the baseline - non-regular video game player - group from “novel” to “occasional” gamers, and clarifies the interpretation of their wave-1 PG scores;

(v) acknowledges the limitations of self-reported gaming hours (King et al., 2020; Stevens et al., 2025) and aligns effect-size terminology with that of longitudinal-cross-lagged benchmarks (Orth et al., 2024);

(vi) adds a new paragraph to the Discussion on the heterogeneity of the heavy-gamer group, distinguishing competitive/elite engagement from disordered involvement (Choi et al., 2021; Billieux and colleagues), and explicitly notes that this heterogeneity is expected to attenuate the observed VG-hours→PG association in heavy gamers;

(vii) expands the Discussion / Limitations on interpersonal factors, acknowledging the substantial peer-, school- and online-social literature (with East Asian cohort references), and reframing our family-environment result as a lower bound on the contribution of the interpersonal layer;

(viii) adds a comparison of completers and non-completers (Tables S5–S6) and a multiple-imputation (MICE, m = 20) sensitivity analysis, both supporting the robustness of the main inferences; and

(ix) adds the Moshel et al. (2024) meta-analysis on the relation between gaming-disorder symptoms and inhibitory-control deficits, and addresses minor reporting issues (p-values to three decimals, Field 2013 reference corrected, spacing fixes).

We believe these revisions substantively strengthen the manuscript while preserving its central contribution — that, in a longitudinal adolescent cohort, the relative importance of gaming time, impulsivity and family environment for changes in PG depends systematically on baseline gaming habits — and we hope the revised version will now be suitable for publication in PLOS ONE.

Thank you again for the careful handling of our manuscript.

Sincerely,

Benoit Bediou, on behalf of all co-authors

Reviewers' comments:

Reviewer's Responses to Questions

**Comments to the Author**

1. If the authors have adequately addressed your comments raised in a previous round of review and you feel that this manuscript is now acceptable for publication, you may indicate that here to bypass the “Comments to the Author” section, enter your conflict of interest statement in the “Confidential to Editor” section, and submit your "Accept" recommendation.

Reviewer #1: (No Response)

Reviewer #3: (No Response)

2. Is the manuscript technically sound, and do the data support the conclusions?

The manuscript must describe a technically sound piece of scientific research with data that supports the conclusions. Experiments must have been conducted rigorously, with appropriate controls, replication, and sample sizes. The conclusions must be drawn appropriately based on the data presented.

Reviewer #1: Partly

Reviewer #3: No

3. Has the statistical analysis been performed appropriately and rigorously?

Reviewer #1: Yes

Reviewer #3: No

4. Have the authors made all data underlying the findings in their manuscript fully available?

The [PLOS Data policy](http://track.editorialmanager.com/CL0/http:%2F%2Fwww.plosone.org%2Fstatic%2Fpolicies.action%23sharing/1/010f019d89888771-c8b5baf5-f1c4-40a5-b15f-5a4f5c9ea94e-000000/Cf5xxzcUP4ECGMAlVoc-ePz2ndKnBCVSvUwi1YpFKOM=258) requires authors to make all data underlying the findings described in their manuscript fully available without restriction, with rare exception (please refer to the Data Availability Statement in the manuscript PDF file). The data should be provided as part of the manuscript or its supporting information, or deposited to a public repository. For example, in addition to summary statistics, the data points behind means, medians and variance measures should be available. If there are restrictions on publicly sharing data—e.g. participant privacy or use of data from a third party—those must be specified.

Reviewer #1: Yes

Reviewer #3: Yes

5. Is the manuscript presented in an intelligible fashion and written in standard English?

PLOS ONE does not copyedit accepted manuscripts, so the language in submitted articles must be clear, correct, and unambiguous. Any typographical or grammatical errors should be corrected at revision, so please note any specific errors here.

Reviewer #1: Yes

Reviewer #3: Yes

6. Review Comments to the Author

Please use the space provided to explain your answers to the questions above. You may also include additional comments for the author, including concerns about dual publication, research ethics, or publication ethics. (Please upload your review as an attachment if it exceeds 20,000 characters)

Reviewer #1:

1. Issue: Lines 52-57. It isn’t clear what was studied longitudinally. Was it persistence of IGD symptoms? Was it treatment?

Response: The meta-analysis by Zhuang et al. focused on studies with a longitudinal design, whether including a treatment (intervention) or not (i.e. IGD persistence). This has been clarified in the text.

Adequacy of author response: Not adequately resolved. Longitudinal effects of what on what? This isn’t clear. Is it effects of problematic levels of gaming on mental health-related outcomes? Are the modifiable factors aspects of problematic gaming that can be modified through intervention? I don’t think this reworded section is sufficiently clear.

2. Issue: Lines 57-58. Also, it is not true that impulsivity and game time are the two strongest predictors.

At r=.11, impulsivity was a weaker predictor than self control (r=-.27) and parent child relationship (r=-.15). If self control and impulsivity have differing effect sizes then they are different constructs and should not be treated interchangeably.

In terms of game time, the authors note in lines 84-93 that the correlation between gaming time and IGD is generally small, and this is my experience. This undermines the contention that game time is the strongest predictor of IGD.

Overall, the second paragraph could be rewritten for clarity and focus.

Response: We appreciate the reviewer drawing our attention to the difference between impulsivity and self-control. We have clarified that self control (the positive side) refers to the capacity to generate adapted behavioral responses, often tested in the context of questionnaires presenting everyday life situations. Impulsivity (the down side) refers to the personality trait which can be measured either using “objective” task based measures or using subjective questionnaires such as the UPPS or the Barratt Impulsiveness Scale, as in the present work. To avoid confusion, we have revised the text to clarify that these constructs are distinct, and we also discuss how these two constructs interact.

As noted by the reviewer, the meta analysis referred above and in later in the discussion (lines 84–93), the correlation between game time and problematic gaming is generally small. We have rewritten to properly characterize its predictive strength. Our point was rather to draw attention to the fact that game time remains one of the most consistently examined variables in the literature. We have rephrased accordingly.

The second paragraph of the introduction (and in fact the whole introduction) has been substantially rewritten to increase clarity and focus, as well as to further acknowledge the existence of other modifiable risk and protective factors (see next comments from Reviewer 1).

**Adequacy of author response: Resolved. The authors have restructured this section for clarity and it makes more conceptual sense.**

3. Issue: Paragraph beginning line 94
This paper seems to be about predictors of IGD rather than outcomes, and this paragraph on the impact of IGD on mental health seems to come from nowhere and not be linked to the central tenets of the paper. If this paper is centrally interested in outcomes as well as predictors then the introduction needs to be more clearly framed with the aims of the paper stated clearly up front.

Response: The reviewer is correct. We have removed the section on the relationship between gaming and other health consequences of problematic gaming as this is not directly relevant to the present study.

**Adequacy of author response: Resolved.**

4. Issue: Lines 136-138. I do not agree that “the most common modifiable risk and protective factors for IGD identified in past research” are “gaming time, impulsiveness, and family environment”. There are many risk and protective factors for IGD (for example, see the Warburton et al 2022 risk and resilience paper on IGD with a large range of factors, Li et al 2023 with risk and protective factors related to GD, Jeong et al 2020 on several factors associated with IGD persistence plus others; there are several papers on risk and protective factors for problematic social media use that may also be relevant). Many such factors are modifiable. I think it is important that all key risk and protective factors be acknowledged in the introduction, with a compelling reason then given as to why the factors of interest in this study were singled out.

Response: We thank the reviewer for pointing out these additional studies. We now provide an extended list of modifiable risk and protective factors in the introduction, including the references suggested by Reviewer 1 as well as new references to other relevant work. We also made explicit that the choice of factors of interest was guided by the epidemiological triad framework to cover factors associated with each level: the agent (gaming consumption), the host (impulsivity) and the environment (family environment).

**Adequacy of author response: Resolved. The authors have made substantial rewrites around the risk and protective factors, and made a clearer rationale for the study.**

5. Issue: Lines 145-157. Discusses results rather than hypotheses. I will deal with the age of the data elsewhere.

Response: We have rephrased this section of the introduction to present our hypotheses rather than results.

**Adequacy of author response: Resolved, again through substantive rewriting/**

6. Issue: Overall, the introduction lacked clarity and focus and for me did not provide a compelling argument for the methodology of the study conducted.

Response: We thank Reviewer 1 for their constructive feedback. We have made substantial changes to improve the clarity and focus of the introduction as well as to better introduce our hypotheses and how they justify the methodology.

Adequacy of author response: Resolved (although note there are still some specific issues to address).

Method

7. Issue: The single biggest issue with this paper is the age of the data (2007-2009), all of which predates by several years the proposed diagnostic criteria for IGD (2013). Apart from the issue of publishing data now nearly 20 years old, how can a person meaningfully study data purported to measure a phenomenon that was not created, reported or measured until several years afterward? At the very least this study must be reframed as being about problematic gaming rather than as being related to a disorder not conceptualised until after the data were collected.

Response: We acknowledge this anachronism and have now rewritten the manuscript to refer to problematic gaming (PG) rather than IGD. The questions use in the present study cover 9 of the 10 symptoms of the DSM-V criteria for IGD with the one divergence concerning the continuation question in the DSM-V which is absent and replaced by a question about negative consequences (sneaky behaviors) in the present study. This has been clarified by adding a column with the IGD items to Table S4 and updating the legend to mention the diverging item.

**Adequacy of author response: Not resolved a**nd, for me, must be resolved for the paper to be publishable. The paper starts with the following: “Since it was first introduced in the research appendix of the DSM-V [1] as a condition for further study, Problematic Gaming (PG) has garnered increasing attention in adolescent mental health due to its potential impact on psychological well-being and social functioning [2]. We refer here to PG as signaling the kind of problematic behavior, that is now also known as ‘Internet Gaming Disorder’ (following the DSM /APA terminology) or ‘Gaming Disorder’ according to the 11th Revision of the International Classification of Diseases (ICD-11) [3,4]”.

I have numerous issues with this.
7.1. Problematic gaming (PG) was not introduced in the research appendix of the DSM-V as stated here. Rather, Internet Gaming Disorder (IGD), not PG, was introduced into the ‘Conditions for further study’ section in 2013. IGD was the first suggested clinical diagnosis. However interest in PG significantly predates this inclusion, and much of the research referred to in this paper also predates 2013.
7.2. Problematic Gaming is not a clinical diagnosis – it refers to gaming that is problematic, at both clinical and sub-clinical levels. Thus it would include people with: a. IGD and GD: b. Hazardous Gaming from ICD-11; c. Gaming at problematic but sub-clinical levels, as indicated by elevated scores on measures of problematic gaming, and scores that are elevated but sub-clinical on measures for IGD and GD.
7.3. Because problematic gaming encompasses the above, PG is not synonymous with IGD and GD as suggested above, but is more synonymous with Hazardous Gaming.
In my view this introduction should say something more like, “Although clinical diagnoses for disordered gaming are only fairly recent, with Internet Gaming Disorder (IGD) appearing as a condition for further study in the DSM-V (APA, 2013), and Gaming Disorder as a diagnosis in the ICD-11 (WHO, 2022), problematic gaming (PG) has been identified and studied for considerably longer. PG is characterised by ….. “ going on to describe what it encapsulates, noting it can be measured as hazardous gaming symptoms (ICD-11), elevated but sub-clinical scores on measures for IGD, and elevated scores on other measures of problematic gaming, many of which include criteria that have been published as IGD criteria. There needs to be complete clarity about what PG is, to set up this paper. Crucially, the rest of the paper then needs to align with this definition of PG.

**Response:** We thank the reviewer for forcing us to make these important clarifications. We have now clarified that our use of the PG scale was intended to capture self-reported severity of symptoms around gaming, and was indeed not meant for clinical diagnosis. We have also clarified that PG would cover clinical and sub-clinical levels. In fact, we refrain from categorizing participants based on their PG score, but rather use it as a graded scale of severity in line with the reviewer points. Although we appreciate the Hazardous gaming terminology, we refrained from introducing it in the present paper, as there are already many distinctions the reader needs to maintain, especially concerning the regularity of gaming - which is the focus of this work. This being said, we have amended introduction and discussion to clearly convey that PG provides self-reports about the severity of problematic gaming.

We use the total score of the 10 items, including the 9 of DSM-IV-GD items that match the ones of the DSM-V-IGD (see table S6) plus the co-dependence item which is not present in the DSM-V-IGD. We now provide in a novel table S6, the mapping between the PG items used here (i.e., the 10 items from DSM-IV) and the IGD items (i.e., 9 items of the DSM-VIGD), with the exact wording used in the present study, and their corresponding labels as per the DSM-V-IGD.

Table S6 makes clear that the DSM items are not worded as questions but rather as descriptions of symptoms, leaving some flexibility regarding the exact wording used. Hence the importance of publishing our exact wording. We thank the reviewers for calling our attention to some mislabeling in our previously submitted versions. These have now been corrected as shown in Table S6. The questions used in our study do cover the 9 symptoms that the DSM-V IGD recommends covering for clinical diagnosis, plus the co-dependence item. We have rewritten this section of the introduction following the reviewer’s recommendation: *“While PG and IGD rely on a similar screening tool (with 9 of the10 PG scale questions being shared with the IGD scale), PG only aims at capturing the severity of self-reported symptoms around gaming, making it amenable to clinical and sub-clinical populations, when IGD refers to a clinical diagnosis. Accordingy, the focus of this work on that broader construct of PG and relies on self-reported PG rather than clinical diagnosis, as is common in the field.”*

**Table S6*.*** Items of the DSM-IV and PG scale as compared to description of DSM-V for IGD diagnosis. The questions used in our study covers the 9 symptoms of the DSM-V criteria for IGD. The 10th item, co-dependence, is present in the DSM-IV / PG but absent from DSM-V / IGD.

| **# IGD item** | **DSM IV Item** | **IGD description** | **PG wording used in this study** |
| --- | --- | --- | --- |
| 1 | **Preoccupation** | The person is preoccupied with gaming. | In the past year, are you thinking about computer or video games more and more? |
| 2 | Withdrawal | The person experiences withdrawal symptoms when gaming is not possible. | In the past year, have you become restless or irritable when trying to cut down or stop playing computer or video games? |
| 3 | **Tolerance** | The person needs to spend more time gaming to satisfy their urge. | In the past year, do you need to spend more and more time or money on computer/video games to feel the same amount of excitement? |
| 4 | **Loss of control** | The person has made unsuccessful attempts to cut down or stop gaming. | In the past year, have you tried to play computer or video games less often or for shorter periods of time? are you successful? |
| 5 | **Loss of interest** | The person has lost interest in other hobbies and entertainment as a result of, or because of, gaming. | In the past year, have you ever skipped your studies or co-curricular activities (CCA) to play more computer or video games? |
| 6 | **Continuation** | The person has jeopardized or lost a significant relationship, job, educational or career opportunity because of gaming. | In the past year, has your schoolwork suffered because you spent too much time playing computer or video games? |
| 7 | Deception | The person has used deception with family members, therapists, or others about the extent of their gaming. | In the past year, have you ever lied to family or friends about how much you play computer or video games? |
| 8 | Escape | The person has used gaming to escape negative moods or feelings. | In the past year, have you played computer or video games to escape from problems, bad feelings, or stress? |
| 9 | Jeopardizing Relationships or Career | The person has jeopardized or lost a job or relationship due to gaming | In the past year, have you ever stolen a video game from a store or a friend, or stolen money so you could buy or play a video game? |
| **-** | **Codependence** | **NOT IN IGD** | **In the past year, have you ever needed to borrow money so you could get or play computer or video games?** |

8. Age of data. I cannot find where the authors have addressed the age of the data itself and provided a justification for using data so old. The authors need to address this issue.

Issue: It is not sufficient to say that the measures and methodology are reported elsewhere. This paper must stand alone in terms of describing the sample and measures used here. Given the contentiousness of claiming IGD was measured (see below) it is crucial that all measures be made clear and described in sufficient detail. It is also important that key sample characteristics are reported.

Response: We have moved the details about the sample and the measures from the supplementary information to the methods section of the main manuscript.

**Adequacy of author response: Resolved**

9. Issue: It is also purported that the study measured IGD. This cannot be factually true given that no such diagnosis existed at the time of the study.

Response: This important point is now clarified in the introduction, where we note that we use the term problematic gaming in this work to cover PG, GD and IGD, as the DSM scales have changed.

**Adequacy of author response: Resolved**

Results

10. Issue: Should decimals be to two decimal points (e.g., Table 2)

Response: We have added the second decimals that were missing.
 **Adequacy of author response: Resolved**

11. Issue: I think it is important to note that the IGD score is [presumably – we never see the measure] a score of IGD symptomology providing a continuous numeric measure on a continuum, rather than an indication of actually having a gaming disorder. So this is not really a study of IGD or any sort of disorder in a way, it is a study of the level of symptoms, with low levels at one end and higher levels at the other, with no way of knowing which, if any, participants had a gaming disorder. In line 335 diagnosis of IGD is discussed in relation to the data. Again, these data predate the diagnosis or any measure of IGD and the data are continuous without categorising IGD.

Response: We have clarified that the dependent variable is the sum of all 10 items, which are available in Table S2, and represents a measure of the severity of PG symptomatology.

**Adequacy of author response: Resolved**

12. Issue: Paragraph starting line 248. It is not simply the case that novel gamers had some IGD symptoms at wave 1 – their levels were nearly as high as at wave 3 and nearly as high as all other groups. Indeed higher than the ‘low’ group. This does not seem to be entirely an artefact of the wording or timing of presentation of measures – for me it is a significant anomaly that casts doubt on the measure of IGD and all the IGD data.

Adequacy of author response: Not resolved. I do not think this explanation is sufficient or clear. Why would kids who only play outside the house, and have less opportunity to play, have PG symptoms close to those of intermediate and heavy gamers? Why would gaming only outside the home result in substantial problems with loss of control over gaming and preoccupation? Is there any reason this group would have disproportionately high loss of other interests or gaming for withdrawal? Why would their gaming hours be the same as intermediate gamers and close to that of heavy gamers when they have less opportunities to play? Something still seems wrong. Maybe the problem is the categorisation – perhaps this group aren’t really novel gamers? Is there a cultural explanation – parenting is so strict in Singapore many kids spend excessive time out of the home gaming? (If so, is there evidence of this). Either way, I think this section (lines 372 to 389) needs to be completely re-thought and revised.

**Response:** We thank the reviewer for raising this important point. As noted above, our choice of terminology is likely the source of confusion. Children were asked whether they usually play video games. A negative answer does not mean that these children have never played video games, merely that they don’t usually play. To correctly capture this situation, we have changed the label from “Novel” to “Occasional” Gamers. It should not come as a surprise that, amongst occasional gamers, there are children who have engaged in the past with video game play or are now engaging in video game play, but do not do so usually within their current school or family setting.

We now discuss how occasional gamers may report levels of gaming that are comparable to low gamers at T1.

This has now been addressed lines 388-400 of the revised MS:

*“All questions including those regarding gaming behavior and PG symptoms specified the time interval to be “over the past year”. Children were therefore asked if, during the past year, they usually played video game. Gamers that replied “No”, or occasional gamers as we labeled them, exhibited levels of PG symptoms at wave 1 as high as at wave 3, and nearly as high as all other groups at baseline. Occasional gamers - while signaling they don’t usually play at present - may have played regularly in the past, but stopped due to encountering difficulties, or may be children who are not allowed to play video game at home, and yet may engage in video game play outside of the family setting, signaling an attitude around gaming, which is again rather unhealthy. In either case, these occasional gamers may report on experienced past symptoms. This apparent disconnect aligns with their most frequently reported “symptoms” at baseline being loss of control / unsuccessful attempts (item 9), followed by escape (item 6).”*

13. Issue: Overall I found the results hard to follow and interpret, but having said that do think the findings are interesting and publishable.

Response: We thank the reviewer and have rephrased the results and discussion to make them easier to follow.

**Adequacy of author response: Resolved (except issues otherwise noted)**

Discussion

14. Issue: Line 365. Is ‘remarkable’ the best word for a finding that is strongly in line with past research? Maybe noteworthy ….

We have replaced remarkable with noteworthy

**Adequacy of author response: Resolved**

15. Issue: I am not sure one can categorise IGD as an impulse control problem (line 383). Rather, impulsiveness is one risk factor. It is certainly not an adequate description of the disorder.

Response: We thank the reviewer for this clarification. We have removed this sentence and rephrased part of our text. We agree with the reviewer that it does not provide direct evidence for considering PG as an impulse control problem.

**Adequacy of author response: Resolved**

16. Issue: Overall I found the discussion to be lacking in depth, entirely omitting the clear limitations of the paper or adequately situating findings in existing literature.

Response: The discussion now better situates our findings within the existing literature. We have also added a limitation section. In particular, we now emphasize that the direction and magnitude of the observed effects is consistent with the prior work discussed in the introduction. We also acknowledge the inherent limitations of our measures of family environment and of impulsivity, especially when it comes to capturing more complex dynamics associated with the constructs of social environment and of impulse regulation.

Adequacy of author response: The Discussion is much improved. I would note (lines 554-566) that a recent meta-analysis (Moshel et al 2024) found links between gaming disorders and reduced executive function. Whilst the biggest effect was on attention, global EF including inhibitory control was also impacted. Thus, whilst problematic gaming over time seems unlikely to change trait impulsivity per se, it seems possible it may increase inhibitory control problems, which would look like greater impulsivity over time, similar to the finding here.

**Response:** We thank the reviewer for bringing to our attention the Moshel meta-analysis which is indeed quite relevant to conceptualizations and measures of, on the one hand, impulsivity via surveys and, on the other hand, inhibitory control via cognitive tasks. It is now discussed on lines 578-601 of the discussion.

*“Another point concerns the nature of the inhibitory control construct under study when looking at PG and impulsivity. A recent meta-analysis of 43 cross-sectional studies of disordered screen use (Moshel et al., 2024) reported small-to-medium cognitive deficits relative to controls (overall Hedges' g = 0.38), with attention being the most affected domain (g = 0.50) and the broad executive-functioning domain showing a smaller but significant deficit (g = 0.31). Within executive functioning, the strongest signals came from delay discounting (g = 0.59) and Stop-Signal-task performance (g = 0.52), while Go/No-go reaction times did not differ between disordered users and controls — indicating that the relationship between PG and inhibitory control is task-dependent rather than uniform. A complementary meta-analysis specifically targeting response inhibition in Internet Gaming Disorder reached a similar conclusion (Argyriou et al., 2017), and an independent meta-analysis of cognitive deficits in problematic internet use likewise reports the strongest deficits in inhibitory control and decision-making (Ioannidis et al., 2019). Two caveats are important. First, the available evidence is overwhelmingly cross-sectional, so it cannot, on its own, establish whether PG induces these deficits or whether pre-existing deficits predispose to PG — a question for which longitudinal designs such as ours are well-suited. Second, the trait-level impulsivity assessed by the BIS (used here) and the task-level inhibitory control captured in most meta-analyses are conceptually and psychometrically distinct (Sharma et al., 2014). Taken together, these results suggest that PG may not necessarily change trait impulsivity itself but may be accompanied by — and possibly amplify — difficulties in task-level inhibitory control, which our self-report measure of impulse-control problems would tend to pick up as elevated impulsiveness over time. This is consistent with our finding of bi-directional change-on-change effects between impulsivity and PG specifically in heavy gamers.”*

**References**

Argyriou E, Davison CB, Lee TTC. Response inhibition and Internet gaming disorder: A meta-analysis. Addictive Behaviors. 2017;71:54–60. doi:10.1016/j.addbeh.2017.02.026. — Specifically on inhibitory control in IGD; directly relevant.

Ioannidis K, Hook R, Goudriaan AE, et al. Cognitive deficits in problematic internet use: meta-analysis of 40 studies. British Journal of Psychiatry. 2019;215(5):639–646. doi:10.1192/bjp.2019.3. — Independent meta-analysis confirming inhibitory-control and decision-making deficits.

Sharma L, Markon KE, Clark LA. Toward a theory of distinct types of "impulsive" behaviors: A meta-analysis of self-report and behavioral measures. Psychological Bulletin. 2014;140(2):374–408. doi:10.1037/a0034418. — The classic reference for the trait-vs-task-level impulsivity distinction; useful to ground the BIS-vs-Stop-Signal contrast.

Minor issues

Issue: There is often not a gap between a word and a following reference number in brackets.

Response: Missing spaces have been added.

Adequacy of author response: Resolved


In Summary

Issue: This paper has a number of serious issues – the age of the data, the measure of IGD predating the existence of the diagnosis, the lack of detail about key parts of the study, and a lack of clarity and focus across the paper. I do think the results are interesting but would need to be presented in a heavily redrafted paper with a completely new focus (not IGD).

Response: We thank the reviewer for their thoughtful and constructive feedback on our manuscript. We hope our interesting results are now better contextualized thanks to the novel introduction, the clarification about the collected measures, the amount of missing data and attrition rates across waves and measures, as well as the updated discussion. We thank the reviewer for their suggestions as to how to strengthen our paper.

**Adequacy of author response: The authors have gone to considerable trouble to revise this paper and have generally done a quite good job. There are still some issues that I think need to be resolved before this can be published, with a better framing of PG crucial, but once these issues are resolved I think this paper will make an interesting contribution to the field.**

**Response:** We thank the reviewer for his positive feedback on our responses and have amended the introduction to better frame PG, IGD and other terminologies around gaming disorder.

Reviewer #3: Thank you for the opportunity to review this manuscript. The study addresses an interesting question about whether the associations between established risk factors and problematic gaming (PG) symptoms are moderated by baseline gaming habits in a longitudinal sample. The research question is timely, the sample size is substantial, and the application of Specification Curve Analysis is a notable methodological strength.

However, I have a number of concerns, some of which are substantial, that I believe need to be addressed.

1. The manuscript does not disclose in either the title or the abstract that this is a secondary analysis of an existing longitudinal dataset originally collected between 2007 and 2009 (Gentile et al., 2011; Choo et al., 2010). This is an important transparency issue. Readers should be made aware from the outset that the study design, measures, and sampling decisions were made for a different primary purpose, and that the current analyses were not pre-specified in that original study. PLOS ONE and broader reporting standards for secondary analyses require this to be clearly stated. The abstract also does not report basic methodological details, including sample size, country of origin, study period, or the key inclusion criteria, which would be expected of a methods-transparent abstract. Both issues should be corrected.

**Response:** We have now clarified in the title and abstract that the work is based on a reanalysis of a historical dataset.

2. The VG hours composite was derived by multiplying self-reported hours on a typical weekday by five and on a typical weekend day by two. This approach risks overestimating weekly gaming time for participants who do not game every day, since multiplying a 'typical play session' figure across all days of a given type attributes gaming to days when no gaming may have occurred. A participant who games on three of five weekdays for two hours each session would report a 'typical weekday' figure of two hours, yielding a composite weekday contribution of ten hours rather than the actual six. Second, and more importantly, the relationship between this self-reported composite and actual gaming behavior is likely noisy. Research has shown that self-reported gaming time correlates only moderately with PG symptoms (i.e., r = 0.25–0.50 in comprehensive reviews; King et al., 2020 <https://doi.org/10.1016/j.cpr.2020.101831>) and, critically, that self-reported gaming time correlates more strongly with available leisure time than with problematic patterns of play (Stevens et al., 2025, Addiction, <https://doi.org/10.1111/add.70192>). The authors should acknowledge this limitation explicitly and consider its implications for interpreting the finding that VG hours drives PG change in novel gamers.

**Response:** We thank the reviewer for raising this limitation of most self-reported measures of behaviors using frequency or time-spent metrics. We have acknowledged the limitation in the manuscript and added the relevant citations to the discussion, lines 696-713: *“Firth, our measure of gaming consumption is based on self-reported frequency of gaming using typical‑day composite (typical weekday × 5 + typical weekend day × 2), which can overestimate weekly hours for participants who do not play every day and is, more broadly, an imperfect index of harm. Across screening and assessment instruments, gaming time correlates only modestly with gaming‑disorder symptom scores (largely r ≈ 0.2–0.4; see the systematic review by King et al., 2020), and a recent psychometric evaluation indicates that self‑reported gaming‑time measures track available leisure time more closely than they track problematic patterns of play (Stevens et al., 2026). This is consistent with our finding that changes in VG hours account for a relatively modest share of variance in PG change. This point is particularly relevant for the interpretation of the larger VG‑hours → PG association observed in occasional gamers, as partly reflecting baseline differences in leisure‑time availability and engagement rather than a specific symptomatic intensification. As many others have pointed out, more objective measures of game consumption would be preferable."*

**References**

King DL, Chamberlain SR, Carragher N, Billieux J, Stein D, Mueller K, et al. Screening and assessment tools for gaming disorder: a comprehensive systematic review. Clinical Psychology Review. 2020;77:101831. doi:10.1016/j.cpr.2020.101831.

Stevens MWR, Radünz M, Galanis C, Quinney B, Zajac I, Billieux J, et al. Red Box, Green Box: Psychometric evaluation of a self-report behavioral frequency measurement approach for behavioral addictions research. Addiction. 2026;121(2):429–39. doi:10.1111/add.70192

3. In the introduction (line 97, approximately), the authors refer to an effect size of r = 0.29 as "small-to-moderate." Yet, on another occasion they describe a pooled longitudinal r = 0.31 as a "strong" correlation for gaming time predicting PG. These two characterisations are inconsistent with each other and, more broadly, r = 0.31 is not conventionally regarded as a strong effect. A citation is needed to justify calling this effect strong, and the authors should apply consistent terminology throughout.

**Response:** The different interpretations reflect differences in magnitudes between effect size in cross-sectional and longitudinal designs. An r = 0.30 is moderate for a cross-sectional correlation, but large for a longitudinal (cross-lagged) association. We have added a citation to Orth et al., (2024) who report beta values of .03, .07, and .12 as benchmarks for small, medium and large effects respectively in longitudinal cross-lagged (CLPM and RICLPM) analyses. This has been clarified in the introduction, lines 76-79: *“The pooled correlation for gaming time predicting subsequent symptoms is strong (r = 0.31) in longitudinal studies [9], according to the benchmarks for interpreting longitudinal (cross-lagged) effects (0.03, 0.07 and 0.12 respectively for small, medium and large effects; Orth et al., 2024.”*

**References**

Orth U, Meier LL, Bühler JL, Dapp LC, Krauss S, Messerli D, et al. Effect size guidelines for cross-lagged effects. Psychological Methods. 2024;29(2):421–33. doi:10.1037/met0000499

4. The Cronbach's alpha for the PG scale is reported as 0.70 (95% CI: 0.67–0.72), which is generally considered borderline acceptable and, at the lower confidence interval bound, would fall into the range many methodologists would flag as problematic. The authors note this in passing but do not pursue the implications sufficiently. Given the unusual item composition of this scale, particularly the inclusion of a "sneaky behaviors" item drawn from DSM-IV pathological gambling (rather than any IGD framework), it would be worth examining whether this item is responsible for attenuating the alpha and whether removing it and re-running the core analyses would alter the findings. Additionally, the authors should report omega coefficients (e.g., McDonald's omega) rather than or alongside Cronbach's alpha, as omega is a more robust reliability estimate that does not assume tau-equivalence and is now preferred in the psychometric literature. This goes for all scales used in this study.

**Response:** We thank Reviewer 3 for this thoughtful comment, which prompted us to clarify three points: (i) the item composition and labelling of the PG scale, (ii) the empirical value of α ≈ 0.70 in the context of brief, DSM-derived symptom inventories, and (iii) the reliability of all scales using McDonald’s omega in addition to Cronbach’s alpha.

**(i) Item composition and the “sneaky behaviors” item.** Our PG scale comprises the 10 items derived from the DSM-IV criteria for pathological gambling, which historically formed the basis for the 9 DSM-V IGD criteria. The item previously labelled “sneaky behaviours” in our manuscript was, in fact, a mis-labelling: this item taps the “jeopardize / lying to family members” dimension that is also present in the DSM-V IGD criteria. The single DSM-IV item that was not retained in the DSM-V IGD criteria is co-dependence (i.e., needing to borrow money to game). The full mapping between the DSM-IV PG and DSM-V IGD criteria, together with the exact item wording used in our questionnaire, is now provided in Table S6, and the labels in Figure 3 have been corrected accordingly. The scale and its scoring are otherwise unchanged from the version used in the original Gentile (2009) and Choo et al. (2010) studies, which were conducted prior to the publication of DSM-V and have served as the basis for multiple peer-reviewed analyses of this Singaporean cohort.

**(ii) The value of α ≈ 0.70 in this measurement context.** We respectfully note that α = 0.70 (95% CI 0.67–0.72) is fully in line with the values reported for brief DSM-based gaming-disorder instruments. A few points are worth emphasising.

First, Cronbach’s α is mechanically sensitive to the number of items and to multidimensionality, both of which apply here. With only 10 binary items, each tapping a distinct DSM diagnostic criterion (preoccupation, withdrawal, tolerance, loss of control, etc.), the scale is not designed to be unidimensional or tau-equivalent. As is well established (Cortina, 1993; Streiner, 2003; Sijtsma, 2009), α systematically under-estimates reliability of short, multidimensional symptom inventories. Second, as the reviewer states with an alpha of .7 we are indeed at the lower range of what one wishes to attain for validating the internal consistency of a set of test items. Yet, we respectfully note that the oft cited “α ≥ 0.70” heuristic, sometimes attributed to Nunnally (1978), was originally intended as a guideline for early-stage research rather than a strict cut-off (Lance, Butts & Michels, 2006; Taber, 2018).

Third and arguably most relevant, the values we report are in line with — and in several cases exceed — those reported for the most widely used IGD instruments based on the same DSM criteria. To give a few benchmarks (all in adolescent or young-adult samples):

- Lemmens, Valkenburg & Gentile (2015) — 9-item Internet Gaming Disorder Scale (IGDS), based on DSM-V IGD criteria: α = 0.81 (Dutch) and 0.82 (US).
- Pontes & Griffiths (2015) — IGDS9-SF: α = 0.87 in adult gamers, but markedly lower in adolescent and child samples.
- Király et al. (2017) — Ten-Item Internet Gaming Disorder Test (IGDT-10): α = 0.72 in Hungarian adolescents.
- Király et al. (2019) — cross-cultural validation of IGDT-10 across 9 countries: α ranged from 0.69 to 0.85.
- Paschke, Sack & Thomasius (2021) — IGDS9-SF in three large German child/adolescent samples: α = 0.56 (2016), 0.72 (2017), 0.78 (2018).
- In the foundational DSM-IV gambling literature from which our scale is derived, Stinchfield (2003) reports α = 0.87 in a general sample but values dropping into the 0.65–0.75 range in adolescent and lower-base-rate subsamples.
- The comprehensive systematic review by King et al. (2020) likewise documents that reported reliability for IGD/GD screening instruments commonly falls in the 0.70–0.85 range, and frequently below 0.70 in younger or lower-symptom samples.

In sum, our reported α of 0.70 is well within the expected range given the existing literature. . We have added a sentence in the Methods/Measures and in the Limitations clarifying this point, with references.

**We have added the following text to the revised Methods, lines 244-249:** *“The observed α, while in the lower acceptable range, is in line with values reported for the most widely used DSM-based gaming-disorder instruments in adolescent samples (e.g., α = 0.81–0.82 for the 9-item Internet Gaming Disorder Scale, (Lemmens et al., 2015); α = 0.72 for the IGDT-10 (Király et al., 2017); α = 0.69–0.85 across 9 countries in the IGDT-10 cross-cultural validation, (Király et al., 2019); α = 0.56–0.78 across three German child/adolescent waves for the IGDS9-SF, (Paschke et al., 2021); see also King et al., 2020).*

**(iii) McDonald’s omega and the role of the co-dependence item.** Following the reviewer’s recommendation, we now report McDonald’s ω total alongside Cronbach’s α for all multi-item scales. We thank the reviewer for this suggestion as indeed ω does not require the tau-equivalence assumption (Dunn, Baguley & Brunsden, 2014; Hayes & Coutts, 2020) and is therefore the more appropriate reliability index for symptom scales. For the full 10-item PG scale, ω = 0.75 (vs. α = 0.70). Removing the co-dependence item — which is the one DSM-IV PG item not retained in the DSM-V IGD criteria — barely affected these indices (α = 0.69, 95% CI 0.67–0.71; ω = 0.74), confirming that this item is not responsible for attenuating reliability. Moreover, the item-level analyses in Figure 3 (updated) show that the co-dependence item explains a negligible share of the variance in any group. For consistency, Omega values have likewise been added for impulsivity and family environment.

**Benchmark McDonald's ω values for IGD/GD instruments.** Until recently, omega has been reported less consistently than alpha in the IGD/GD psychometric literature — the comprehensive systematic review by King et al. (2020) does not tabulate omega at all — but where it has been reported, values for brief DSM-5 / ICD-11–based instruments cluster in roughly the same range as α (typically ω ≈ 0.80–0.92 in adult validation samples, lower in adolescents). To give a few benchmarks:

- Monacis, de Palo, Griffiths & Sinatra (2016) — IGDS9-SF Italian validation: composite reliability / ω ≈ 0.87 from the unidimensional CFA (adult sample).
- Severo, de Souza Vasconcellos, Rolim Medeiros et al. (2020) — IGDS9-SF Brazilian validation: ω ≈ 0.88 alongside α ≈ 0.87.
- Stavropoulos, Beard, Griffiths et al. (2018) — IGDS9-SF cross-cultural (US/Australia): composite reliability ≈ 0.85–0.90 across samples.
- Pontes, Schivinski, Sindermann, Li, Becker, Zhou & Montag (2021) — Gaming Disorder Test (GDT, 4 items, ICD-11): ω in the 0.85–0.92 range across multi-country samples.
- Paschke, Sack & Thomasius (2021) — IGDS9-SF in three large German child/adolescent samples: where ω is reported alongside α, values are comparable to α (0.56–0.78 across waves), confirming the pattern that brief scales in younger samples produce lower reliability for both indices.

Our ω = 0.75 for a 10-item, multi-symptom, DSM-IV-derived scale in 11–13-year-olds at baseline therefore sits at the lower end of the range reported for adult IGDS9-SF / GDT validation samples and within the range observed in adolescent samples (Paschke et al., 2021). As expected from the theoretical properties of the two coefficients (Dunn, Baguley & Brunsden, 2014; Hayes & Coutts, 2020), ω exceeds α by about .05 in our data, consistent with the scale being multidimensional rather than tau-equivalent.

**We have added the following text to the methods section, lines 250-256:** *“We further report McDonald's ω for all multi-item scales throughout the manuscript as, unlike α, it does not assume tau-equivalence and is therefore more appropriate for brief DSM-derived symptom inventories like ours (Dunn et al., 2014; Hayes & Coutts, 2020). McDonald's ω total for the same 10-item scale was 0.75, which is again in the low acceptable end of the range reported for adult IGDS9-SF / GDT validation samples and within the range observed in adolescent samples (Paschke et al., 2021). Finally, removing the single DSM-IV item not retained in the DSM-V IGD criteria (co-dependence) did not improve reliability (α = 0.69; ω = 0.74).”*

**References**

Cortina JM. What is coefficient alpha? An examination of theory and applications. Journal of Applied Psychology. 1993;78(1):98–104. doi:10.1037/0021-9010.78.1.98.

Dunn TJ, Baguley T, Brunsden V. From alpha to omega: a practical solution to the pervasive problem of internal consistency estimation. British Journal of Psychology. 2014;105(3):399–412. doi:10.1111/bjop.12046.

Hayes AF, Coutts JJ. Use omega rather than Cronbach’s alpha for estimating reliability. But… Communication Methods and Measures. 2020;14(1):1–24. doi:10.1080/19312458.2020.1718629.

King DL, Chamberlain SR, Carragher N, Billieux J, Stein D, Mueller K, et al. Screening and assessment tools for gaming disorder: a comprehensive systematic review. Clinical Psychology Review. 2020;77:101831. doi:10.1016/j.cpr.2020.101831.

Király O, Sleczka P, Pontes HM, Urbán R, Griffiths MD, Demetrovics Z. Validation of the Ten-Item Internet Gaming Disorder Test (IGDT-10) and evaluation of the nine DSM-V Internet Gaming Disorder criteria. Addictive Behaviors. 2017;64:253–260. doi:10.1016/j.addbeh.2015.11.005.

Király O, Bőthe B, Ramos-Diaz J, Rahimi-Movaghar A, Lukavska K, Hrabec O, et al. Ten-Item Internet Gaming Disorder Test (IGDT-10): measurement invariance and cross-cultural validation across seven language-based samples. Psychology of Addictive Behaviors. 2019;33(1):91–103. doi:10.1037/adb0000433.

Lemmens JS, Valkenburg PM, Gentile DA. The Internet Gaming Disorder Scale. Psychological Assessment. 2015;27(2):567–582. doi:10.1037/pas0000062.

Monacis L, de Palo V, Griffiths MD, Sinatra M. Validation of the Internet Gaming Disorder Scale – Short-Form (IGDS9-SF) in an Italian-speaking sample. Journal of Behavioral Addictions. 2016;5(4):683–690. doi:10.1556/2006.5.2016.083.

Paschke K, Sack PM, Thomasius R. Validity and psychometric properties of the Internet Gaming Disorder Scale in three large independent samples of children and adolescents. International Journal of Environmental Research and Public Health. 2021;18(3):1095. doi:10.3390/ijerph18031095.

Pontes HM, Griffiths MD. Measuring DSM-V Internet gaming disorder: development and validation of a short psychometric scale. Computers in Human Behavior. 2015;45:137–143. doi:10.1016/j.chb.2014.12.006.

Pontes HM, Schivinski B, Sindermann C, Li M, Becker B, Zhou M, Montag C. Measurement and conceptualization of Gaming Disorder according to the World Health Organization framework: the development of the Gaming Disorder Test. International Journal of Mental Health and Addiction. 2021;19(2):508–528. doi:10.1007/s11469-019-00088-z.

Severo RB, Soares JM, Affonso JP, Giusti DA, de Souza Junior AA, de Figueiredo VLM, et al. Prevalence and risk factors for Internet Gaming Disorder. Brazilian Journal of Psychiatry. 2020;42(5):532–535. doi:10.1590/1516-4446-2019-0760.

Stavropoulos V, Beard C, Griffiths MD, Buleigh T, Gomez R, Pontes HM. Measurement invariance of the Internet Gaming Disorder Scale – Short-Form (IGDS9-SF) between Australia, the USA and the UK. International Journal of Mental Health and Addiction. 2018;16(2):377–392. doi:10.1007/s11469-017-9786-3.

Streiner DL. Starting at the beginning: an introduction to coefficient alpha and internal consistency. Journal of Personality Assessment. 2003;80(1):99–103. doi:10.1207/S15327752JPA8001_18.

Sijtsma K. On the use, the misuse, and the very limited usefulness of Cronbach's alpha. Psychometrika. 2009;74(1):107–120. doi:10.1007/s11336-008-9101-0.

5. The PG measure is derived from DSM-IV pathological gambling criteria and was administered between 2007 and 2009. It predates the DSM-V IGD criteria (2013) and the ICD-11 Gaming Disorder definition (2019). The alignment between this instrument and current diagnostic frameworks is therefore partial at best. Nine of the ten items reportedly correspond to IGD criteria, but the tenth item (sneaky behaviors) does not, and this item appears to drive a notable proportion of the VG hours effect in novel gamers (Figure 3). The authors acknowledge this in the item-level exploratory analysis but do not adequately address the construct validity concern in the main methods or limitations sections. Reviewers and readers of a 2025/2026 manuscript should be made aware that the core outcome measure was not designed or validated against either current diagnostic system.

**Response:** We thank Reviewer 3 for pressing this important point. Our revisions address three related concerns: (i) the historical continuity between the scale we used and the DSM-V IGD criteria, (ii) the corrected item-level mapping (including the previously mis-labelled “sneaky” item), and (iii) the explicit construct-validity statement now added to the Methods and Limitations.

**(i) Historical continuity and construct alignment.** The DSM-V IGD criteria were not developed independently of the DSM-IV pathological gambling (PG) criteria — they were explicitly modelled on them, with the DSM-V work-group adapting nine of the ten DSM-IV PG criteria to the gaming context (Petry et al., 2014; American Psychiatric Association, 2013). Our scale uses these same 10 DSM-IV PG items, the 9 of which that were retained in DSM-V IGD therefore map onto the DSM-V IGD criteria with a one-to-one correspondence (see Table S6 for the full mapping, with the exact item wording used in our questionnaire). The only DSM-IV PG item not retained in DSM-V IGD is co-dependence (i.e., needing to borrow money to game), not “sneaky behaviour” as we had erroneously suggested in the previous version of the manuscript (see below). As such, the construct captured by our composite is not partial alignment with DSM-V IGD — it is DSM-V IGD symptomatology plus one additional item, and we treat it accordingly throughout the manuscript.

**(ii) Item-level correction (“sneaky” → “jeopardize”).** We are grateful to the reviewer for prompting us to re-examine the item labels. The item previously labelled “sneaky” in our manuscript and figures was, in fact, a mis-labelling: the wording of the item (“In the past year, have you ever lied to family or friends about how much you play computer or video games?”) corresponds directly to the DSM-V IGD criterion of deception / lying to family members about extent of involvement (jeopardize-relationships / lying dimension). It is the DSM-IV co-dependence item — not the deception item — that has no DSM-V IGD analogue. The mis-labelling has been corrected in Table S6 (with both the erroneous and corrected labels shown for transparency), in Figure 3, and throughout the manuscript text. With the corrected labelling, the item that contributes most variance to the VG-hours→PG effect in occasional gamers is the jeopardize item, which is fully part of the DSM-V IGD criteria; the co-dependence item — the one item without a DSM-V IGD analogue — contributes negligibly in every group (updated Figure 3).

**(iii) Scale provenance and prior use.** The scale has been used in multiple peer-reviewed publications on this same Singaporean cohort (Gentile et al., 2011; Choo et al., 2010; Liau et al., 2015; Choo et al., 2015) as well as in Gentile’s (2009) earlier US national study. The instrument therefore has a substantial published track record, including the same author group, in studies whose target construct is gaming disorder severity. We have added a sentence in the Methods making this lineage explicit.

**(iv) New Methods and Limitations text.** Reflecting the above, we have added the following clarifications:

**Methods / Measures (lines 230-237):** *“Participants answered the 10-item screening instrument derived from the pathological gambling items of the DSM-IV, and adapted to PG. Such a 10-item DSM-IV-derived composite for PG has been validated and used in multiple peer-reviewed analyses of this and related cohorts (Gentile, 2009; Choo et al., 2010; Gentile et al., 2011; Liau et al., 2015; Choo et al., 2015). TThe DSM-V IGD criteria (APA, 2013) were themselves developed by adapting nine of the ten DSM-IV pathological-gambling criteria to gaming (Petry et al., 2014); accordingly, nine of our ten items map onto the DSM-V IGD criteria (Table S6). The remaining item (co-dependence — needing to borrow money to game) is the one DSM-IV PG item without a DSM-V IGD analogue.”*

**References**

Petry NM, Rehbein F, Gentile DA, Lemmens JS, Rumpf H-J, Mößle T, et al. An international consensus for assessing internet gaming disorder using the new DSM-V approach. Addiction. 2014;109(9):1399–1406. doi:10.1111/add.12457.

• Gentile DA. Pathological video-game use among youth ages 8 to 18: A national study. Psychological Science. 2009;20(5):594–602. doi:10.1111/j.1467-9280.2009.02340.x.

• Liau AK, Choo H, Li D, Gentile DA, Sim T, Khoo A. Pathological video-gaming among youth: a prospective study examining dynamic protective factors. Addiction Research & Theory. 2015;23(4):301–308. doi:10.3109/16066359.2014.987759.

• Choo H, Sim T, Liau AK, Gentile DA, Khoo A. Parental influences on pathological symptoms of video-gaming among children and adolescents. Journal of Child and Family Studies. 2015;24(5):1429–1441. doi:10.1007/s10826-014-9949-9.

6. The manuscript examines family environment as the primary interpersonal predictor but does not consider peer-level factors. This is a meaningful gap given the extensive literature on the role of school belonging, peer victimization, online social dynamics, and social isolation as risk factors for PG, particularly in Chinese and East Asian adolescent samples. A number of studies using similar Singaporean and Chinese cohorts have highlighted that peer and school factors often explain variance in PG beyond family environment. The authors should at minimum acknowledge this limitation and note that the absence of peer-level measures constrains the generalizability of the conclusion that interpersonal factors show relatively weak effects.

**Response:** We fully agree with the reviewer that peer-level and school-level factors — including peer victimization, social isolation, peer influence, and school belonging — are well-documented risk and protective factors for problematic gaming (PG), with particularly strong evidence in East Asian adolescent samples (e.g., Yu & Shek, 2013, Hong Kong; Yu, Li & Zhang, 2015 and Zhu, Zhang, Yu & Bao, 2015, mainland China; for syntheses, see Schneider, King & Delfabbro, 2017; Mihara & Higuchi, 2017; Sugaya et al., 2019). Importantly, no validated peer- or school-belonging measures were collected, and so they could not be incorporated in the present analyses.

Our choice to focus on the family environment in the present work was driven by three considerations. First, our analyses were structured around the epidemiological triad (agent / host / environment), with one variable per layer; family functioning represents the most proximal modifiable component of the social environment for the early-adolescent age range examined here (most participants aged 11–13 at baseline). Second, the original cohort (Gentile et al., 2011; Choo et al., 2010) was not designed to test peer or school dynamics. Validated multi-item measures of school belonging, peer victimization, or online social dynamics were not collected, so we could not substitute them for, or add them alongside, the family-environment measure. Third, family-environment items were drawn from a well-validated instrument and were available across all three waves — a prerequisite for the within-person, change-on-change analyses on which our main inferences rest.

We agree, however, that the absence of peer- and school-level measures has two specific implications that should be made explicit, and we have revised the Discussion / Limitations accordingly.

- It limits the generalizability of our conclusion that interpersonal factors show relatively weak effects on PG change. Because we measured only one facet of the interpersonal environment (family), our estimate of the contribution of interpersonal factors as a whole is, by construction, a lower bound. Studies that included peer and school constructs have reported associations with PG of magnitudes comparable to — and sometimes exceeding — those of family environment (Yu & Shek, 2013; Yu, Li & Zhang, 2015; Zhu et al., 2015; Schneider et al., 2017). The pattern we observe should therefore be interpreted as showing that family environment specifically explains relatively little variance in change in PG, not that the interpersonal layer as a whole is unimportant.
- It also constrains the cultural interpretation of our results. In East Asian adolescent contexts, peer comparison, school engagement and academic pressure are well-documented amplifiers of PG risk (Yu et al., 2015; Zhu et al., 2015; Mihara & Higuchi, 2017). Our Singaporean cohort therefore likely under-samples some of the most salient interpersonal risk and protective factors for this population, and the relative weight of family vs. peer/school environments may differ in samples where peer-level constructs are explicitly measured.

We have thus revised the discussion accordingly – lines 665-678.

*“Second, among our measures, family environment aimed to capture different aspects of parental behaviour and parent–child relationships. The original cohort was not designed to assess peer- or school-level dynamics (validated measures of school belonging, peer victimization or peer influence were not collected). A substantial body of work — including studies in Hong Kong (L. Yu & Shek, 2013), mainland Chinese (C. Yu et al., 2015; Zhu et al., 2015) and broader East Asian adolescent samples (for syntheses see Schneider et al., 2017; Mihara & Higuchi, 2017; Sugaya et al., 2019) — has documented that peer victimization, peer influence and school engagement are robust risk and protective factors for problematic gaming, sometimes accounting for variance beyond that explained by the family environment. Family environment in our analyses is therefore best interpreted as one facet of the broader interpersonal layer, and our estimate of its contribution to interpersonal factors should be regarded as a lower bound. Future longitudinal work in adolescents would benefit of incorporating peer- and school-level measures alongside family functioning to allow direct comparison of their relative contributions to changes in PG over time.”*

**References**

Choo H, Sim T, Liau AK, Gentile DA, Khoo A. Parental influences on pathological symptoms of video-gaming among children and adolescents: a prospective study. Journal of Child and Family Studies. 2015;24(5):1429–1441. doi:10.1007/s10826-014-9949-9.

Liau AK, Choo H, Li D, Gentile DA, Sim T, Khoo A. Pathological video-gaming among youth: a prospective study examining dynamic protective factors. Addiction Research & Theory. 2015;23(4):301–308. doi:10.3109/16066359.2014.987759.

Mihara S, Higuchi S. Cross-sectional and longitudinal epidemiological studies of Internet gaming disorder: a systematic review of the literature. Psychiatry and Clinical Neurosciences. 2017;71(7):425–444. doi:10.1111/pcn.12532.

Schneider LA, King DL, Delfabbro PH. Family factors in adolescent problematic Internet gaming: a systematic review. Journal of Behavioral Addictions. 2017;6(3):321–333. doi:10.1556/2006.6.2017.035.

Sugaya N, Shirasaka T, Takahashi K, Kanda H. Bio-psychosocial factors of children and adolescents with internet gaming disorder: a systematic review. BioPsychoSocial Medicine. 2019;13:3. doi:10.1186/s13030-019-0144-5.

Yu C, Li X, Zhang W. Predicting adolescent problematic online game use from teacher autonomy support, basic psychological needs satisfaction, and school engagement: a 2-year longitudinal study. Cyberpsychology, Behavior, and Social Networking. 2015;18(4):228–233. doi:10.1089/cyber.2014.0385.

Yu L, Shek DTL. Internet addiction in Hong Kong adolescents: a three-year longitudinal study. Journal of Pediatric and Adolescent Gynecology. 2013;26(3 Suppl):S10–S17. doi:10.1016/j.jpag.2013.03.010.

Zhu J, Zhang W, Yu C, Bao Z. Early adolescent Internet game addiction in context: how parents, school, and peers impact youth. Computers in Human Behavior. 2015;50:159–168. doi:10.1016/j.chb.2015.03.079.

7. The manuscript does not distinguish between pathological engagement and elite or professional gaming, and the heavy-gamer category (averaging 57 hours per week, up to 106 hours) almost certainly includes individuals whose gaming reflects high skill and commitment rather than disorder. Research on professional gamers and eSports athletes shows that this group does not show the same neurobiological or psychosocial patterns as individuals with GD despite comparable gaming hours (see e.g., Choi et al., 2021; <https://doi.org/10.2196/25793>). The authors' framework could be strengthened by acknowledging this heterogeneity within the heavy-gamer group, which may attenuate the observed effect of VG hours on PG in that group.

**Response:** We thank the reviewer for this important point. We fully agree that the heavy-gamer group, defined here solely on the basis of self-reported hours of play, is almost certainly heterogeneous in nature and likely to include adolescents whose extensive engagement reflects passionate, skilled, competitive play as well as disordered use.

We now highlight this limitation, lines 678-686:

*“Third, our gaming-group categorization rests on self-reported weekly hours and does not distinguish between healthy high engagement (e.g., competitive, passionate, or skill-oriented play) and problematic involvement. As a consequence, the heavy-gamer group is intrinsically heterogeneous and likely includes adolescents whose extensive play does not reflect disorder. This is expected to attenuate, rather than inflate, the observed effect of VG hours on PG in that group, and underscores the value of complementing hours-based measures with indicators of motivation, functional impairment, and subjective control in future work. ”*

We have also added a paragraph further discussing possible interpretations given the likely heterogeneity of the heavy-gamers group, lines 601-622. We discuss the systematic review by Choi et al. (2021) as well as the works by Billieux and colleagues (e.g., Deleuze et al., 2018; Billieux et al., 2019)

*“Finally, our heavy-gamer category, defined solely on the basis of self-reported weekly hours (mean ≈ 57 h/week, up to 106 h/week), is likely composed of a mix of profiles, ranging from adolescents engaging in highly committed, skill-oriented, or competitive play to adolescents whose extensive use reflects difficulties in regulation of, and emerging problems with, gaming. Accumulating evidence indicates that these profiles are not interchangeable. In a systematic review of brain-imaging studies, Choi et al., (2021) showed that, despite comparable extensive engagement, professional/elite gamers and individuals with Gaming Disorder display distinct structural and functional alterations. Pro-gamers exhibit enhanced attentional and sensorimotor function as well as better cognitive control, whereas individuals with Gaming Disorder show impaired cognitive control and increased craving-related activation. A complementary line of work led by Billieux and colleagues has long argued for separating high engagement from problematic involvement and has shown that time spent playing, on its own, is a poor proxy for harm (Billieux et al., 2019; Deleuze et al., 2018); see also (Charlton & Danforth, 2007). The fact that, in our data, changes in VG hours explained a smaller share of variance in PG among heavy gamers (~7%) than among occasional gamers (~12%) is consistent with this view. That is, if a non-negligible subset of heavy gamers are highly engaged but not symptomatic, additional hours in this subgroup will be only weakly coupled to PG, attenuating the average VG-hours-to-PG association. Future work using measures that capture the motivational, functional, and contextual aspects of play (e.g., passion, achievement, social, competitive motives; functional impairment; subjective control) in addition to gaming time will be needed to disentangle these distinct trajectories within heavy gamers.”*

**References :**

Choi E, Shin S-H, Ryu J-K, Jung K-I, Hyun Y, Kim J, Park M-H. Association of Extensive Video Gaming and Cognitive Function Changes in Brain-Imaging Studies of Pro Gamers and Individuals With Gaming Disorder: Systematic Literature Review. JMIR Serious Games. 2021;9(3):e25793. doi:10.2196/25793.

Deleuze J, Long J, Liu T-Q, Maurage P, Billieux J. Passion or addiction? Correlates of healthy versus problematic use of videogames in a sample of French-speaking regular players. Addictive Behaviors. 2018;82:114–121. doi:10.1016/j.addbeh.2018.02.031.

Billieux J, Stein DJ, Castro-Calvo J, Higushi S, King DL. Rationale for and usefulness of the inclusion of gaming disorder in the ICD-11. World Psychiatry. 2021;20(2):198–199. doi:10.1002/wps.20848.

Charlton JP, Danforth IDW. Distinguishing addiction and high engagement in the context of online game playing. Computers in Human Behavior. 2007;23(3):1531–1548. doi:10.1016/j.chb.2005.07.002.

8. The observation that novel gamers (who report not usually playing at baseline) nonetheless endorse PG symptoms is acknowledged by the authors but the explanation offered is speculative. It is plausible that this reflects measurement artefact (i.e., children who cannot access games at home but occasionally play elsewhere being classified as non-gamers despite some exposure) but it is equally possible that some participants misunderstood the item ordering or that the "over the past year" framing captures occasional play episodes. This issue creates a conceptual ambiguity at the heart of the novel-gamer subgroup analysis: if novel gamers are reporting PG symptoms without habitual gaming, the construct being measured may be something other than gaming-related harm (e.g., general sensation-seeking, impulsivity, or a response set). The authors should address this more deeply.

**Response:** We now acknowledge this ambiguity regarding the self-reported gaming and PG symptoms of “novel” players which we have now renamed “occasional” players throughout – See response to R1- point 12

9. The authors state they focused on completers (participants with data at waves 1 and 3) and did not apply missing data imputation. Attrition was not uniform across groups, with higher rates among novel gamers. Completers-only analysis in the presence of differential attrition risks producing biased estimates of longitudinal associations, particularly if dropout is related to the outcome (i.e., if adolescents whose PG worsened were more likely to drop out). The authors acknowledge some missing data but do not adequately address whether their completers-only approach may have introduced systematic bias. Multiple imputation or at minimum a sensitivity analysis comparing completers and non-completers on key baseline variables should be considered.

**Response:** We thank the reviewer for raising this important methodological concern. The primary reason for participants dropping out of the study was switching schools. Differential attrition is a genuine threat to the validity of completers-only inferences, and we agree it deserves a more thorough treatment than in the previous version. We have therefore added (i) a missing-data taxonomy clarifying which assumptions are at stake, (ii) a baseline comparison of completers vs. non-completers (new Tables S3 and S4), and (iii) a multiple-imputation sensitivity analysis using chained equations (MICE, m = 20). We summarise each below.

**(i) Missing-data mechanism.** The conventional missing-data taxonomy distinguishes three mechanisms: missing completely at random (MCAR), missing at random (MAR; missingness depends on observed variables but not on the unobserved value itself, conditional on those), and missing not at random (MNAR; missingness depends on the unobserved value). Both maximum-likelihood estimation (used in our CLPM / RI-CLPM models) and MICE are valid under MAR and MCAR, but not under MNAR. The key vulnerability of the completers-only analysis is to MNAR — for example, if adolescents whose PG was worsening were systematically more likely to drop out as the reviewer flags.

**(ii) Completers vs. non-completers comparison.** To probe the plausibility of MNAR, we now compare completers (W1 and W3) and non-completers (W1 only) on all baseline variables (new Tables S3 continuous, S4 categorical). Compared to completers, non-completers reported higher baseline VG hours, higher impulsiveness, slightly more positive family environment, were older, and had lower parental education. Importantly, non-completers reported lower baseline PG, not higher. This pattern is the opposite of the worst-case MNAR scenario invoked in which drop-out is concentrated among adolescents with worsening PG. Thus, if anything, the attrition pattern observed would tend to under- rather than over- estimate the longitudinal VG-hours→PG and impulsivity→PG associations in the completers subsample. Despite this, these two relationships are our strongest effects.

**(iii) Multiple-imputation sensitivity analysis.** As a more direct robustness check, we re-estimated the main change-on-change regressions (changes in PG between W1 and W3 predicted by changes in VG hours, impulsiveness and family environment) after multiple imputation with chained equations (MICE, m = 20). The MICE coefficients are essentially identical to the completers-only estimates (see new figure in SI section 2). In occasional and heavy gamers, the directions, signs and orderings of the effects are unchanged, and the magnitudes differ by less than one standard error. The only notable numerical difference concerns the low-gamer group, in which the impact of VG hours and of family environment on PG is slightly larger after imputation (already a sub-dominant effect in the main analysis). We interpret this convergence as evidence that, within the MAR assumption that MICE makes, the completers-only inferences previously reported are not driven by selection on the outcome. Under more pessimistic MNAR assumptions, no analysis can be definitive, but the combined evidence from (ii) — non-completers having lower, not higher, baseline PG — substantially reduces the plausibility of the most damaging MNAR scenarios.

**(iv) Why we retain completers-only as the primary analysis.** We have chosen to keep the completers-only analysis as the primary one in the main text, with MICE as a sensitivity analysis, for two reasons. First, the main inferences rest on within-person change-on-change regressions, which require non-missing PG and predictor scores at both W1 and W3 by construction — these participants are exactly the completers’ set. Second, the close agreement between completers-only and MICE estimates means the primary analysis is unlikely to be biased in a way that imputation would correct. The new Tables S5–S6 and the MICE figure are now cited in the Methods (data analysis section) and in the Limitations, where we explicitly flag the residual MNAR caveat.

**Table S3. Completers vs non-completers - continuous variables.**

| **variable** | **n_completers** | **n_dropouts** | **mean_compl** | **mean_dropout** | **sd_compl** | **sd_dropout** | **mean_diff** | **cohen_d** | **t_p** | **wilcox_p** |
| --- | --- | --- | --- | --- | --- | --- | --- | --- | --- | --- |
| PG | 2213 | 626 | 1.87 | 1.72 | 1.87 | 1.85 | 0.15 | 0.08 | 0.082 | 0.036 |
| VG Hours | 2213 | 821 | 19.74 | 22.64 | 25.13 | 27.36 | -2.90 | -0.11 | 0.008 | 0.090 |
| Impuls. | 2018 | 629 | 2.25 | 2.34 | 0.45 | 0.40 | -0.09 | -0.20 | 0.000 | 0.000 |
| Fam. Env. | 2082 | 696 | 3.27 | 3.16 | 0.58 | 0.59 | 0.11 | 0.19 | 0.000 | 0.000 |
| Age | 2094 | 719 | 10.95 | 11.97 | 2.00 | 2.04 | -1.02 | -0.51 | 0.000 | 0.000 |
| Mother Education | 2058 | 709 | 6.58 | 5.95 | 2.11 | 2.38 | 0.62 | 0.29 | 0.000 | 0.000 |
| Father Education | 2026 | 696 | 6.77 | 6.17 | 1.94 | 2.30 | 0.59 | 0.29 | 0.000 | 0.000 |

**Table S4. Completers vs non-completers - categorical variables**

| **variable** | **chi_sq** | **df** | **p_value** | **cramer_v** |
| --- | --- | --- | --- | --- |
| **group** | 33.17 | 3 | 0.000 | 0.10 |
| **sex** | 2.35 | 1 | 0.125 | 0.03 |
| **race** | 4.12 | 4 | 0.390 | 0.04 |
| **education** | 225.52 | 3 | 0.000 | 0.27 |
| **school** | 365.68 | 11 | 0.000 | 0.33 |

**Figure S2. Beta-coefficients of a regression analysis predicting changes in PG from W1 to W3 from changes in VG hours, impulsiveness and family environment over the same period (W1 to W3), separately for the completers only (as in the main manuscript) and after multivariate imputation with chained equations (MICE) using m = 20. As can be seen in the figure below, the coefficients are very similar. The only notable difference concerns low gamers, where the impact of VG hours and family environment on PG is numerically larger after data imputation (see figure below - MI (m=20)).**

These additional analyses and tables are now reported in the methods section, lines 284-289:

*“To reduce possible bias due to differential attrition between groups, our main analyses focused on completers, i.e., participants who had data at wave 1 and 3, and no missing-data imputation technique was applied. A comparison of completers and non-completers on baseline measures, as well as an analysis with multiple data imputation technique, indicated the same patterns as in the main analyses (see SI section 2 - Table S3, Table S4 and Figure S2).”*

Detailed results are available in the SI, Section 1.1, lines 35-42:

*“To probe the plausibility of MNAR, we compared completers (W1 and W3) and non-completers (W1 only) on all baseline variables (Tables S3 continuous, S4 categorical). Compared to completers, non-completers reported higher baseline VG hours, higher impulsiveness, slightly more positive family environment, were older, and had lower parental education; importantly, non-completers reported lower baseline PG, not higher. This pattern is the opposite of the worst-case MNAR scenario (in which drop-out is concentrated among adolescents with worsening PG), and suggests that the attrition pattern observed would tend to under- rather than over-estimate the longitudinal VG-hours→PG and impulsivity→PG associations in the completer subsample, which is consistent with our finding that these are the strongest effects.”*

*M*inor Concerns
P-values throughout Table 3 are reported as "0.00," which is not a valid p-value. Values should be reported to three decimal places, or as "< .001" where appropriate. This is a basic statistical reporting standard and should be corrected throughout.

**Response:** P-values have been corrected to 3 decimals and replaced with p < 0.001 where appropriate.

The supplementary materials note that this dataset has been used in numerous prior publications. The authors should provide a clearer account of what the present analysis adds that has not been reported elsewhere, beyond the novel stratification by baseline gaming group.

**Response:** This is mentioned in the SI, under section 6. 6. Published papers with this dataset (chronological order).

The data were collected in Singapore between 2007 and 2009. The gaming landscape has changed substantially since then, particularly in the prevalence of mobile gaming, online multiplayer formats, and free-to-play monetization models. The authors should be explicit about the limitations of extrapolating findings from this historical cohort to contemporary populations.

**Response:** We have clarified in the abstract that the *data was collected in 2007-2009* and added a sentence in the discussion lines 706-710: *“Finally, we recognize the gaming landscape has changed substantially since our data set was collected, particularly in the prevalence of mobile gaming, online multiplayer formats, and free-to-play monetization models. The findings from this historical cohort should thus be extrapolated to contemporary gaming with caution.”*

Reference 46 (cited to justify effect size thresholds) is listed as a Google search URL, which is not a citable source. The authors should cite the primary source (Field, 2013).

**Response:** The reference has been updated. The correct reference is

57.	Field A. Discovering statistics using IBM SPSS statistics: and sex and drugs and rock “n” roll. 4th edition. Los Angeles London New Delhi Singapore Washington DC: Sage; 2013. 915p.

Overall, the study raises an interesting hypothesis about moderating effects of baseline gaming habits and uses appropriate analytic techniques. However, the concerns above, particularly around the secondary analysis transparency, gaming time measurement validity, instrument age and construct alignment, and handling of attrition, are substantial enough that major revisions are warranted.

**Response:** We thank the reviewer for his positive feedback on our study and for his comments which have improved the manuscript!

7. PLOS authors have the option to publish the peer review history of their article ([what does this mean?](https://track.editorialmanager.com/CL0/https:%2F%2Fjournals.plos.org%2Fplosone%2Fs%2Feditorial-and-peer-review-process%23loc-peer-review-history/1/010f019d89888771-c8b5baf5-f1c4-40a5-b15f-5a4f5c9ea94e-000000/sUzoLHN5qm2QxPeb6ENJ1d4tK-ZhBzE3kmyNRu-Yh-Y=258)). If published, this will include your full peer review and any attached files.

If you choose “no”, your identity will remain anonymous but your review may still be made public.

**Do you want your identity to be public for this peer review?** For information about this choice, including consent withdrawal, please see our [Privacy Policy](https://track.editorialmanager.com/CL0/https:%2F%2Fwww.plos.org%2Fprivacy-policy/1/010f019d89888771-c8b5baf5-f1c4-40a5-b15f-5a4f5c9ea94e-000000/qV1bd_LnAn_oAxRn9lSb54zHeGUtqC5Y7p147ylMZR4=258).

Reviewer #1: No

Reviewer #3: **Yes:** Matthew W. R. Stevens

[NOTE: If reviewer comments were submitted as an attachment file, they will be attached to this email and accessible via the submission site. Please log into your account, locate the manuscript record, and check for the action link "View Attachments". If this link does not appear, there are no attachment files.]

To ensure your figures meet our technical requirements, please review our figure guidelines: <https://journals.plos.org/plosone/s/figures>

You may also use PLOS’s free figure tool, NAAS, to help you prepare publication quality figures: <https://journals.plos.org/plosone/s/figures#loc-tools-for-figure-preparation>.

NAAS will assess whether your figures meet our technical requirements by comparing each figure against our figure specifications.

*In compliance with data protection regulations, you may request that we remove your personal registration details at any time.* [*(Remove my information/details)*](https://track.editorialmanager.com/CL0/https:%2F%2Fwww.editorialmanager.com%2Fpone%2Flogin.asp%3Fa=r/1/010f019d89888771-c8b5baf5-f1c4-40a5-b15f-5a4f5c9ea94e-000000/7shpzsuomVXGTszX0TwcCP5-dBf9bAdgGat2zrmdhUc=258)*. Please contact the publication office if you have any questions.*
