# Supplementary material for: Problematic gaming in occasional, low, intermediate or heavy use gamers – not all screen time is created equal – A reanalysis
Source: PLoS One. 2026 Oct 21;21(10):e0356080. doi: 10.1371/journal.pone.0356080 (PMC13686491; doi:10.1371/journal.pone.0356080)
Supplement: Supplementary file 6 [file pone.0356080.s014.docx]

Dear Dr. Potenza,

On behalf of my co-authors, I am pleased to submit the revised version of our manuscript entitled **“Problematic gaming in occasional, low, intermediate or heavy gamers – not all screen time is created equal: a reanalysis”** (Manuscript ID: **PONE-D-25-39575R2**) for further consideration for publication in *PLOS ONE*.

We sincerely thank you and the reviewers for your careful evaluation of our manuscript and for the constructive comments provided. We are particularly encouraged that Reviewer #3 considered all previous concerns to have been adequately addressed.

In response to Reviewer #4’s additional suggestions, we have carefully revised the manuscript throughout. The revisions include clarification of terminology, improved reporting of the methodology and statistical analyses, additional procedural details, clarification of the gaming-engagement group definitions, refinement of several sections of the Introduction and Discussion, correction of typographical and language issues, and the addition of a brief Conclusion section. We have also expanded the discussion of the study’s limitations and improved the description of the problematic gaming measure.

A detailed, point-by-point response to every comment is provided in the accompanying **Response to Reviewers** document. For ease of review, we have also submitted both a tracked-changes version of the manuscript and a clean version.

We believe that these revisions have strengthened the manuscript considerably and hope that it is now suitable for publication in *PLOS ONE*.

Thank you for your time and consideration. We appreciate the opportunity to revise our work and look forward to your decision.

Sincerely,

Benoît Bediou, PhD
on behalf of all authors

Reviewer #4: Thank you for the opportunity to review this interesting manuscript. This study examined how differences in gaming engagement, impulsivity, and family environment relate to changes in problematic-gaming symptoms in adolescents with varying baseline levels of gaming engagement (i.e., duration of play). Please see my suggestions for the manuscript below:

**Abstract:**

- Please introduce the “VG” abbreviation when video games are first mentioned.

**Response:** We now introduce VG in the abstract

- Please include a space between “heavygamers.” In relevance to this, I would also consider using more person-centered language throughout the manuscript (including figures and tables). For example, “heavy gamers” may be replaced with “heavy gaming” or “heavy-gaming group.”

**Response:** Thank you — the missing space has been added. On person-centered language, we agree with the spirit of the suggestion and now explicitly introduce the four groups as gaming-engagement categories defined by baseline weekly play (not clinical categories or stable identities), using person-centered phrasing (e.g., “adolescents in the heavy-gaming group”) where it reads naturally. For readability and consistency across the numerous figures and tables — including all figures in the Supplementary Information — we have retained the concise group labels (occasional-, low-, intermediate-, heavy-gamers) throughout, now to be understood as shorthand for these engagement-based groups; this convention is stated explicitly where the groups are first defined.

**Introduction:**

- Please consider revising the first sentence of the introduction to reflect that it was Internet Gaming Disorder (not the broader construct of problematic gaming) that was included in Section III as a condition for further study.

**Response:** We have replaced PG by IGD in the first sentence.

- It may be helpful to include in-text citations in the “host” and “environment” sections (e.g., studies showing relationships between depression and problematic gaming).

**Response:** We have added in-text citations to the host and environment sentences. For the environment, we now cite Schneider, King & Delfabbro (2017) and Coşa et al. (2023) directly at the relevant sentence; for the host, we cite the existing references on psychopathology and ADHD/impulsivity (e.g., Gao et al., 2022; Koncz et al., 2023) at the point where depression, anxiety, attention problems, and impulsivity are introduced.

- I would recommend removing the word “established” in the subheading.

**Response:** Done — “established” has been removed from the subheading (now “Modifiable risk and protective factors of PG”).

- Replacing “predicting” with “associated with” may be preferable in the following sentence: “…predicting subsequent symptoms severity is strong.”

**Response:** Done — the sentence now reads “…associated with subsequent symptom severity is strong.”

- I believe the word “and” is missing here: “…perseverance, sensation seeking.”

**Response:** Done — corrected to “…perseverance, and sensation seeking.”

- Rewriting for clarity and adding in-text citations here may strengthen the sentence: “impulsivity is associated with difficulties considering alternative solutions, with acting precipitously and ignoring consequences and is recognized as a predictive factor in the development of maladaptive behaviors resulting from poor regulation of positive and negative emotions.”

**Response:** We have rewritten this sentence for clarity and added citations. It now reads: “Within the UPPS-P framework (Cyders & Smith, 2008), impulsivity reflects a tendency to act precipitously, to overlook alternative courses of action, and to disregard consequences. As such, it is a recognized predictor of maladaptive behaviors arising from poor regulation of positive and negative emotions (Sharma, Markon & Clark, 2014).”

- For rows 110-113, please consider clarifying that this is in the context of problematic gaming.

**Response:** We have clarified that this passage refers specifically to the context of problematic gaming.

- I would recommend proofreading the manuscript. For example, there appear to be repeated words in row 172. Also, some words appear to be capitalized, such as impulsiveness and family environment in row 198.

**Response:** We have carefully proofread the manuscript, removed the repeated word(s) (row 172), and corrected the erroneous capitalization of “impulsiveness” and “family environment” (row 198).

- Considering that the presentations of results (in the results section of the manuscript) are stratified according to group, it would be important to specify how many hours of gaming were considered to be occasional for the “occasional” gaming group both in the introduction and methods. Currently, in the introduction, it states “not usually playing,” but it would be important to report a specific number here or clarify how this was determined (it appears this information can be found in the results section, rows 406-208, but it may be helpful to provide this clarification earlier).

**Response:** We now provide the operational definition earlier, in both the Introduction and the Methods. “Occasional gamers” are participants who reported not usually playing video games at baseline (i.e., answering “no” when asked whether they usually play), and we cross-reference the quantile-based thresholds used for the other groups (low: 1–10 h/week; intermediate: 10–28 h/week; heavy: >28 h/week, mean ≈ 57 h/week).

**Methods:**

- Please consider changing 3’434 to 3,434

**Response:** Done — changed to 3,434.

- It may be helpful to include some additional information regarding the procedure (e.g., was the survey delivered on laptops/phones or on paper? Were participants reimbursed? How exactly were the schools/students recruited?).

**Response:** We have added detail to the procedure and now cite Gentile et al. (2011) and Choo et al. (2010), where the full data-collection procedures are described. The survey was administered as a paper-and-pencil questionnaire in participants’ classrooms by their teachers, with four counterbalanced versions distributed across classes. Schools were recruited as a convenience sample: principals of twelve schools (six primary and six secondary) accepted an invitation to participate, and the classes were selected by each school. Participation was voluntary and unpaid (no reimbursement was provided). We now state this in the Procedure and cite Gentile et al. (2011) and Choo et al. (2010) for the full data-collection procedures.

- The wording around “most” may be slightly confusing for readers in rows 232-233. Please consider clarifying the “most of the cohort was followed” section. If it is referring to participants who were retained over the three-year follow-up period, please explicitly state this.

**Response:** We have clarified this. The sentence now states explicitly that this refers to participants who were retained across the three-year follow-up period (three annual waves).

- I suggest being more cautious with the wording in rows 243-245, as some DSM-IV pathological-gambling criteria (e.g., chasing losses, committing illegal acts, and relying on others for financial bailouts) were not transferred to IGD.

**Response:** We agree and have made the wording more cautious. We now note that not all DSM-IV pathological-gambling criteria were transferred to IGD: only nine of the ten items map onto the DSM-5 IGD criteria, and criteria such as chasing losses, committing illegal acts, and financial bailout are DSM-IV-specific and are not represented in our adapted instrument (see Measures and Table S6).

- I would also recommend slightly adjusting the wording for rows 242-243 (perhaps removing the “10-item” part) to better align with the following sentence in the referenced King et al. article: “Prior to the provisional DSM-5 criteria for GD, researchers would often adapt the criteria of other disorders (e.g., pathological gambling in the DSM-IV-TR).”

**Response:** We have adjusted the wording to align with King et al. We removed the “10-item” descriptor where it created ambiguity and now describe the instrument as adapted from the DSM-IV pathological-gambling criteria, consistent with King et al.’s phrasing.

- Please include a space between “and13%”

**Response:** Done — a space has been added (“and 13%”).

- After the “measures included in this reanalysis” subheading, it would be helpful to include additional subheadings for the other sections (e.g., an “ethics” subheading for rows 306 -317 and also a “statistical analysis” subheading).

**Response:** We have added the suggested subheadings: an “Ethics” subheading (former rows 306–317) and a “Statistical analysis” subheading.

- Please correct the typo for “environement”

**Response:** Done — corrected to “environment” throughout.

- Please change “ensures” to “ensure” in row 324.

**Response:** Done — changed “ensures” to “ensure.”

- Some additional specific information would be helpful for the statistical analysis section. For example, what statistical package was used, how model fit and significance were evaluated, etc.

**Response:** We have expanded the Statistical analysis section. All analyses were conducted in R (Version 4.6.1). Specification-curve analysis was implemented with the specr package; partial η² effect sizes and Type-III ANOVAs were computed with the car, effectsize and heplots packages. Linear mixed-effects moderation models were fitted with lme4 (with afex, emmeans and interactions for follow-up tests). Finally, the CLPM and RI-CLPM were estimated with lavaan using MLR estimator with FIML for missing data. Statistical significance was evaluated at α = .05, and effect sizes were interpreted using the conventional benchmarks already reported in the manuscript. Model fits were estimated using AIC and BIC for ANOVAs and mixed-effects models as well as with CFI, RMSEA and SRMR for RI-CLPM models.

- Regarding the section describing the problematic gaming scale, it would be helpful to briefly summarize the 10 items, given that the results show “sneaky” and “deception” as separate symptoms.

**Response:** We now briefly summarize the ten items of the PG scale in the Measures section and list them in full in Table S2/S6, so that the symptom-level results (e.g., “sneaky” and “deception”) can be readily interpreted.

**Results:**

- It appears that the number of hours of gaming per week that determines what group a participant is in is different from what is stated in the introduction. Also, regarding the heavy-gaming group, it would be helpful to add the range of hours. For the remaining groups, it would be helpful to add the mean.

**Response:** We have reconciled the group definitions between the Introduction and the Results so that consistent thresholds are reported throughout. For the heavy-gaming group we now report the range (up to 106 h/week; mean ≈ 57 h/week), and we have added the mean for each remaining group (occasional, low, and intermediate).

**Discussion:**

- In the discussion or the introduction, it may be beneficial to provide a nuanced discussion of the limitations associated with categorizing participants into named gaming-engagement groups (“occasional, low, intermediate, or heavy”) solely on the basis of hours played.

**Response:** We have expanded this limitation (currently the third limitation) to discuss more explicitly that classifying participants into named engagement groups solely on the basis of self-reported hours is a simplification that does not capture motivation, functional impairment, or context, and that the heavy-gaming group in particular is likely heterogeneous.

- Regarding the fifth limitation, it may be helpful to consider discussing the use of the TimeLine FollowBack in previous gaming disorder studies (e.g., “A brief internet-delivered intervention for the reduction of gaming-related harm: A feasibility study”) to reduce the concerns stated.

**Response:** We have added a discussion of the Timeline Followback (TLFB) approach to the fifth limitation, noting that more structured retrospective methods such as TLFB have been used in gaming research (e.g., Park et al., 2020, Computers in Human Behavior Reports) and could mitigate the imprecision of typical-day self-reports of gaming time in future work.

- Please consider including a brief conclusion section (and subheading) at the end of the discussion.

**Response:** We have added a brief Conclusion section with its own subheading at the end of the Discussion, as follows:

Conclusion. By stratifying adolescents according to their baseline gaming habits, this study shows that the factors most strongly linked to changes in problematic gaming differ across engagement levels: changes in gaming time dominate in occasional gamers, whereas changes in impulse control dominate in heavy gamers. These findings argue against a one-size-fits-all approach and support tailoring prevention and intervention to a young person’s current gaming profile: supporting impulse regulation in heavy gamers and addressing the social integration of an emerging interest in gaming among occasional gamers. More broadly, they underscore that time spent playing is, on its own, an incomplete index of harm, and that future work combining engagement level with measures of motivation and function will be needed to refine these recommendations.
