# Supplementary material for: Problematic gaming in occasional, low, intermediate or heavy use gamers – not all screen time is created equal – A reanalysis
Source: PLoS One. 2026 Oct 21;21(10):e0356080. doi: 10.1371/journal.pone.0356080 (PMC13686491; doi:10.1371/journal.pone.0356080)
Supplement: Peer review history

# Peer review: Decision Letter 0

**DOI:** 10.1371/journal.pone.0356080.r001

**Authors:** Yusup Martyastiadi

17 Nov 2025

Internet Gaming Disorder in novel, low, intermediate or heavy gamers – not all screen time is created equal

PLOS ONE

Dear Dr. Bediou,

Thank you for submitting your manuscript to PLOS ONE. After careful consideration, we feel that it has merit but does not fully meet PLOS ONE’s publication criteria as it currently stands. Therefore, we invite you to submit a revised version of the manuscript that addresses the points raised during the review process.

Please submit your revised manuscript by Jan 01 2026 11:59PM. If you will need more time than this to complete your revisions, please reply to this message or contact the journal office at plosone@plos.org. When you're ready to submit your revision, log on to https://www.editorialmanager.com/pone/ and select the 'Submissions Needing Revision' folder to locate your manuscript file.

Please include the following items when submitting your revised manuscript:

- A rebuttal letter that responds to each point raised by the academic editor and reviewer(s). You should upload this letter as a separate file labeled 'Response to Reviewers'.
- A marked-up copy of your manuscript that highlights changes made to the original version. You should upload this as a separate file labeled 'Revised Manuscript with Track Changes'.
- An unmarked version of your revised paper without tracked changes. You should upload this as a separate file labeled 'Manuscript'.

If you would like to make changes to your financial disclosure, please include your updated statement in your cover letter. Guidelines for resubmitting your figure files are available below the reviewer comments at the end of this letter.

If applicable, we recommend that you deposit your laboratory protocols in protocols.io to enhance the reproducibility of your results. Protocols.io assigns your protocol its own identifier (DOI) so that it can be cited independently in the future. For instructions see: https://journals.plos.org/plosone/s/submission-guidelines#loc-laboratory-protocols. Additionally, PLOS ONE offers an option for publishing peer-reviewed Lab Protocol articles, which describe protocols hosted on protocols.io. Read more information on sharing protocols at https://plos.org/protocols?utm_medium=editorial-email&utm_source=authorletters&utm_campaign=protocols.

We look forward to receiving your revised manuscript.

Kind regards,

Yusup Sigit Martyastiadi, Dr.Sn.

Guest Editor

PLOS ONE

Journal Requirements:

When submitting your revision, we need you to address these additional requirements.

1.Please ensure that your manuscript meets PLOS ONE's style requirements, including those for file naming. The PLOS ONE style templates can be found at

https://journals.plos.org/plosone/s/file?id=wjVg/PLOSOne_formatting_sample_main_body.pdf and

https://journals.plos.org/plosone/s/file?id=ba62/PLOSOne_formatting_sample_title_authors_affiliations.pdf

2. Thank you for stating the following financial disclosure:

“This study is supported by the Swiss National Science Foundation  100014_212405 grant to BB, the European Research Council Synergy grant (BrainPlay, 810580) to DB, the Singapore Ministry of Education, and the Media Development Authority (Project #EPI/06AK),”

Please state what role the funders took in the study.  If the funders had no role, please state: "The funders had no role in study design, data collection and analysis, decision to publish, or preparation of the manuscript."

If this statement is not correct you must amend it as needed.

Please include this amended Role of Funder statement in your cover letter; we will change the online submission form on your behalf.

3. In the online submission form you indicate that your data is not available for proprietary reasons and have provided a contact point for accessing this data. Please note that your current contact point is a co-author on this manuscript. According to our Data Policy, the contact point must not be an author on the manuscript and must be an institutional contact, ideally not an individual. Please revise your data statement to a non-author institutional point of contact, such as a data access or ethics committee, and send this to us via return email. Please also include contact information for the third party organization, and please include the full citation of where the data can be found.

4. If the reviewer comments include a recommendation to cite specific previously published works, please review and evaluate these publications to determine whether they are relevant and should be cited. There is no requirement to cite these works unless the editor has indicated otherwise.

Additional Editor Comments:

Dear authors,

Your submission, "Internet Gaming Disorder in novel, low, intermediate or heavy gamers – not all screen time is created equal" has been reviewed. We encourage you to submit a revised version that carefully addresses all reviewers’ comments. Please note that failure to adequately address the concerns raised may result in the rejection of your submission. An editor will evaluate the revisions and determine whether they meet the necessary criteria for publication.

The reviewers' comments are included at the bottom of this email. Please respond to each point in the reviewers' comments and identify what changes you have made. If you find any of the reviewer's comments to be unjustified or inappropriate, please explain your perspective.

[Note: HTML markup is below. Please do not edit.]

Reviewers' comments:

Reviewer's Responses to Questions

**Comments to the Author**

1. Is the manuscript technically sound, and do the data support the conclusions?

Reviewer #1: Partly

Reviewer #2: Yes

**********

2. Has the statistical analysis been performed appropriately and rigorously?

Reviewer #1: Yes

Reviewer #2: Yes

**********

3. Have the authors made all data underlying the findings in their manuscript fully available?

The PLOS Data policy

Reviewer #1: Yes

Reviewer #2: No

**********

4. Is the manuscript presented in an intelligible fashion and written in standard English?

Reviewer #1: Yes

Reviewer #2: Yes

**********

Reviewer #1: In this paper the authors explore three factors – impulsiveness, gaming time and family environment – as risk and resilience factors for internet Gaming Disorder (IGD) across a two year longitudinal study in children. Gaming time and impulsiveness were found to be risk factors for IGD symptoms whereas a better family environment was protective.

This paper had some interesting findings but had a substantial number of issues as well.

Introduction

Lines 52-57. It isn’t clear what was studied longitudinally. Was it persistence of IGD symptoms? Was it treatment?

Lines 57-58. Also, it is not true that impulsivity and game time are the two strongest predictors.

At r=.11, impulsivity was a weaker predictor than self control (r=-.27) and parent child relationship (r=-.15). If self control and impulsivity have differing effect sizes then they are different constructs and should not be treated interchangeably.

In terms of game time, the authors note in lines 84-93 that the correlation between gaming time and IGD is generally small, and this is my experience. This undermines the contention that game time is the strongest predictor of IGD.

Overall, the second paragraph could be rewritten for clarity and focus.

Paragraph beginning line 94

This paper seems to be about predictors of IGD rather than outcomes, and this paragraph on the impact of IGD on mental health seems to come from nowhere and not be linked to the central tenets of the paper. If this paper is centrally interested in outcomes as well as predictors then the introduction needs to be more clearly framed with the aims of the paper stated clearly up front.

Lines 136-138. I do not agree that “the most common modifiable risk and protective factors for IGD identified in past research” are “gaming time, impulsiveness, and family environment”. There are many risk and protective factors for IGD (for example, see the Warburton et al 2022 risk and resilience paper on IGD with a large range of factors, Li et al 2023 with risk and protective factors related to GD, Jeong et al 2020 on several factors associated with IGD persistence plus others; there are several papers on risk and protective factors for problematic social media use that may also be relevant). Many such factors are modifiable. I think it is important that all key risk and protective factors be acknowledged in the introduction, with a compelling reason then given as to why the factors of interest in this study were singled out.

Lines 145-157. Discusses results rather than hypotheses. I will deal with the age of the data elsewhere.

Overall, the introduction lacked clarity and focus and for me did not provide a compelling argument for the methodology of the study conducted.

Method

The single biggest issue with this paper is the age of the data (2007-2009), all of which predates by several years the proposed diagnostic criteria for IGD (2013). Apart from the issue of publishing data now nearly 20 years old, how can a person meaningfully study data purported to measure a phenomenon that was not created, reported or measured until several years afterward? At the very least this study must be reframed as being about problematic gaming rather than as being related to a disorder not conceptualised until after the data were collected.

It is not sufficient to say that the measures and methodology are reported elsewhere. This paper must stand alone in terms of describing the sample and measures used here. Given the contentiousness of claiming IGD was measured (see below) it is crucial that all measures be made clear and described in sufficient detail. It is also important that key sample characteristics are reported.

It is also purported that the study measured IGD. This cannot be factually true given that no such diagnosis existed at the time of the study.

Results

Should decimals be to two decimal points (e.g., Table 2)?

I think it is important to note that the IGD score is [presumably – we never see the measure] a score of IGD symptomology providing a continuous numeric measure on a continuum, rather than an indication of actually having a gaming disorder. So this is not really a study of IGD or any sort of disorder in a way, it is a study of the level of symptoms, with low levels at one end and higher levels at the other, with no way of knowing which, if any, participants had a gaming disorder. In line 335 diagnosis of IGD is discussed in relation to the data. Again, these data predate the diagnosis or any measure of IGD and the data are continuous without categorising IGD.

Paragraph starting line 248. It is not simply the case that novel gamers had some IGD symptoms at wave 1 – their levels were nearly as high as at wave 3 and nearly as high as all other groups. Indeed higher than the ‘low’ group. This does not seem to be entirely an artefact of the wording or timing of presentation of measures – for me it is a significant anomaly that casts doubt on the measure of IGD and all the IGD data.

Overall I found the results hard to follow and interpret, but having said that do think the findings are interesting and publishable.

Discussion

Line 365. Is ‘remarkable’ the best word for a finding that is strongly in line with past research? Maybe noteworthy ….

I am not sure one can categorise IGD as an impulse control problem (line 383). Rather, impulsiveness is one risk factor. It is certainly not an adequate description of the disorder.

Overall I found the discussion to be lacking in depth, entirely omitting the clear limitations of the paper or adequately situating findings in existing literature.

Minor issues

There is often not a gap between a word and a following reference number in brackets.

In Summary

This paper has a number of serious issues – the age of the data, the measure of IGD predating the existence of the diagnosis, the lack of detail about key parts of the study, and a lack of clarity and focus across the paper. I do think the results are interesting but would need to be presented in a heavily redrafted paper with a completely new focus (not IGD).

Reviewer #2: Major Issues Requiring Revision:

1. Data Availability (Critical)

Does not meet PLOS ONE standards

Must provide deidentified data to controlled-access repository or substantially enhance supplementary materials with complete analysis code and results

2. Analytical Approach

Recommendation: Replace or supplement difference score analyses with:

Cross-lagged panel models to establish temporal precedence

Growth curve models to capture trajectories

These approaches handle missing data better and test directional hypotheses more rigorously

Current claim (lines 269-271): "we can not only determine the direction of influence" overstates what difference scores can establish

3. Missing Data & Attrition

Required: Add section describing:

Pattern of missingness across waves

Comparison of completers vs. non-completers on key variables

Sensitivity analyses (if feasible)

Concern: Differential attrition by gaming group could bias results

4. Grouping Strategy

Consider: Justify quantile-based grouping or explore:

Theory-driven cutoffs (e.g., APA's 30 hours/week mentioned line 245)

Data-driven approaches (latent profile analysis)

Sensitivity analyses with different grouping schemes

5. Novel-Gamers Definition

Lines 249-259 reveal measurement timing issues

Required clarification: Were questions about "usual" gaming and "past year" IGD symptoms administered in the same session or different sessions?

This affects interpretation of results for novel-gamers substantially

Moderate Issues:

6. Effect Size Interpretation

While the stratified findings are interesting, the practical significance needs discussion

Even in novel-gamers, VG hours explain only 12% of IGD variance

Add: Discussion of what these effect sizes mean for intervention design

7. Exploratory Analyses (Figure 3)

Apply FDR correction for multiple comparisons

Be more cautious about interpreting "sneaky" and "deception" symptoms (lines 342-351)

These are interesting hypotheses but need replication

8. Temporal Ordering

Strengthen claims: The phrase "changes in VG consumption influenced changes in IGD" (lines 26-27) implies causation that difference scores cannot establish

Suggest rewording throughout to reflect associations rather than causal effects

9. Alternative Explanations

The discussion could address:

Why does impulsivity matter more for heavy gamers? (addressed briefly)

Could reverse causation explain some findings?

Role of game genres, social gaming, etc.

Minor Issues:

10. Methods Detail

SI section mentions IGD, VG consumption, impulsivity measures but main text should briefly describe:

How many items in each scale?

Response formats?

Reliability estimates?

11. Table 1

Add column showing attrition rates by group

Consider adding baseline comparisons of groups on outcome measures

12. Figure 1

Very informative but consider adding:

Sample sizes at each timepoint

Statistical tests of mean changes

13. Supplementary Materials

Consider moving Table S1 to main text - these are primary results

Provide complete correlation matrices for all variables

Strengths to Highlight:

Novel stratification approach addresses real heterogeneity in the literature

Large, longitudinal sample

Sophisticated control for confounds via SCA

Practical implications for tailored interventions

Transparent acknowledgment of exploratory analyses

Suggested Additional Analyses:

Moderation analyses: Formally test whether gaming group moderates VG hours→IGD and impulsivity→IGD relationships

Dose-response: Within each group, is there a linear relationship between change in VG hours and change in IGD?

Bidirectional effects: Even with difference scores, test whether baseline IGD predicts change in VG hours

Discussion Enhancements:

Compare effect sizes to other longitudinal IGD studies

Discuss clinical vs. statistical significance more explicitly

Address limitations more comprehensively (single cultural context, self-report measures, attrition)

Clarify intervention implications: How would one "introduce gaming progressively" (line 31)?

**********

what does this mean?). If published, this will include your full peer review and any attached files.

If you choose “no”, your identity will remain anonymous but your review may still be made public.

**Do you want your identity to be public for this peer review?**  For information about this choice, including consent withdrawal, please see our Privacy Policy

Reviewer #1: No

Reviewer #2: No

**********

[NOTE: If reviewer comments were submitted as an attachment file, they will be attached to this email and accessible via the submission site. Please log into your account, locate the manuscript record, and check for the action link "View Attachments". If this link does not appear, there are no attachment files.]

To ensure your figures meet our technical requirements, please review our figure guidelines: https://journals.plos.org/plosone/s/figures

You may also use PLOS’s free figure tool, NAAS, to help you prepare publication quality figures: https://journals.plos.org/plosone/s/figures#loc-tools-for-figure-preparation.

NAAS will assess whether your figures meet our technical requirements by comparing each figure against our figure specifications.

# Author comment: Author response to Decision Letter 1

**DOI:** 10.1371/journal.pone.0356080.r002

21 Jan 2026

1. funding

We added the following statement in the data availability section: "The funders provided only financial support and had no involvement in the research's study design, data collection, analysis, writing, or decision to publish."

2. Data availability

In the online submission form you indicate that your data is not available for proprietary reasons and have provided a contact point for accessing this data. Please note that your current contact point is a co-author on this manuscript. According to our Data Policy, the contact point must not be an author on the manuscript and must be an institutional contact, ideally not an individual. Please revise your data statement to a non-author institutional point of contact, such as a data access or ethics committee, and send this to us via return email. Please also include contact information for the third party organization, and please include the full citation of where the data can be found.

We have updated the contact information for data availability. The institutional contact for data sharing is the Research Support Office at Nanyang Technological University. Research Support Office (RSO), NTU Singapore Dir-RSO@ntu.edu.sg

3. References

If the reviewer comments include a recommendation to cite specific previously published works, please review and evaluate these publications to determine whether they are relevant and should be cited. There is no requirement to cite these works unless the editor has indicated otherwise.

All references suggested by reviewers have been added where appropriate.

Additional Editor Comments:

Dear authors,

Your submission, "Internet Gaming Disorder in novel, low, intermediate or heavy gamers – not all screen time is created equal" has been reviewed. We encourage you to submit a revised version that carefully addresses all reviewers’ comments. Please note that failure to adequately address the concerns raised may result in the rejection of your submission. An editor will evaluate the revisions and determine whether they meet the necessary criteria for publication.

The reviewers' comments are included at the bottom of this email. Please respond to each point in the reviewers' comments and identify what changes you have made. If you find any of the reviewer's comments to be unjustified or inappropriate, please explain your perspective.

[Note: HTML markup is below. Please do not edit.]

Reviewers' comments:

Reviewer's Responses to Questions

Comments to the Author

1. Is the manuscript technically sound, and do the data support the conclusions?

The manuscript must describe a technically sound piece of scientific research with data that supports the conclusions. Experiments must have been conducted rigorously, with appropriate controls, replication, and sample sizes. The conclusions must be drawn appropriately based on the data presented.

Reviewer #1: Partly

Reviewer #2: Yes

2. Has the statistical analysis been performed appropriately and rigorously?

Reviewer #1: Yes

Reviewer #2: Yes

3. Have the authors made all data underlying the findings in their manuscript fully available?

The PLOS Data policy requires authors to make all data underlying the findings described in their manuscript fully available without restriction, with rare exception (please refer to the Data Availability Statement in the manuscript PDF file). The data should be provided as part of the manuscript or its supporting information, or deposited to a public repository. For example, in addition to summary statistics, the data points behind means, medians and variance measures should be available. If there are restrictions on publicly sharing data—e.g. participant privacy or use of data from a third party—those must be specified.

Reviewer #1: Yes

Reviewer #2: No

4. Is the manuscript presented in an intelligible fashion and written in standard English?

PLOS ONE does not copyedit accepted manuscripts, so the language in submitted articles must be clear, correct, and unambiguous. Any typographical or grammatical errors should be corrected at revision, so please note any specific errors here.

Reviewer #1: Yes

Reviewer #2: Yes

4. Review Comments to the Author

Please use the space provided to explain your answers to the questions above. You may also include additional comments for the author, including concerns about dual publication, research ethics, or publication ethics. (Please upload your review as an attachment if it exceeds 20,000 characters)

Based on the comments from the reviewers, the introduction has been entirely rewritten, and the work now discusses problematic gaming and not IGD, a term coined after our problematic gaming questionnaire was administered (as correctly noted by Reviewer 1). As we detail below, our questionnaire indeed differs by one question as compared to those of IGD. The rewritten introduction is divided into 3 sections. We begin by reviewing the different modifiable risk and protecting factors found in previous research. These are now introduced following an epidemiological model that distinguishes between factors related to the agent (game factors), to the host (intrapersonal factors) and to the environment (social and interpersonal factors). In this context, we introduce the multidimensional model of impulsivity which links impulsivity to other related constructs (e.g., self-control, impulse regulation), as well as to presumably unrelated factors, such as maladaptive cognitions and dysregulated behaviors (e.g., rule-breaking) as proposed in the I-PACE model. The second section focuses on the heterogeneity of the association between gaming time and problematic gaming. In the third and last section, we introduce our study goal and hypotheses which are that (1) increases in VG consumption and impulsiveness will be positively associated with increases in PG symptoms, while improvements in the family environment will be protective; and (2) the predictive strength of these factors will vary significantly depending on the baseline gaming habit group, consistent with the hypothesized non-linear model. This rewriting clarifies the hypotheses tested and methodology of the study.

Reviewer #1: In this paper the authors explore three factors – impulsiveness, gaming time and family environment – as risk and resilience factors for internet Gaming Disorder (IGD) across a two year longitudinal study in children. Gaming time and impulsiveness were found to be risk factors for IGD symptoms whereas a better family environment was protective.

This paper had some interesting findings but had a substantial number of issues as well.

Introduction

Lines 52-57. It isn’t clear what was studied longitudinally. Was it persistence of IGD symptoms? Was it treatment?

The meta-analysis by Zhuang et al. focused on studies with a longitudinal design, whether including a treatment (intervention) or not (i.e. IGD persistence). This has been clarified in the text.

Lines 57-58. Also, it is not true that impulsivity and game time are the two strongest predictors. At r=.11, impulsivity was a weaker predictor than self control (r=-.27) and parent child relationship (r=-.15). If self control and impulsivity have differing effect sizes then they are different constructs and should not be treated interchangeably.

We appreciate the reviewer drawing our attention to the difference between impulsivity and self-control. We have clarified that self‑control (the positive side) refers to the capacity to generate adapted behavioral responses, often tested in the context of questionnaires presenting everyday life situations. Impulsivity (the down side) refers to the personality trait which can be measured either using “objective” task‑based measures or using subjective questionnaires such as the UPPS or the Barratt Impulsiveness Scale, as in the present work. To avoid confusion, we have revised the text to clarify that these constructs are distinct, and we also discuss how these two constructs interact.

In terms of game time, the authors note in lines 84-93 that the correlation between gaming time and IGD is generally small, and this is my experience. This undermines the contention that game time is the strongest predictor of IGD.

As noted by the reviewer, the meta‑analysis referred above and in later in the discussion (lines 84–93), the correlation between game time and problematic gaming is generally small. We have rewritten to properly characterize its predictive strength. Our point was rather to draw attention to the fact that game time remains one of the most consistently examined variables in the literature. We have rephrased accordingly.

Overall, the second paragraph could be rewritten for clarity and focus.

The second paragraph of the introduction (and in fact the whole introduction) has been substantially rewritten to increase clarity and focus, as well as to further acknowledge the existence of other modifiable risk and protective factors (see next comments from Reviewer 1).

Paragraph beginning line 94

This paper seems to be about predictors of IGD rather than outcomes, and this paragraph on the impact of IGD on mental health seems to come from nowhere and not be linked to the central tenets of the paper. If this paper is centrally interested in outcomes as well as predictors then the introduction needs to be more clearly framed with the aims of the paper stated clearly up front.

The reviewer is correct. We have removed the section on the relationship between gaming and other health consequences of problematic gaming as this is not directly relevant to the present study.

Lines 136-138. I do not agree that “the most common modifiable risk and protective factors for IGD identified in past research” are “gaming time, impulsiveness, and family environment”. There are many risk and protective factors for IGD (for example, see the Warburton et al 2022 risk and resilience paper on IGD with a large range of factors, Li et al 2023 with risk and protective factors related to GD, Jeong et al 2020 on several factors associated with IGD persistence plus others; there are several papers on risk and protective factors for problematic social media use that may also be relevant). Many such factors are modifiable. I think it is important that all key risk and protective factors be acknowledged in the introduction, with a compelling reason then given as to why the factors of interest in this study were singled out.

We thank the reviewer for pointing out these additional studies. We now provide an extended list of modifiable risk and protective factors in the introduction, including the references suggested by Reviewer 1 as well as new references to other relevant work. We also made explicit that the choice of factors of interest was guided by the epidemiological triad framework to cover factors associated with each level: the agent (gaming consumption), the host (impulsivity) and the environment (family environment).

Lines 145-157. Discusses results rather than hypotheses. I will deal with the age of the data elsewhere.

We have rephrased this section of the introduction to present our hypotheses rather than results.

Overall, the introduction lacked clarity and focus and for me did not provide a compelling argument for the methodology of the study conducted.

We thank Reviewer 1 for their constructive feedback. We have made substantial changes to improve the clarity and focus of the introduction as well as to better introduce our hypotheses and how they justify the methodology.

Method

The single biggest issue with this paper is the age of the data (2007-2009), all of which predates by several years the proposed diagnostic criteria for IGD (2013). Apart from the issue of publishing data now nearly 20 years old, how can a person meaningfully study data purported to measure a phenomenon that was not created, reported or measured until several years afterward? At the very least this study must be reframed as being about problematic gaming rather than as being related to a disorder not conceptualised until after the data were collected.

We acknowledge this anachronism and have now rewritten the manuscript to refer to problematic gaming (PG) rather than IGD. The questions use in the present study cover 9 of the 10 symptoms of the DSM-V criteria for IGD with the one divergence concerning the continuation question in the DSM-V which is absent and replaced by a question about negative consequences (sneaky behaviors) in the present study. This has been clarified by adding a column with the IGD items to Table S4 and updating the legend to mention the diverging item.

It is not sufficient to say that the measures and methodology are reported elsewhere. This paper must stand alone in terms of describing the sample and measures used here. Given the contentiousness of claiming IGD was measured (see below) it is crucial that all measures be made clear and described in sufficient detail. It is also important that key sample characteristics are reported.

We have moved the details about the sample and the measures from the supplementary information to the methods section of the main manuscript.

It is also purported that the study measured IGD. This cannot be factually true given that no such diagnosis existed at the time of the study.

This important point is now clarified in the introduction, where we note that we use the term problematic gaming in this work to cover PG, GD and IGD, as the DSM scales have changed.

Results

Should decimals be to two decimal points (e.g., Table 2)?

We have added the second decimals that were missing.

I think it is important to note that the IGD score is [presumably – we never see the measure] a score of IGD symptomology providing a continuous numeric measure on a continuum, rather than an indication of actually having a gaming disorder. So this is not really a study of IGD or any sort of disorder in a way, it is a study of the level of symptoms, with low levels at one end and higher levels at the other, with no way of knowing which, if any, participants had a gaming disorder. In line 335 diagnosis of IGD is discussed in relation to the data. Again, these data predate the diagnosis or any measure of IGD and the data are continuous without categorising IGD.

We have clarified that the dependent variable is the sum of all 10 items, which are available in Table S2, and represents a measure of the severity of PG symptomatology.

Paragraph starting line 248. It is not simply the case that novel gamers had some IGD symptoms at wave 1 – their levels were nearly as high as at wave 3 and nearly as high as all other groups. Indeed higher than the ‘low’ group. This does not seem to be entirely an artefact of the wording or timing of presentation of measures – for me it is a significant anomaly that casts doubt on the measure of IGD and all the IGD data.

We thank the reviewer for raising this important point. It should not come as a surprise that, amongst novel gamers, there are children who are not allowed to play video game at home and yet may engage in video game play outside of the family setting. This disconnect is not surprising and in fact aligned with the most frequently reported “symptoms” at baseline in novel gamers being, from most frequent to least frequent: items 9 (loss of control / unsuccessful attempts), followed by item 6 (escape), item 2 (withdrawal), item 7 (loss of interests), and item 1 (preoccupation).

This has now been addressed lines 360-370 of the revised MS.

Overall I found the results hard to follow and interpret, but having said that do think the findings are interesting and publishable.

We thank the reviewer and have rephrased the results and discussion to make them easier to follow.

Discussion

Line 365. Is ‘remarkable’ the best word for a finding that is strongly in line with past research? Maybe noteworthy ….

We have replaced remarkable with noteworthy

I am not sure one can categorise IGD as an impulse control problem (line 383). Rather, impulsiveness is one risk factor. It is certainly not an adequate description of the disorder.

We thank the reviewer for this clarification. We have removed this sentence and rephrased part of our text. We agree with the reviewer that it does not provide direct evidence for considering PG as an impulse control problem.

Overall I found the discussion to be lacking in depth, entirely omitting the clear limitations of the paper or adequately situating findings in existing literature.

The discussion now better situates our findings within the existing literature. We have also added a limitation section. In particular, we now emphasize that the direction and magnitude of the observed effects is consistent with the prior work discussed in the introduction. We also acknowledge the inherent limitations of our measures of family environment and of impulsivity, especially when it comes to capturing more complex dynamics associated with the constructs of social environment and of impulse regulation.

Minor issues

There is often not a gap between a word and a following reference number in brackets.

Missing spaces have been added.

In Summary

This paper has a number of serious issues – the age of the data, the measure of IGD predating the existence of the diagnosis, the lack of detail about key parts of the study, and a lack of clarity and focus across the paper. I do think the results are interesting but would need to be presented in a heavily redrafted paper with a completely new focus (not IGD).

We thank the reviewer for their thoughtful and constructive feedback on our manuscript. We hope our interesting results are now better contextualized thanks to the novel introduction, the clarification about the collected measures, the amount of missing data and attrition rates across waves and measures, as well as the updated discussion. We thank the reviewer for their suggestions as to how to strengthen our paper.

Reviewer #2: Major Issues Requiring Revision:

1. Data Availability (Critical)

Does not meet PLOS ONE standards

Must provide deidentified data to controlled-access repository or substantially enhance supplementary materials with complete analysis code and results

We have updated the contact information for data availability. The institutional contact for data sharing is the Research Support Office at Nanyang Technological University. Research Support Office (RSO), NTU Singapore Dir-RSO@ntu.edu.sg

2. Analytical Approach

Recommendation: Replace or supplement difference score analyses with:

Cross-lagged panel models to establish temporal precedence.

Growth curve models to capture trajectories

These approaches handle missing data better and test directional hypotheses more rigorously

We thank the reviewer for these thoughtful suggestions. Our use of Specification Curve Analysis (SCA) of change scores (including autoregressive effects) was specifically chosen to address potential p-hacking and model uncertainty inherent in longitudinal observational data analysis, particularly concerning the selection of covariates. While CLPMs and RI-CLPMs are excellent for isolating within-person dynamics, the SCA framework offers superior robustness against specification choices.

Yet, because the SCA doesn’t assess the directionality of the associations, we have now complemented the main (SCA) analysis with cross-lagged panel models (CLPM) which provide better tests of directionality of reciprocal influences while controlling for auto-regressive effects. In addition, we also conducted random-intercept cross-lagged panel models (RI-CLPM) which better separate within-person from between-person effects. These analyses were performed at the whole group level as their goal is to verify the directionality of influences rather than replicate the effects observed in the main analysis. The results are summarized in supplementary Table S3 and supplementary Figure S6.

CLPM analyses revealed no significant influences at the between-person level. Yet, at the within-subject level, RI-CLPM models suggested a unidirectional (positive) influence of PG on impulsiveness (more PG symptoms at time t leading to higher levels of impulsiveness at time t + 1) and bidirectional (negative) influences between family environment and PG. Of note, no relationship was found between gaming time and PG whether at the between-subject or at the within-subject level. This new analysis is now noted in the Introduction, lines 286-293. The results are then summarized in the results section, lines 482-496.

In addition, we now also discuss these new results lines 559-574 of the revised MS and highlight the strength of our multi-method approach as requested by Reviewer 2.

Current claim (lines 269-271): "we can not only determine the direction of influence" overstates what difference scores can establish

This section has been reworded.

3. Missing Data & Attrition

Required: Add section describing:

Pattern of missingness across waves.

Comparison of completers vs. non-completers on key variables

Sensitivity analyses (if feasible)

Concern: Differential attrition by gaming group could bias results

We have added a section on missing data. The proportion of missing data ranged from 0% for video game consumption at baseline (which was used for grouping) to 13% (impulsivity) at baseline and increased with each wave (See Table S1).

Missing data was not equally distributed between groups and was higher in novel-gamers compared to other groups, except for PG and VG hours at baseline (Figure S1). To reduce possible bias due to differential attrition between groups, our main analyses focused on completers, i.e., participants who had data at wave 1 and 3, and no missing-data imputation technique was applied. This better controls for potential bias related to differential attrition between groups.

This has been clarified lines 256-262 of the revised MS and in the supplementary information, Table S1 and Figure S1.

4. Grouping Strategy

Consider: Justify quantile-based grouping or explore:

Theory-driven cutoffs (e.g., APA's 30 hours/week mentioned line 245)

Data-driven approaches (latent profile analysis)

Sensitivity analyses with different grouping schemes

We used a data-driven approach to categorize participants in quartiles according to their video game consumption at study inception. This choice ensures an equal number of participants in each group. In line with our hypothesis of non-linear associations between gaming time and problematic gaming, at least 3 groups were needed. We note that, among the four groups identified, we are well aligned with the theory-driven APA cutoff of 30 hours/week defining the heavy-gamers group. A smaller number of groups (i.e., tertiles) would result in lower cutoff, whereas a higher number (five or more) would result in smaller groups and thus lower power.

5. Novel-Gamers Definition

Lines 249-259 reveal measurement timing issues

Required clarification: Were questions about "usual" gaming and "past year" IGD symptoms administered in the same session or different sessions?

This affects interpretation of results for novel-gamers substantially.

All surveys were administered in the same session. At each wave, participants were asked about their usual gaming consumption and their problematic gaming symptoms during the past year.

Moderate Issues:

6. Effect Size Interpretation

While the stratified findings are interesting, the practical significance needs discussion

Even in novel-gamers, VG hours explain only 12% of IGD variance

Add: Discussion of what these effect sizes mean for intervention design

We thank the reviewer for raising this important point. We have now added the following text to the discussion lines 592-610 which provides a benchmark for interpretation of effect-sizes magnitudes as well as a comparison with effect-sizes from CBT intervention studies to situate their practical significance.

7. Exploratory Analyses (Figure 3)

Apply FDR correction for multiple comparisons

Be more cautious about interpreting "sneaky" and "deception" symptoms (lines 342-351)

These are interesting hypotheses but need replication

For large sample sizes, we followed the general recommendation to rely on effect sizes rather than significance levels. The results of analysis by symptom (i.e., item of the problematic gaming scale) are indeed presented as exploratory and in need of replication. We have further clarified the limitations in the discussion lines 556-571.

8. Temporal Ordering

Strengthen claims: The phrase "changes in VG consumption influenced changes in IGD" (lines 26-27) implies causation that difference scores cannot establish

Suggest rewording throughout to reflect associations rather than causal effects

We have rephrased in terms of association rather than influence.

9. Alternative Explanations

The discussion could address:

Why does impulsivity matter more for heavy gamers? (addressed briefly)

Could reverse causation explain some findings?

Role of game genres, social gaming, etc.

We have amended the discussion to more directly address these points (lines 514-543).

Minor Issues:

10. Methods Detail

SI section mentions IGD, VG consumption, impulsivity measures but main text should briefly describe:

How many items in each scale?

Response formats?

Reliability estimates?

The number of items and response formats are now available in the methods section. We have added the Cronbach’s alpha for each measure (0.70 for PG, 0.69 for impulsiveness and 0.78 for family environment) in the relevant section of the methods.

11. Table 1

Add column showing attrition rates by group

Because attrition varies across groups and measures, we have added a new supplementary Table S2 which shows the number of observations available for each variable and group at wave 1 and wave 3, with corresponding attrition rates.

Consider adding baseline comparisons of groups on outcome measures

Baseline differences are reported in supplementary results (section 3.1).

12. Figure 1

Very informative but consider adding:

Sample sizes at each timepoint

Statistical tests of mean changes

Sample sizes at each time point are now provided in table S2 for each group and measure. In addition, we have added the total sample size at each wave and measure to the legend of Figure 1.

Tests of mean changes between wave 1 and wave 3 are available in supplementary results, section 3.1.

13. Supplementary Materials

Consider moving Table S1 to main text - these are primary results.

Provide complete correlation matrices for all variables.

Table S1 (now S3) contains the coefficients plotted in Figure 2 and thus could be viewed as redundant. For now, given the length of the paper, we have kept that Table in the supplementary information but would be happy to move it to the main paper if deemed appropriate. Such information is indeed important for future systematic reviews and meta-analyses.

As asked, we have added a new Figure S2 in Sup Mat showing the full correlation matrix.

Strengths to Highlight:

Novel stratification approach addresses real heterogeneity in the literature

Large, longitudinal sample

Sophisticated control for confounds via SCA

Practical implications for tailored interventions

Transparent acknowledgment of exploratory analyses

We thank the reviewer for their suggestion of highlighting the strength of this work. It is now done lines 559-574 of the revised MS.

Suggested Additional Analyses:

Moderation analyses: Formally test whether gaming group moderates VG hours→IGD and impulsivity→IGD relationships

Dose-response: Within each group, is there a linear relationship between change in VG hours and change in IGD?

Bidirectional effects: Even with difference scores, test whether baseline IGD predicts change in VG hours

We have added a moderation analysis, which shows that the slope (dose-response) of the relationship between changes in problematic gaming and changes in gaming hours is steeper in novel-gamers. In contrast, the relationship between changes in problematic gaming and changes in impulsivity is steeper in heavy-gamers. These results, which are summarized in Figure S6 and in the main in the main text (lines 476-481), are consistent with the main analysis, confirming the moderating influence of gaming group on the relationship between VG hours and PG and between Impulsivity and PG.

To more formally test the directionality of the effects, we also added CLPM and RICLPM analyses (also requested by reviewer 1).

Discussion Enhancements:

Compare effect sizes to other longitudinal IGD studies

Discuss clinical vs. statistical significance more explicitly

Address limitations more comprehensively (single cultural context, self-report measures, attrition)

We have added a dedicated section to address these points in the discussion lines 592-610.

We also added a section to acknowledge the limitations of the study, lines 575-589.

Clarify intervention implications: How would one "introduce gaming progressively" (line 31)?

We have added examples in the abstract (last sentence).

6. PLOS authors have the option to publish the peer review history of their article (what does this mean?). If published, this will include your full peer review and any attached files.

If you choose “no”, your identity will remain anonymous but your review may still be made public.

Do you want your identity to be public for this peer review? For information about this choice, including consent withdrawal, please see our Privacy Policy.

Reviewer #1: No

Reviewer #2: No

REFERENCES (cited in the response)

Ji, Y., Yin, M. X. C., Zhang, A. Y., & Wong, D. F. K. (2022). Risk and protective factors of Internet gaming disorder among Chinese people: A meta-analysis. The Australian and New Zealand Journal of Psychiatry, 56(4), 332–346. https://doi.org/10.1177/00048674211025703

Sallis, J. F., Prochaska, J. J., & Taylor, W. C. (2000). A review of correlates of physical activity of children and adolescents. Medicine and Science in Sports and Exercise, 32(5), 963–975. https://doi.org/10.1097/00005768-200005000-00014

Twenge, J. M., Martin, G. N., & Campbell, W. K. (2018). Decreases in psychological well-being among American adolescents after 2012 and links to screen time during the rise of smartphone technology. Emotion, 18(6), 765–780. https://doi.org/10.1037/emo0000403

Warburton, W. A., Parkes, S., & Sweller, N. (2022). Internet Gaming Disorder: Evidence for a Risk and Resilience Approach. International Journal of Environmental Research and Public Health, 19(9), Article 9. https://doi.org/10.3390/ijerph19095587

Zhuang, X., Zhang, Y., Tang, X., Ng, T. K., Lin, J., & Yang, X. (2023). Longitudinal modifiable risk and protective factors of internet gaming disorder: A systematic review and meta-analysis. Journal of Behavioral Addictions, 12(2), 375–392. https://doi.org/10.1556/2006.2023.00017

Attachment Submitted filename: MS_PLOSone_responses_v4.docx

# Peer review: Decision Letter 1

**DOI:** 10.1371/journal.pone.0356080.r003

**Authors:** Marc Potenza

13 Apr 2026

Dear Dr. Bediou,

Please submit your revised manuscript by May 28 2026 11:59PM. If you will need more time than this to complete your revisions, please reply to this message or contact the journal office at plosone@plos.org. When you're ready to submit your revision, log on to https://www.editorialmanager.com/pone/ and select the 'Submissions Needing Revision' folder to locate your manuscript file.

- A letter that responds to each point raised by the academic editor and reviewer(s). You should upload this letter as a separate file labeled 'Response to Reviewers'.
- A marked-up copy of your manuscript that highlights changes made to the original version. You should upload this as a separate file labeled 'Revised Manuscript with Track Changes'.
- An unmarked version of your revised paper without tracked changes. You should upload this as a separate file labeled 'Manuscript'.

If you would like to make changes to your financial disclosure, please include your updated statement in your cover letter. Guidelines for resubmitting your figure files are available below the reviewer comments at the end of this letter.

If applicable, we recommend that you deposit your laboratory protocols in protocols.io to enhance the reproducibility of your results. Protocols.io assigns your protocol its own identifier (DOI) so that it can be cited independently in the future. For instructions see: https://journals.plos.org/plosone/s/submission-guidelines#loc-laboratory-protocols. Additionally, PLOS ONE offers an option for publishing peer-reviewed Lab Protocol articles, which describe protocols hosted on protocols.io. Read more information on sharing protocols at https://plos.org/protocols?utm_medium=editorial-email&utm_source=authorletters&utm_campaign=protocols.

As the corresponding author, your ORCID iD is verified in the submission system and will appear in the published article. PLOS supports the use of ORCID, and we encourage all coauthors to register for an ORCID iD and use it as well. Please encourage your coauthors to verify their ORCID iD within the submission system before final acceptance, as unverified ORCID iDs will not appear in the published article. *Only*  the individual author can complete the verification step; PLOS staff *cannot*  verify ORCID iDs on behalf of authors.

We look forward to receiving your revised manuscript.

Kind regards,

Marc N Potenza

Academic Editor

PLOS One

**Journal Requirements:**

If the reviewer comments include a recommendation to cite specific previously published works, please review and evaluate these publications to determine whether they are relevant and should be cited. There is no requirement to cite these works unless the editor has indicated otherwise.

[Note: HTML markup is below. Please do not edit.]

Reviewers' comments:

Reviewer's Responses to Questions

**Comments to the Author**

Reviewer #1: (No Response)

Reviewer #3: (No Response)

**********

2. Is the manuscript technically sound, and do the data support the conclusions?

Reviewer #1: Partly

Reviewer #3: No

**********

3. Has the statistical analysis been performed appropriately and rigorously?

Reviewer #1: Yes

Reviewer #3: No

**********

4. Have the authors made all data underlying the findings in their manuscript fully available?

The PLOS Data policy

Reviewer #1: Yes

Reviewer #3: Yes

**********

5. Is the manuscript presented in an intelligible fashion and written in standard English?

Reviewer #1: Yes

Reviewer #3: Yes

**********

Reviewer #1: Issue: Lines 52-57. It isn’t clear what was studied longitudinally. Was it persistence of IGD symptoms? Was it treatment?

Response: The meta-analysis by Zhuang et al. focused on studies with a longitudinal design, whether including a treatment (intervention) or not (i.e. IGD persistence). This has been clarified in the text.

Adequacy of author response: Not adequately resolved. Longitudinal effects of what on what? This isn’t clear. Is it effects of problematic levels of gaming on mental health-related outcomes? Are the modifiable factors aspects of problematic gaming that can be modified through intervention? I don’t think this reworded section is sufficiently clear.

Issue: Lines 57-58. Also, it is not true that impulsivity and game time are the two strongest predictors.

At r=.11, impulsivity was a weaker predictor than self control (r=-.27) and parent child relationship (r=-.15). If self control and impulsivity have differing effect sizes then they are different constructs and should not be treated interchangeably.

In terms of game time, the authors note in lines 84-93 that the correlation between gaming time and IGD is generally small, and this is my experience. This undermines the contention that game time is the strongest predictor of IGD.

Overall, the second paragraph could be rewritten for clarity and focus.

Response: We appreciate the reviewer drawing our attention to the difference between impulsivity and self-control. We have clarified that self control (the positive side) refers to the capacity to generate adapted behavioral responses, often tested in the context of questionnaires presenting everyday life situations. Impulsivity (the down side) refers to the personality trait which can be measured either using “objective” task based measures or using subjective questionnaires such as the UPPS or the Barratt Impulsiveness Scale, as in the present work. To avoid confusion, we have revised the text to clarify that these constructs are distinct, and we also discuss how these two constructs interact.

As noted by the reviewer, the meta analysis referred above and in later in the discussion (lines 84–93), the correlation between game time and problematic gaming is generally small. We have rewritten to properly characterize its predictive strength. Our point was rather to draw attention to the fact that game time remains one of the most consistently examined variables in the literature. We have rephrased accordingly.

The second paragraph of the introduction (and in fact the whole introduction) has been substantially rewritten to increase clarity and focus, as well as to further acknowledge the existence of other modifiable risk and protective factors (see next comments from Reviewer 1).

Adequacy of author response: Resolved. The authors have restructured this section for clarity and it makes more conceptual sense.

Issue: Paragraph beginning line 94

This paper seems to be about predictors of IGD rather than outcomes, and this paragraph on the impact of IGD on mental health seems to come from nowhere and not be linked to the central tenets of the paper. If this paper is centrally interested in outcomes as well as predictors then the introduction needs to be more clearly framed with the aims of the paper stated clearly up front.

Response: The reviewer is correct. We have removed the section on the relationship between gaming and other health consequences of problematic gaming as this is not directly relevant to the present study.

Adequacy of author response: Resolved.

Issue: Lines 136-138. I do not agree that “the most common modifiable risk and protective factors for IGD identified in past research” are “gaming time, impulsiveness, and family environment”. There are many risk and protective factors for IGD (for example, see the Warburton et al 2022 risk and resilience paper on IGD with a large range of factors, Li et al 2023 with risk and protective factors related to GD, Jeong et al 2020 on several factors associated with IGD persistence plus others; there are several papers on risk and protective factors for problematic social media use that may also be relevant). Many such factors are modifiable. I think it is important that all key risk and protective factors be acknowledged in the introduction, with a compelling reason then given as to why the factors of interest in this study were singled out.

Response: We thank the reviewer for pointing out these additional studies. We now provide an extended list of modifiable risk and protective factors in the introduction, including the references suggested by Reviewer 1 as well as new references to other relevant work. We also made explicit that the choice of factors of interest was guided by the epidemiological triad framework to cover factors associated with each level: the agent (gaming consumption), the host (impulsivity) and the environment (family environment).

Adequacy of author response: Resolved. The authors have made substantial rewrites around the risk and protective factors, and made a clearer rationale for the study.

Issue: Lines 145-157. Discusses results rather than hypotheses. I will deal with the age of the data elsewhere.

Response: We have rephrased this section of the introduction to present our hypotheses rather than results.

Adequacy of author response: Resolved, again through substantive rewriting/

Issue: Overall, the introduction lacked clarity and focus and for me did not provide a compelling argument for the methodology of the study conducted.

Response: We thank Reviewer 1 for their constructive feedback. We have made substantial changes to improve the clarity and focus of the introduction as well as to better introduce our hypotheses and how they justify the methodology.

Adequacy of author response: Resolved (although note there are still some specific issues to address).

Method

Issue: The single biggest issue with this paper is the age of the data (2007-2009), all of which predates by several years the proposed diagnostic criteria for IGD (2013). Apart from the issue of publishing data now nearly 20 years old, how can a person meaningfully study data purported to measure a phenomenon that was not created, reported or measured until several years afterward? At the very least this study must be reframed as being about problematic gaming rather than as being related to a disorder not conceptualised until after the data were collected.

Response: We acknowledge this anachronism and have now rewritten the manuscript to refer to problematic gaming (PG) rather than IGD. The questions use in the present study cover 9 of the 10 symptoms of the DSM-V criteria for IGD with the one divergence concerning the continuation question in the DSM-V which is absent and replaced by a question about negative consequences (sneaky behaviors) in the present study. This has been clarified by adding a column with the IGD items to Table S4 and updating the legend to mention the diverging item.

Adequacy of author response: Not resolved and, for me, must be resolved for the paper to be publishable. The paper starts with the following: “Since it was first introduced in the research appendix of the DSM-V [1] as a condition for further study, Problematic Gaming (PG) has garnered increasing attention in adolescent mental health due to its potential impact on psychological well-being and social functioning [2]. We refer here to PG as signaling the kind of problematic behavior, that is now also known as ‘Internet Gaming Disorder’ (following the DSM /APA terminology) or ‘Gaming Disorder’ according to the 11th Revision of the International Classification of Diseases (ICD-11) [3,4]”.

I have numerous issues with this.

1. Problematic gaming (PG) was not introduced in the research appendix of the DSM-V as stated here. Rather, Internet Gaming Disorder (IGD), not PG, was introduced into the ‘Conditions for further study’ section in 2013. IGD was the first suggested clinical diagnosis. However interest in PG significantly predates this inclusion, and much of the research referred to in this paper also predates 2013.

2. Problematic Gaming is not a clinical diagnosis – it refers to gaming that is problematic, at both clinical and sub-clinical levels. Thus it would include people with:

a. IGD and GD

b. Hazardous Gaming from ICD-11

c. Gaming at problematic but sub-clinical levels, as indicated by elevated scores on measures of problematic gaming, and scores that are elevated but sub-clinical on measures for IGD and GD.

3. Because problematic gaming encompasses the above, PG is not synonymous with IGD and GD as suggested above, but is more synonymous with Hazardous Gaming.

In my view this introduction should say something more like, “Although clinical diagnoses for disordered gaming are only fairly recent, with Internet Gaming Disorder (IGD) appearing as a condition for further study in the DSM-V (APA, 2013), and Gaming Disorder as a diagnosis in the ICD-11 (WHO, 2022), problematic gaming (PG) has been identified and studied for considerably longer. PG is characterised by ….. “ going on to describe what it encapsulates, noting it can be measured as hazardous gaming symptoms (ICD-11), elevated but sub-clinical scores on measures for IGD, and elevated scores on other measures of problematic gaming, many of which include criteria that have been published as IGD criteria. There needs to be complete clarity about what PG is, to set up this paper. Crucially, the rest of the paper then needs to align with this definition of PG.

Age of data. I cannot find where the authors have addressed the age of the data itself and provided a justification for using data so old. The authors need to address this issue.

Issue: It is not sufficient to say that the measures and methodology are reported elsewhere. This paper must stand alone in terms of describing the sample and measures used here. Given the contentiousness of claiming IGD was measured (see below) it is crucial that all measures be made clear and described in sufficient detail. It is also important that key sample characteristics are reported.

Response: We have moved the details about the sample and the measures from the supplementary information to the methods section of the main manuscript.

Adequacy of author response: Resolved

Issue: It is also purported that the study measured IGD. This cannot be factually true given that no such diagnosis existed at the time of the study.

Response: This important point is now clarified in the introduction, where we note that we use the term problematic gaming in this work to cover PG, GD and IGD, as the DSM scales have changed.

Adequacy of author response: Resolved

Results

Issue: Should decimals be to two decimal points (e.g., Table 2)

Response: We have added the second decimals that were missing.

Adequacy of author response: Resolved

Issue: I think it is important to note that the IGD score is [presumably – we never see the measure] a score of IGD symptomology providing a continuous numeric measure on a continuum, rather than an indication of actually having a gaming disorder. So this is not really a study of IGD or any sort of disorder in a way, it is a study of the level of symptoms, with low levels at one end and higher levels at the other, with no way of knowing which, if any, participants had a gaming disorder. In line 335 diagnosis of IGD is discussed in relation to the data. Again, these data predate the diagnosis or any measure of IGD and the data are continuous without categorising IGD.

Response: We have clarified that the dependent variable is the sum of all 10 items, which are available in Table S2, and represents a measure of the severity of PG symptomatology.

Adequacy of author response: Resolved

Issue: Paragraph starting line 248. It is not simply the case that novel gamers had some IGD symptoms at wave 1 – their levels were nearly as high as at wave 3 and nearly as high as all other groups. Indeed higher than the ‘low’ group. This does not seem to be entirely an artefact of the wording or timing of presentation of measures – for me it is a significant anomaly that casts doubt on the measure of IGD and all the IGD data.

Response: We thank the reviewer for raising this important point. It should not come as a surprise that, amongst novel gamers, there are children who are not allowed to play video game at home and yet may engage in video game play outside of the family setting. This disconnect is not surprising and in fact aligned with the most frequently reported “symptoms” at baseline in novel gamers being, from most frequent to least frequent: items 9 (loss of control / unsuccessful attempts), followed by item 6 (escape), item 2 (withdrawal), item 7 (loss of interests), and item 1 (preoccupation).

This has now been addressed lines 360-370 of the revised MS.

Adequacy of author response: Not resolved. I do not think this explanation is sufficient or clear. Why would kids who only play outside the house, and have less opportunity to play, have PG symptoms close to those of intermediate and heavy gamers? Why would gaming only outside the home result in substantial problems with loss of control over gaming and preoccupation? Is there any reason this group would have disproportionately high loss of other interests or gaming for withdrawal? Why would their gaming hours be the same as intermediate gamers and close to that of heavy gamers when they have less opportunities to play? Something still seems wrong. Maybe the problem is the categorisation – perhaps this group aren’t really novel gamers? Is there a cultural explanation – parenting is so strict in Singapore many kids spend excessive time out of the home gaming? (If so, is there evidence of this). Either way, I think this section (lines 372 to 389) needs to be completely re-thought and revised.

Issue: Overall I found the results hard to follow and interpret, but having said that do think the findings are interesting and publishable.

Response: We thank the reviewer and have rephrased the results and discussion to make them easier to follow.

Adequacy of author response: Resolved (except issues otherwise noted)

Discussion

Issue: Line 365. Is ‘remarkable’ the best word for a finding that is strongly in line with past research? Maybe noteworthy ….

We have replaced remarkable with noteworthy

Adequacy of author response: Resolved

Issue: I am not sure one can categorise IGD as an impulse control problem (line 383). Rather, impulsiveness is one risk factor. It is certainly not an adequate description of the disorder.

Response: We thank the reviewer for this clarification. We have removed this sentence and rephrased part of our text. We agree with the reviewer that it does not provide direct evidence for considering PG as an impulse control problem.

Adequacy of author response: Resolved

Issue: Overall I found the discussion to be lacking in depth, entirely omitting the clear limitations of the paper or adequately situating findings in existing literature.

Response: The discussion now better situates our findings within the existing literature. We have also added a limitation section. In particular, we now emphasize that the direction and magnitude of the observed effects is consistent with the prior work discussed in the introduction. We also acknowledge the inherent limitations of our measures of family environment and of impulsivity, especially when it comes to capturing more complex dynamics associated with the constructs of social environment and of impulse regulation.

Adequacy of author response: The Discussion is much improved. I would note (lines 554-566) that a recent meta-analysis (Moshel et al 2024) found links between gaming disorders and reduced executive function. Whilst the biggest effect was on attention, global EF including inhibitory control was also impacted. Thus, whilst problematic gaming over time seems unlikely to change trait impulsivity per se, it seems possible it may increase inhibitory control problems, which would look like greater impulsivity over time, similar to the finding here.

Minor issues

Issue: There is often not a gap between a word and a following reference number in brackets.

Response: Missing spaces have been added.

Adequacy of author response: Resolved

In Summary

Issue: This paper has a number of serious issues – the age of the data, the measure of IGD predating the existence of the diagnosis, the lack of detail about key parts of the study, and a lack of clarity and focus across the paper. I do think the results are interesting but would need to be presented in a heavily redrafted paper with a completely new focus (not IGD).

Response: We thank the reviewer for their thoughtful and constructive feedback on our manuscript. We hope our interesting results are now better contextualized thanks to the novel introduction, the clarification about the collected measures, the amount of missing data and attrition rates across waves and measures, as well as the updated discussion. We thank the reviewer for their suggestions as to how to strengthen our paper.

Adequacy of author response: The authors have gone to considerable trouble to revise this paper and have generally done a quite good job. There are still some issues that I think need to be resolved before this can be published, with a better framing of PG crucial, but once these issues are resolved I think this paper will make an interesting contribution to the field.

Reviewer #3: Thank you for the opportunity to review this manuscript. The study addresses an interesting question about whether the associations between established risk factors and problematic gaming (PG) symptoms are moderated by baseline gaming habits in a longitudinal sample. The research question is timely, the sample size is substantial, and the application of Specification Curve Analysis is a notable methodological strength.

However, I have a number of concerns, some of which are substantial, that I believe need to be addressed.

1. The manuscript does not disclose in either the title or the abstract that this is a secondary analysis of an existing longitudinal dataset originally collected between 2007 and 2009 (Gentile et al., 2011; Choo et al., 2010). This is an important transparency issue. Readers should be made aware from the outset that the study design, measures, and sampling decisions were made for a different primary purpose, and that the current analyses were not pre-specified in that original study. PLOS ONE and broader reporting standards for secondary analyses require this to be clearly stated. The abstract also does not report basic methodological details, including sample size, country of origin, study period, or the key inclusion criteria, which would be expected of a methods-transparent abstract. Both issues should be corrected.

2. The VG hours composite was derived by multiplying self-reported hours on a typical weekday by five and on a typical weekend day by two. This approach risks overestimating weekly gaming time for participants who do not game every day, since multiplying a 'typical play session' figure across all days of a given type attributes gaming to days when no gaming may have occurred. A participant who games on three of five weekdays for two hours each session would report a 'typical weekday' figure of two hours, yielding a composite weekday contribution of ten hours rather than the actual six. Second, and more importantly, the relationship between this self-reported composite and actual gaming behavior is likely noisy. Research has shown that self-reported gaming time correlates only moderately with PG symptoms (i.e., r = 0.25–0.50 in comprehensive reviews; King et al., 2020 https://doi.org/10.1016/j.cpr.2020.101831) and, critically, that self-reported gaming time correlates more strongly with available leisure time than with problematic patterns of play (Stevens et al., 2025, Addiction, https://doi.org/10.1111/add.70192). The authors should acknowledge this limitation explicitly and consider its implications for interpreting the finding that VG hours drives PG change in novel gamers.

3. In the introduction (line 97, approximately), the authors refer to an effect size of r = 0.29 as "small-to-moderate." Yet, on another occasion they describe a pooled longitudinal r = 0.31 as a "strong" correlation for gaming time predicting PG. These two characterisations are inconsistent with each other and, more broadly, r = 0.31 is not conventionally regarded as a strong effect. A citation is needed to justify calling this effect strong, and the authors should apply consistent terminology throughout.

4. The Cronbach's alpha for the PG scale is reported as 0.70 (95% CI: 0.67–0.72), which is generally considered borderline acceptable and, at the lower confidence interval bound, would fall into the range many methodologists would flag as problematic. The authors note this in passing but do not pursue the implications sufficiently. Given the unusual item composition of this scale, particularly the inclusion of a "sneaky behaviors" item drawn from DSM-IV pathological gambling (rather than any IGD framework), it would be worth examining whether this item is responsible for attenuating the alpha and whether removing it and re-running the core analyses would alter the findings. Additionally, the authors should report omega coefficients (e.g., McDonald's omega) rather than or alongside Cronbach's alpha, as omega is a more robust reliability estimate that does not assume tau-equivalence and is now preferred in the psychometric literature. This goes for all scales used in this study.

5. The PG measure is derived from DSM-IV pathological gambling criteria and was administered between 2007 and 2009. It predates the DSM-5 IGD criteria (2013) and the ICD-11 Gaming Disorder definition (2019). The alignment between this instrument and current diagnostic frameworks is therefore partial at best. Nine of the ten items reportedly correspond to IGD criteria, but the tenth item (sneaky behaviors) does not, and this item appears to drive a notable proportion of the VG hours effect in novel gamers (Figure 3). The authors acknowledge this in the item-level exploratory analysis but do not adequately address the construct validity concern in the main methods or limitations sections. Reviewers and readers of a 2025/2026 manuscript should be made aware that the core outcome measure was not designed or validated against either current diagnostic system.

6. The manuscript examines family environment as the primary interpersonal predictor but does not consider peer-level factors. This is a meaningful gap given the extensive literature on the role of school belonging, peer victimization, online social dynamics, and social isolation as risk factors for PG, particularly in Chinese and East Asian adolescent samples. A number of studies using similar Singaporean and Chinese cohorts have highlighted that peer and school factors often explain variance in PG beyond family environment. The authors should at minimum acknowledge this limitation and note that the absence of peer-level measures constrains the generalizability of the conclusion that interpersonal factors show relatively weak effects.

7. The manuscript does not distinguish between pathological engagement and elite or professional gaming, and the heavy-gamer category (averaging 57 hours per week, up to 106 hours) almost certainly includes individuals whose gaming reflects high skill and commitment rather than disorder. Research on professional gamers and eSports athletes shows that this group does not show the same neurobiological or psychosocial patterns as individuals with GD despite comparable gaming hours (see e.g., Choi et al., 2021; https://doi.org/10.2196/25793). The authors' framework could be strengthened by acknowledging this heterogeneity within the heavy-gamer group, which may attenuate the observed effect of VG hours on PG in that group.

8. The observation that novel gamers (who report not usually playing at baseline) nonetheless endorse PG symptoms is acknowledged by the authors but the explanation offered is speculative. It is plausible that this reflects measurement artefact (i.e., children who cannot access games at home but occasionally play elsewhere being classified as non-gamers despite some exposure) but it is equally possible that some participants misunderstood the item ordering or that the "over the past year" framing captures occasional play episodes. This issue creates a conceptual ambiguity at the heart of the novel-gamer subgroup analysis: if novel gamers are reporting PG symptoms without habitual gaming, the construct being measured may be something other than gaming-related harm (e.g., general sensation-seeking, impulsivity, or a response set). The authors should address this more deeply.

9. The authors state they focused on completers (participants with data at waves 1 and 3) and did not apply missing data imputation. Attrition was not uniform across groups, with higher rates among novel gamers. Completers-only analysis in the presence of differential attrition risks producing biased estimates of longitudinal associations, particularly if dropout is related to the outcome (i.e., if adolescents whose PG worsened were more likely to drop out). The authors acknowledge some missing data but do not adequately address whether their completers-only approach may have introduced systematic bias. Multiple imputation or at minimum a sensitivity analysis comparing completers and non-completers on key baseline variables should be considered.

Minor Concerns

P-values throughout Table 3 are reported as "0.00," which is not a valid p-value. Values should be reported to three decimal places, or as "< .001" where appropriate. This is a basic statistical reporting standard and should be corrected throughout.

The supplementary materials note that this dataset has been used in numerous prior publications. The authors should provide a clearer account of what the present analysis adds that has not been reported elsewhere, beyond the novel stratification by baseline gaming group.

The data were collected in Singapore between 2007 and 2009. The gaming landscape has changed substantially since then, particularly in the prevalence of mobile gaming, online multiplayer formats, and free-to-play monetization models. The authors should be explicit about the limitations of extrapolating findings from this historical cohort to contemporary populations.

Reference 46 (cited to justify effect size thresholds) is listed as a Google search URL, which is not a citable source. The authors should cite the primary source (Field, 2013).

Overall, the study raises an interesting hypothesis about moderating effects of baseline gaming habits and uses appropriate analytic techniques. However, the concerns above, particularly around the secondary analysis transparency, gaming time measurement validity, instrument age and construct alignment, and handling of attrition, are substantial enough that major revisions are warranted.

**********

what does this mean?). If published, this will include your full peer review and any attached files.

If you choose “no”, your identity will remain anonymous but your review may still be made public.

**Do you want your identity to be public for this peer review?**  For information about this choice, including consent withdrawal, please see our Privacy Policy

Reviewer #1: No

Reviewer #3: **Yes:**  Matthew W. R. Stevens

**********

[NOTE: If reviewer comments were submitted as an attachment file, they will be attached to this email and accessible via the submission site. Please log into your account, locate the manuscript record, and check for the action link "View Attachments". If this link does not appear, there are no attachment files.]

To ensure your figures meet our technical requirements, please review our figure guidelines: https://journals.plos.org/plosone/s/figures

You may also use PLOS’s free figure tool, NAAS, to help you prepare publication quality figures: https://journals.plos.org/plosone/s/figures#loc-tools-for-figure-preparation.

NAAS will assess whether your figures meet our technical requirements by comparing each figure against our figure specifications.

# Author comment: Author response to Decision Letter 2

**DOI:** 10.1371/journal.pone.0356080.r004

29 May 2026

The point-by-point response to reviewers has been uploaded as a separate document

Attachment Submitted filename: ResponseLetter_R2_PONE-D-25-39575R1.docx

# Peer review: Decision Letter 2

**DOI:** 10.1371/journal.pone.0356080.r005

**Authors:** Marc Potenza

21 Jul 2026

Dear Dr. Bediou,

Thank you for submitting your manuscript to PLOS ONE. After careful consideration, we feel that it has merit but does not fully meet PLOS ONE’s publication criteria as it currently stands. Therefore, we invite you to submit a revised version of the manuscript that addresses the points raised during the review process.

plosone@plos.org. When you're ready to submit your revision, log on to https://www.editorialmanager.com/pone/ and select the 'Submissions Needing Revision' folder to locate your manuscript file.

- A letter that responds to each point raised by the academic editor and reviewer(s). You should upload this letter as a separate file labeled 'Response to Reviewers'.
- A marked-up copy of your manuscript that highlights changes made to the original version. You should upload this as a separate file labeled 'Revised Manuscript with Track Changes'.
- An unmarked version of your revised paper without tracked changes. You should upload this as a separate file labeled 'Manuscript'.

If you would like to make changes to your financial disclosure, please include your updated statement in your cover letter. Guidelines for resubmitting your figure files are available below the reviewer comments at the end of this letter.

If applicable, we recommend that you deposit your laboratory protocols in protocols.io to enhance the reproducibility of your results. Protocols.io assigns your protocol its own identifier (DOI) so that it can be cited independently in the future. For instructions see: https://journals.plos.org/plosone/s/submission-guidelines#loc-laboratory-protocols. Additionally, PLOS ONE offers an option for publishing peer-reviewed Lab Protocol articles, which describe protocols hosted on protocols.io. Read more information on sharing protocols at https://plos.org/protocols?utm_medium=editorial-email&utm_source=authorletters&utm_campaign=protocols.

As the corresponding author, your ORCID iD is verified in the submission system and will appear in the published article. PLOS supports the use of ORCID, and we encourage all coauthors to register for an ORCID iD and use it as well. Please encourage your coauthors to verify their ORCID iD within the submission system before final acceptance, as unverified ORCID iDs will not appear in the published article. *Only*  the individual author can complete the verification step; PLOS staff *cannot*  verify ORCID iDs on behalf of authors.

We look forward to receiving your revised manuscript.

Kind regards,

Marc N Potenza

Academic Editor

PLOS One

Journal Requirements:

If the reviewer comments include a recommendation to cite specific previously published works, please review and evaluate these publications to determine whether they are relevant and should be cited. There is no requirement to cite these works unless the editor has indicated otherwise.

Additional Editor Comments:

Please see the review for details.

[Note: HTML markup is below. Please do not edit.]

Reviewers' comments:

Reviewer's Responses to Questions

**Comments to the Author**

Reviewer #3: All comments have been addressed

Reviewer #4: (No Response)

**********

2. Is the manuscript technically sound, and do the data support the conclusions?

Reviewer #3: Yes

Reviewer #4: Yes

**********

3. Has the statistical analysis been performed appropriately and rigorously?

Reviewer #3: Yes

Reviewer #4: I Don't Know

**********

4. Have the authors made all data underlying the findings in their manuscript fully available?

The PLOS Data policy

Reviewer #3: Yes

Reviewer #4: No

**********

5. Is the manuscript presented in an intelligible fashion and written in standard English?

Reviewer #3: Yes

Reviewer #4: Yes

**********

Reviewer #3: Dear authors,

Thank you for your timely and thoughtful revisions. All comments and concerns have now been adequately addressed. Wishing you all the best with your submission.

Reviewer #4: Thank you for the opportunity to review this interesting manuscript. This study examined how differences in gaming engagement, impulsivity, and family environment relate to changes in problematic-gaming symptoms in adolescents with varying baseline levels of gaming engagement (i.e., duration of play). Please see my suggestions for the manuscript below:

Abstract:

- Please introduce the “VG” abbreviation when video games are first mentioned.

- Please include a space between “heavygamers.” In relevance to this, I would also consider using more person-centered language throughout the manuscript (including figures and tables). For example, “heavy gamers” may be replaced with “heavy gaming” or “heavy-gaming group.”

Introduction:

- Please consider revising the first sentence of the introduction to reflect that it was Internet Gaming Disorder (not the broader construct of problematic gaming) that was included in Section III as a condition for further study.

- It may be helpful to include in-text citations in the “host” and “environment” sections (e.g., studies showing relationships between depression and problematic gaming).

- I would recommend removing the word “established” in the subheading.

- Replacing “predicting” with “associated with” may be preferable in the following sentence: “…predicting subsequent symptoms severity is strong.”

- I believe the word “and” is missing here: “…perseverance, sensation seeking.”

- Rewriting for clarity and adding in-text citations here may strengthen the sentence: “impulsivity is associated with difficulties considering alternative solutions, with acting precipitously and ignoring consequences and is recognized as a predictive factor in the development of maladaptive behaviors resulting from poor regulation of positive and negative emotions.”

- For rows 110-113, please consider clarifying that this is in the context of problematic gaming.

- I would recommend proofreading the manuscript. For example, there appear to be repeated words in row 172. Also, some words appear to be capitalized, such as impulsiveness and family environment in row 198.

- Considering that the presentations of results (in the results section of the manuscript) are stratified according to group, it would be important to specify how many hours of gaming were considered to be occasional for the “occasional” gaming group both in the introduction and methods. Currently, in the introduction, it states “not usually playing,” but it would be important to report a specific number here or clarify how this was determined (it appears this information can be found in the results section, rows 406-208, but it may be helpful to provide this clarification earlier).

Methods:

- Please consider changing 3’434 to 3,434

- It may be helpful to include some additional information regarding the procedure (e.g., was the survey delivered on laptops/phones or on paper? Were participants reimbursed? How exactly were the schools/students recruited?).

- The wording around “most” may be slightly confusing for readers in rows 232-233. Please consider clarifying the “most of the cohort was followed” section. If it is referring to participants who were retained over the three-year follow-up period, please explicitly state this.

- I suggest being more cautious with the wording in rows 243-245, as some DSM-IV pathological-gambling criteria (e.g., chasing losses, committing illegal acts, and relying on others for financial bailouts) were not transferred to IGD.

- I would also recommend slightly adjusting the wording for rows 242-243 (perhaps removing the “10-item” part) to better align with the following sentence in the referenced King et al. article: “Prior to the provisional DSM-5 criteria for GD, researchers would often adapt the criteria of other disorders (e.g., pathological gambling in the DSM-IV-TR).”

- Please include a space between “and13%”

- After the “measures included in this reanalysis” subheading, it would be helpful to include additional subheadings for the other sections (e.g., an “ethics” subheading for rows 306 -317 and also a “statistical analysis” subheading).

- Please correct the typo for “environement”

- Please change “ensures” to “ensure” in row 324.

- Some additional specific information would be helpful for the statistical analysis section. For example, what statistical package was used, how model fit and significance were evaluated, etc.

- Regarding the section describing the problematic gaming scale, it would be helpful to briefly summarize the 10 items, given that the results show “sneaky” and “deception” as separate symptoms.

Results:

- It appears that the number of hours of gaming per week that determines what group a participant is in is different from what is stated in the introduction. Also, regarding the heavy-gaming group, it would be helpful to add the range of hours. For the remaining groups, it would be helpful to add the mean.

Discussion:

- In the discussion or the introduction, it may be beneficial to provide a nuanced discussion of the limitations associated with categorizing participants into named gaming-engagement groups (“occasional, low, intermediate, or heavy”) solely on the basis of hours played.

- Regarding the fifth limitation, it may be helpful to consider discussing the use of the TimeLine FollowBack in previous gaming disorder studies (e.g., “A brief internet-delivered intervention for the reduction of gaming-related harm: A feasibility study”) to reduce the concerns stated.

- Please consider including a brief conclusion section (and subheading) at the end of the discussion.

**********

what does this mean?). If published, this will include your full peer review and any attached files.

If you choose “no”, your identity will remain anonymous but your review may still be made public.

**Do you want your identity to be public for this peer review?**  For information about this choice, including consent withdrawal, please see our Privacy Policy

Reviewer #3: **Yes:**  Matthew W. R. Stevens

Reviewer #4: **Yes:**  Jennifer Park

**********

[NOTE: If reviewer comments were submitted as an attachment file, they will be attached to this email and accessible via the submission site. Please log into your account, locate the manuscript record, and check for the action link "View Attachments". If this link does not appear, there are no attachment files.]

To ensure your figures meet our technical requirements, please review our figure guidelines: https://journals.plos.org/plosone/s/figures

You may also use PLOS’s free figure tool, NAAS, to help you prepare publication quality figures: https://journals.plos.org/plosone/s/figures#loc-tools-for-figure-preparation.

NAAS will assess whether your figures meet our technical requirements by comparing each figure against our figure specifications.

# Author comment: Author response to Decision Letter 3

**DOI:** 10.1371/journal.pone.0356080.r006

29 Jul 2026

Dear Dr. Potenza,

On behalf of my co-authors, I am pleased to submit the revised version of our manuscript entitled “Problematic gaming in occasional, low, intermediate or heavy gamers – not all screen time is created equal: a reanalysis” (Manuscript ID: PONE-D-25-39575R2) for further consideration for publication in PLOS ONE.

We sincerely thank you and the reviewers for your careful evaluation of our manuscript and for the constructive comments provided. We are particularly encouraged that Reviewer #3 considered all previous concerns to have been adequately addressed.

In response to Reviewer #4’s additional suggestions, we have carefully revised the manuscript throughout. The revisions include clarification of terminology, improved reporting of the methodology and statistical analyses, additional procedural details, clarification of the gaming-engagement group definitions, refinement of several sections of the Introduction and Discussion, correction of typographical and language issues, and the addition of a brief Conclusion section. We have also expanded the discussion of the study’s limitations and improved the description of the problematic gaming measure.

A detailed, point-by-point response to every comment is provided in the accompanying Response to Reviewers document. For ease of review, we have also submitted both a tracked-changes version of the manuscript and a clean version.

We believe that these revisions have strengthened the manuscript considerably and hope that it is now suitable for publication in PLOS ONE.

Thank you for your time and consideration. We appreciate the opportunity to revise our work and look forward to your decision.

Sincerely,

Benoît Bediou, PhD

on behalf of all authors

Reviewer #4: Thank you for the opportunity to review this interesting manuscript. This study examined how differences in gaming engagement, impulsivity, and family environment relate to changes in problematic-gaming symptoms in adolescents with varying baseline levels of gaming engagement (i.e., duration of play). Please see my suggestions for the manuscript below:

Abstract:

- Please introduce the “VG” abbreviation when video games are first mentioned.

Response: We now introduce VG in the abstract

- Please include a space between “heavygamers.” In relevance to this, I would also consider using more person-centered language throughout the manuscript (including figures and tables). For example, “heavy gamers” may be replaced with “heavy gaming” or “heavy-gaming group.”

Response: Thank you — the missing space has been added. On person-centered language, we agree with the spirit of the suggestion and now explicitly introduce the four groups as gaming-engagement categories defined by baseline weekly play (not clinical categories or stable identities), using person-centered phrasing (e.g., “adolescents in the heavy-gaming group”) where it reads naturally. For readability and consistency across the numerous figures and tables — including all figures in the Supplementary Information — we have retained the concise group labels (occasional-, low-, intermediate-, heavy-gamers) throughout, now to be understood as shorthand for these engagement-based groups; this convention is stated explicitly where the groups are first defined.

Introduction:

- Please consider revising the first sentence of the introduction to reflect that it was Internet Gaming Disorder (not the broader construct of problematic gaming) that was included in Section III as a condition for further study.

Response: We have replaced PG by IGD in the first sentence.

- It may be helpful to include in-text citations in the “host” and “environment” sections (e.g., studies showing relationships between depression and problematic gaming).

Response: We have added in-text citations to the host and environment sentences. For the environment, we now cite Schneider, King & Delfabbro (2017) and Coşa et al. (2023) directly at the relevant sentence; for the host, we cite the existing references on psychopathology and ADHD/impulsivity (e.g., Gao et al., 2022; Koncz et al., 2023) at the point where depression, anxiety, attention problems, and impulsivity are introduced.

- I would recommend removing the word “established” in the subheading.

Response: Done — “established” has been removed from the subheading (now “Modifiable risk and protective factors of PG”).

- Replacing “predicting” with “associated with” may be preferable in the following sentence: “…predicting subsequent symptoms severity is strong.”

Response: Done — the sentence now reads “…associated with subsequent symptom severity is strong.”

- I believe the word “and” is missing here: “…perseverance, sensation seeking.”

Response: Done — corrected to “…perseverance, and sensation seeking.”

- Rewriting for clarity and adding in-text citations here may strengthen the sentence: “impulsivity is associated with difficulties considering alternative solutions, with acting precipitously and ignoring consequences and is recognized as a predictive factor in the development of maladaptive behaviors resulting from poor regulation of positive and negative emotions.”

Response: We have rewritten this sentence for clarity and added citations. It now reads: “Within the UPPS-P framework (Cyders & Smith, 2008), impulsivity reflects a tendency to act precipitously, to overlook alternative courses of action, and to disregard consequences. As such, it is a recognized predictor of maladaptive behaviors arising from poor regulation of positive and negative emotions (Sharma, Markon & Clark, 2014).”

- For rows 110-113, please consider clarifying that this is in the context of problematic gaming.

Response: We have clarified that this passage refers specifically to the context of problematic gaming.

- I would recommend proofreading the manuscript. For example, there appear to be repeated words in row 172. Also, some words appear to be capitalized, such as impulsiveness and family environment in row 198.

Response: We have carefully proofread the manuscript, removed the repeated word(s) (row 172), and corrected the erroneous capitalization of “impulsiveness” and “family environment” (row 198).

- Considering that the presentations of results (in the results section of the manuscript) are stratified according to group, it would be important to specify how many hours of gaming were considered to be occasional for the “occasional” gaming group both in the introduction and methods. Currently, in the introduction, it states “not usually playing,” but it would be important to report a specific number here or clarify how this was determined (it appears this information can be found in the results section, rows 406-208, but it may be helpful to provide this clarification earlier).

Response: We now provide the operational definition earlier, in both the Introduction and the Methods. “Occasional gamers” are participants who reported not usually playing video games at baseline (i.e., answering “no” when asked whether they usually play), and we cross-reference the quantile-based thresholds used for the other groups (low: 1–10 h/week; intermediate: 10–28 h/week; heavy: >28 h/week, mean ≈ 57 h/week).

Methods:

- Please consider changing 3’434 to 3,434

Response: Done — changed to 3,434.

- It may be helpful to include some additional information regarding the procedure (e.g., was the survey delivered on laptops/phones or on paper? Were participants reimbursed? How exactly were the schools/students recruited?).

Response: We have added detail to the procedure and now cite Gentile et al. (2011) and Choo et al. (2010), where the full data-collection procedures are described. The survey was administered as a paper-and-pencil questionnaire in participants’ classrooms by their teachers, with four counterbalanced versions distributed across classes. Schools were recruited as a convenience sample: principals of twelve schools (six primary and six secondary) accepted an invitation to participate, and the classes were selected by each school. Participation was voluntary and unpaid (no reimbursement was provided). We now state this in the Procedure and cite Gentile et al. (2011) and Choo et al. (2010) for the full data-collection procedures.

- The wording around “most” may be slightly confusing for readers in rows 232-233. Please consider clarifying the “most of the cohort was followed” section. If it is referring to participants who were retained over the three-year follow-up period, please explicitly state this.

Response: We have clarified this. The sentence now states explicitly that this refers to participants who were retained across the three-year follow-up period (three annual waves).

- I suggest being more cautious with the wording in rows 243-245, as some DSM-IV pathological-gambling criteria (e.g., chasing losses, committing illegal acts, and relying on others for financial bailouts) were not transferred to IGD.

Response: We agree and have made the wording more cautious. We now note that not all DSM-IV pathological-gambling criteria were transferred to IGD: only nine of the ten items map onto the DSM-5 IGD criteria, and criteria such as chasing losses, committing illegal acts, and financial bailout are DSM-IV-specific and are not represented in our adapted instrument (see Measures and Table S6).

- I would also recommend slightly adjusting the wording for rows 242-243 (perhaps removing the “10-item” part) to better align with the following sentence in the referenced King et al. article: “Prior to the provisional DSM-5 criteria for GD, researchers would often adapt the criteria of other disorders (e.g., pathological gambling in the DSM-IV-TR).”

Response: We have adjusted the wording to align with King et al. We removed the “10-item” descriptor where it created ambiguity and now describe the instrument as adapted from the DSM-IV pathological-gambling criteria, consistent with King et al.’s phrasing.

- Please include a space between “and13%”

Response: Done — a space has been added (“and 13%”).

- After the “measures included in this reanalysis” subheading, it would be helpful to include additional subheadings for the other sections (e.g., an “ethics” subheading for rows 306 -317 and also a “statistical analysis” subheading).

Response: We have added the suggested subheadings: an “Ethics” subheading (former rows 306–317) and a “Statistical analysis” subheading.

- Please correct the typo for “environement”

Response: Done — corrected to “environment” throughout.

- Please change “ensures” to “ensure” in row 324.

Response: Done — changed “ensures” to “ensure.”

- Some additional specific information would be helpful for the statistical analysis section. For example, what statistical package was used, how model fit and significance were evaluated, etc.

Response: We have expanded the Statistical analysis section. All analyses were conducted in R (Version 4.6.1). Specification-curve analysis was implemented with the specr package; partial η² effect sizes and Type-III ANOVAs were computed with the car, effectsize and heplots packages. Linear mixed-effects moderation models were fitted with lme4 (with afex, emmeans and interactions for follow-up tests). Finally, the CLPM and RI-CLPM were estimated with lavaan using MLR estimator with FIML for missing data. Statistical significance was evaluated at α = .05, and effect sizes were interpreted using the conventional benchmarks already reported in the manuscript. Model fits were estimated using AIC and BIC for ANOVAs and mixed-effects models as well as with CFI, RMSEA and SRMR for RI-CLPM models.

- Regarding the section describing the problematic gaming scale, it would be helpful to briefly summarize the 10 items, given that the results show “sneaky” and “deception” as separate symptoms.

Response: We now briefly summarize the ten items of the PG scale in the Measures section and list them in full in Table S2/S6, so that the symptom-level results (e.g., “sneaky” and “deception”) can be readily interpreted.

Results:

- It appears that the number of hours of gaming per week that determines what group a participant is in is different from what is stated in the introduction. Also, regarding the heavy-gaming group, it would be helpful to add the range of hours. For the remaining groups, it would be helpful to add the mean.

Response: We have reconciled the group definitions between the Introduction and the Results so that consistent thresholds are reported throughout. For the heavy-gaming group we now report the range (up to 106 h/week; mean ≈ 57 h/week), and we have added the mean for each remaining group (occasional, low, and intermediate).

Discussion:

- In the discussion or the introduction, it may be beneficial to provide a nuanced discussion of the limitations associated with categorizing participants into named gaming-engagement groups (“occasional, low, intermediate, or heavy”) solely on the basis of hours played.

Response: We have expanded this limitation (currently the third limitation) to discuss more explicitly that classifying participants into named engagement groups solely on the basis of self-reported hours is a simplification that does not capture motivation, functional impairment, or context, and that the heavy-gaming group in particular is likely heterogeneous.

- Regarding the fifth limitation, it may be helpful to consider discussing the use of the TimeLine FollowBack in previous gaming disorder studies (e.g., “A brief internet-delivered intervention for the reduction of gaming-related harm: A feasibility study”) to reduce the concerns stated.

Response: We have added a discussion of the Timeline Followback (TLFB) approach to the fifth limitation, noting that more structured retrospective methods such as TLFB have been used in gaming research (e.g., Park et al., 2020, Computers in Human Behavior Reports) and could mitigate the imprecision of typical-day self-reports of gaming time in future work.

- Please consider including a brief conclusion section (and subheading) at the end of the discussion.

Response: We have added a brief Conclusion section with its own subheading at the end of the Discussion, as follows:

Conclusion. By stratifying adolescents according to their baseline gaming habits, this study shows that the factors most strongly linked to changes in problematic gaming differ across engagement levels: changes in gaming time dominate in occasional gamers, whereas changes in impulse control dominate in heavy gamers. These findings argue against a one-size-fits-all approach and support tailoring prevention and intervention to a young person’s current gaming profile: supporting impulse regulation in heavy gamers and addressing the social integration of an emerging interest in gaming among occasional gamers. More broadly, they underscore that time spent playing is, on its own, an incomplete index of harm, and that future work combining engagement level with measures of motivation and function will be needed to refine these recommendations.

Attachment Submitted filename: PlosOne_ReviewersResponse_R3_CLEAN.docx

# Peer review: Decision Letter 3

**DOI:** 10.1371/journal.pone.0356080.r007

**Authors:** Marc Potenza

31 Aug 2026

Problematic gaming in occasional, low, intermediate or heavy use gamers – not all screen time is created equal - a reanalysis

PONE-D-25-39575R3

Dear Dr. Bediou,

We’re pleased to inform you that your manuscript has been judged scientifically suitable for publication and will be formally accepted for publication once it meets all outstanding technical requirements.

Within one week, you’ll receive an e-mail detailing the required amendments. When these have been addressed, you’ll receive a formal acceptance letter and your manuscript will be scheduled for publication.

An invoice will be generated when your article is formally accepted. Please note, if your institution has a publishing partnership with PLOS and your article meets the relevant criteria, all or part of your publication costs will be covered. Please make sure your user information is up-to-date by logging into Editorial Manager at Editorial Manager® and clicking the ‘Update My Information' link at the top of the page. For questions related to billing, please contact billing support.

If your institution or institutions have a press office, please notify them about your upcoming paper to help maximize its impact. If they’ll be preparing press materials, please inform our press team as soon as possible -- no later than 48 hours after receiving the formal acceptance. Your manuscript will remain under strict press embargo until 2 pm Eastern Time on the date of publication. For more information, please contact onepress@plos.org.

Kind regards,

Marc N Potenza

Academic Editor

PLOS One

Additional Editor Comments (optional):

Reviewers' comments:

Reviewer's Responses to Questions

**Comments to the Author**

Reviewer #4: All comments have been addressed

**********

2. Is the manuscript technically sound, and do the data support the conclusions?

Reviewer #4: Yes

**********

3. Has the statistical analysis been performed appropriately and rigorously?

Reviewer #4: Yes

**********

4. Have the authors made all data underlying the findings in their manuscript fully available?

The PLOS Data policy

Reviewer #4: No

**********

5. Is the manuscript presented in an intelligible fashion and written in standard English?

Reviewer #4: Yes

**********

Reviewer #4: Thank you for addressing the previous comments and for providing thoughtful revisions to the manuscript. I have no further comments.

**********

what does this mean?). If published, this will include your full peer review and any attached files.

If you choose “no”, your identity will remain anonymous but your review may still be made public.

**Do you want your identity to be public for this peer review?**  For information about this choice, including consent withdrawal, please see our Privacy Policy

Reviewer #4: **Yes:**  Jennifer Park

**********

# Editor report: Acceptance letter

**DOI:** 10.1371/journal.pone.0356080.r008

**Authors:** Marc Potenza

PONE-D-25-39575R3

PLOS One

Dear Dr. Bediou,

I'm pleased to inform you that your manuscript has been deemed suitable for publication in PLOS One. Congratulations! Your manuscript is now being handed over to our production team.

At this stage, our production department will prepare your paper for publication. This includes ensuring the following:

* All references, tables, and figures are properly cited

* All relevant supporting information is included in the manuscript submission,

* There are no issues that prevent the paper from being properly typeset

You will receive further instructions from the production team, including instructions on how to review your proof when it is ready. Please keep in mind that we are working through a large volume of accepted articles, so please give us a few days to review your paper and let you know the next and final steps.

Lastly, if your institution or institutions have a press office, please let them know about your upcoming paper now to help maximize its impact. If they'll be preparing press materials, please inform our press team within the next 48 hours. Your manuscript will remain under strict press embargo until 2 pm Eastern Time on the date of publication. For more information, please contact onepress@plos.org.

You will receive an invoice from PLOS for your publication fee after your manuscript has reached the completed accept phase. If you receive an email requesting payment before acceptance or for any other service, this may be a phishing scheme. Learn how to identify phishing emails and protect your accounts at https://explore.plos.org/phishing.

If we can help with anything else, please email us at customercare@plos.org.

Thank you for submitting your work to PLOS One and supporting open access.

Kind regards,

PLOS One Editorial Office Staff

on behalf of

Dr. Marc N Potenza

Academic Editor

PLOS One
